# Stem cells tightly regulate dead cell clearance to maintain tissue fitness

**DOI:** 10.1038/s41586-024-07855-6

**Published:** 2024-08-21

**Authors:** Katherine S. Stewart, Merve Deniz Abdusselamoglu, Matthew T. Tierney, Anita Gola, Yun Ha Hur, Kevin A. U. Gonzales, Shaopeng Yuan, Alain R. Bonny, Yihao Yang, Nicole R. Infarinato, Christopher J. Cowley, John M. Levorse, Hilda Amalia Pasolli, Sourav Ghosh, Carla V. Rothlin, Elaine Fuchs

**Affiliations:** 1 Howard Hughes Medical Institute, Robin Chemers Neustein Laboratory of Mammalian Cell Biology and Development, The Rockefeller University, New York, NY USA; 2 Electron Microscopy Resource Center, The Rockefeller University, New York, NY USA; 3 Departments of Neurology and Pharmacology, Yale School of Medicine, New Haven, CT USA; 4 Departments of Immunobiology and Pharmacology, Yale School of Medicine, New Haven, CT USA; 5 Present Address: Department of Life Sciences, Pohang University of Science and Technology, Pohang, Republic of Korea; 6 Present Address: Department of Discovery Technology and Genomics, Novo Nordisk Research Centre Oxford, Oxford, UK; 7 Present Address: Altos Labs, Cambridge Institute of Science, Granta Park, Cambridge, UK; 8 Present Address: Altos Labs, San Diego, CA USA; 9 Present Address: PrecisionScienta, Yardley, PA USA; 10 Present Address: Molecular Pharmacology Program, Memorial Sloan Kettering Cancer Center, New York, NY USA; 11 Present Address: Cardiovascular Research Group, Temple University, Philadelphia, PA USA

**Keywords:** Cell death and immune response, Apoptosis, Stem-cell niche, Skin stem cells, Nuclear receptors

## Abstract

Billions of cells are eliminated daily from our bodies^1–4^. Although macrophages and dendritic cells are dedicated to migrating and engulfing dying cells and debris, many epithelial and mesenchymal tissue cells can digest nearby apoptotic corpses^1–4^. How these non-motile, non-professional phagocytes sense and eliminate dying cells while maintaining their normal tissue functions is unclear. Here we explore the mechanisms that underlie their multifunctionality by exploiting the cyclical bouts of tissue regeneration and degeneration during hair cycling. We show that hair follicle stem cells transiently unleash phagocytosis at the correct time and place through local molecular triggers that depend on both lipids released by neighbouring apoptotic corpses and retinoids released by healthy counterparts. We trace the heart of this dual ligand requirement to RARγ–RXRα, whose activation enables tight regulation of apoptotic cell clearance genes and provides an effective, tunable mechanism to offset phagocytic duties against the primary stem cell function of preserving tissue integrity during homeostasis. Finally, we provide functional evidence that hair follicle stem cell-mediated phagocytosis is not simply redundant with professional phagocytes but rather has clear benefits to tissue fitness. Our findings have broad implications for other non-motile tissue stem or progenitor cells that encounter cell death in an immune-privileged niche.

## Abstract (web summary)

The spatiotemporal activation of phagocytosis by hair follicle cells is orchestrated by lipids released from surrounding apoptotic cells and retinoids released by healthy cells.

## Main

In developmental, homeostatic, and pathological contexts, professional (immune cells) and non-professional (epithelial, mesenchymal and neuronal cells) phagocytes detect and clear apoptotic corpses—a process called efferocytosis. Failure to do so results in secondary necrosis, causing inflammatory and/or degenerative pathologies^1–4^. Studies on phagocytic cells have shown that upon engagement of phagocytic receptors, the ELMO–DOCK–RAC pathway is activated, eliciting actin cytoskeleton rearrangement and facilitating uptake of apoptotic bodies^2–4^. Subsequent digestion of corpses occurs via phagosome maturation and fusion with lysosomes, during which corpse-derived materials are degraded^2–5^.

**Fig. 1:**
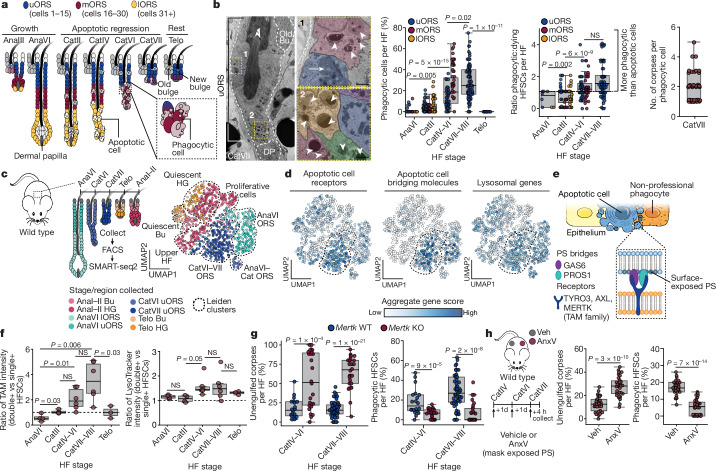
Apoptotic corpses are transiently cleared by neighbouring HFSCs in late catagen. **a**, Schematic of the hair cycle depicting inactive or old HFSC niche (grey) and active or current niche in uORS. Middle ORS (mORS) and lORS die in catagen. Ana, anagen; Cat, catagen; Telo, telogen. **b**, Left, ultrastructure of engulfed corpses (white arrowheads) within catagen uORS. Outlined regions are magnified and pseudocoloured on the right. White arrows denote apoptotic cells and dashed lines denote dermo-epithelial border. Graphs show quantification of phagocytic cells, ratio of phagocytic to apoptotic cells per hair follicle (HF) (*n* = 25 hair follicles, 4 mice per stage (AnaVI; Telo); or *n* = 38 hair follicles (CatII), 51 hair follicles (CatIV–VI), 45 hair follicles (CatVII–VIII), 6 mice per stage) and number of corpses per phagocytic cell (*n* = 7 hair follicles). Bu, bulge; DP, dermal papilla. Scale bars, 2 μm. **c**, Left, single-cell RNA sequencing (scRNA-seq) strategy for hair follicle epithelial cells. Right, uniform manifold approximation and projection (UMAP) representation and clustering of transcriptomes across the hair cycle. HG, hair germ. **d**, UMAP representations coloured by aggregate gene-set score with catagen cluster outlined. **e**, Schematic depicting apoptotic cell recognition by neighbouring phagocyte. PS, phosphatidylserine. **f**, Expression of TAM-family phagocytic receptors (left) and lysosomal genes (right) in *Sox9-creER*^*+*^*;R26-Brainbow2.1* fluorophore HFSCs that are double-fluor-positive (double+) or single-fluor-positive (single+) per mouse. *n* = 3 (Telo), *n* = 4 (AnaVI, CatII), or *n* = 6 (CatIV–VI, CatVII–VIII) mice per stage. **g**, Quantification of unengulfed corpses (left) and phagocytic HFSCs (right) from wild-type (WT) versus *Mertk*-knockout (KO) mice. *n* = 20 WT, *n* = 26 *Mertk*-KO (CatIV–VI) and *n* = 53 WT, *n* = 28 *Mertk*-KO (CatVII–VII) hair follicles quantified, 3 mice per stage. **h**, Strategy (left) using recombinant annexin V (AnxV) to block corpse-exposed phosphatidylserine during catagen (contralateral vehicle (Veh) injections). Quantifications of unengulfed corpses (middle) and phagocytic HFSCs (right). Ten hair follicles per mouse, *n* = 3 mice per condition. 1d, 1 day. Pairwise independent two-sided Student’s *t*-tests, *P* values indicated. NS, not significant (*P* > 0.05). In box plots, the centre line is the median, box edges delineate first and third quartiles and whiskers extend to 1.5× the inter-quartile range. Further details on statistics and reproducibility in Methods. See Extended Data Figs. 1–3 for additional supporting experiments and Supplementary Table 1 for differential gene expression analysis. Source data

In contrast to immune phagocytes, most non-professional phagocytes are non-motile and restrict their phagocytosis to a brief and highly focused diversion from their other dedicated tissue functions^2,4,6^. Here we investigate the underlying mechanisms involved. To do so, we focus on the epithelial stem cells of the mouse hair follicle. Following every synchronized bout of follicle regeneration and hair production (anagen), the entire hair follicle beneath the stem cell compartment is destroyed by a process of apoptosis and phagocytosis (catagen), which initiates at the very base of the follicle and works its way up to the stem cell niche^7–10^ (Fig. 1a). We dissect how the phagocytic process is tightly regulated to maintain stem cell preservation and tissue fitness. We address the benefits of engulfing corpses to the stem cells that survive the destructive phase, and the consequences when this mechanism malfunctions. In doing so, we uncover insights into a process that occurs in nearly all tissues and unravel a reversible, tunable regulatory mechanism within stem cells that has important implications for the fields of cell death and tissue fitness.

## Hair follicle stem cells can engulf many corpses

Quiescent (telogen) hair follicle stem cells (HFSCs) that are responsible for propagating regenerative hair cycles reside within the upper outer root sheath (uORS) of the hair follicle in an anatomical region called the bulge^7,8^. A new growth phase is launched when crosstalk between HFSCs and underlying specialized mesenchymal cells (dermal papilla) reaches a threshold, prompting HFSCs to briefly proliferate and generate the short-lived progeny that fuel hair growth^11,12^. As the new hair follicle grows and pushes the dermal papilla downward, its uORS returns to quiescence, setting aside a new pool (bulge) of HFSCs for the next hair cycle, while the short-lived progeny fuel production of the hair at the base (bulb) of the mature hair follicle^13^.

**Extended Data Fig. 1:**
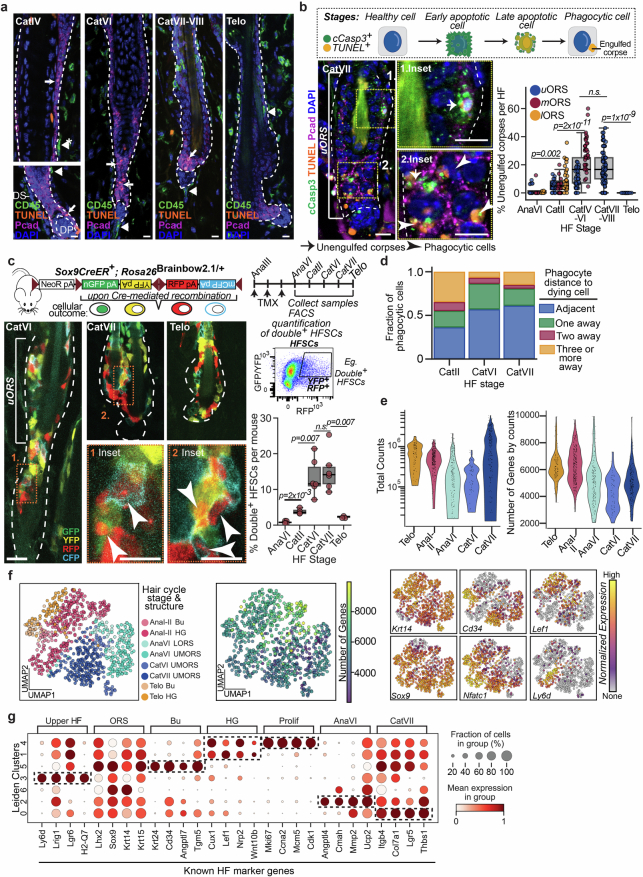
Apoptotic corpses are cleared by neighbouring HFSCs in catagen. **a**, Sagittal wild type (WT) skin sections across the catagen (Cat)- telogen (Telo) hair cycle. CD45, dermal immune cells (white arrowheads); TUNEL, DNA damage in apoptotic corpses (white arrows); P-cadherin (Pcad), HF outer root sheath (ORS); DAPI, DNA. Dashed lines, dermo-epithelial border; asterisk denotes TUNEL^+^ de-nuclearization of hair shaft lineages as part of differentiation; scale bar 10 μm. Images are representative of 25-30 follicles across 3 mice per stage. **b**, Top, Schematic delineating timing of apoptotic markers. Cleaved caspase 3 (cCasp3) marks early apoptotic cells and late apoptotic corpses, while TUNEL marks late apoptotic corpses both unengulfed and engulfed. Bottom left, Sagittal WT skin sections of engulfed apoptotic corpses (cCasp3^+^TUNEL^+^) encased by upper ORS (*u*ORS) catagen cells (cell membrane delineated by P-cadherin). Boxed regions, magnified/inset at right. Dashed lines, dermo-epithelial border; Scale bar 10 μm. Bottom right, Quantification of unengulfed dying cells (top) and ratio of phagocytic to apoptotic cells (bottom) per hair follicle (HF) across the hair cycle. (*n* = 25 HFs, 4 mice per stage (AnaVI; Telo); or *n* = 38 HFs (CatII), 51HFs (CatIV-VI), 45 HFs (CatVII-VIII), 6 mice per stage). **c**, Top, Experimental schematic to identify phagocytic HFSCs. Bottom left, Representative immunofluorescence images of single fluor^+^ (containing no corpses) and double fluor^+^ (phagocytic/corpse-containing, white arrowhead) HFSCs in the *u*ORS. Boxed regions, magnified insets at bottom. Scale bar 20 μm. Bottom right, FACS was used to quantify single and double+ cells across the hair cycle. *n* = 4 each (AnaVI, CatII); *n* = 6 each (CatIV-VI, CatVII-VIII) and *n* = 3 (Telo) mice. **d**, Quantifications across the hair cycle of phagocytic cell position with respect to nearest apoptotic cell. *n* = 414 cells across 4-6 mice per stage. **e**, Total counts per cell (left), number of genes detected per cell (right) for scRNA-sequencing transcriptomes across the hair cycle. Telo, telogen; AnaI-II, early anagen; AnaVI, late anagen; CatVI-VII, mid-late catagen. **f**, Left, Uniform manifold projection (UMAP) representation of scRNA-seq data coloured by anatomic location (via FACS antibody markers) and hair cycle stage. Bu, bulge; HG, hair germ; LORS, lower outer root sheath (ORS); UMORS, upper-middle ORS. Middle, UMAP representation of number of genes detected per cell, colourbar, right. Left, UMAPs of relative expression levels (log2[TPM + 1]) of selected marker genes. ORS markers: *Krt14, Sox9*; Bu hair follicle stem cell (HFSC) markers: *Cd34, Nfatc1*; HG progenitor marker: *Lef1*; Upper HF marker: *Ly6d*. **g**, Dot plot representation of marker gene analysis across Leiden clusters. Horizontal rows are grouped by Leiden cluster; vertical columns represent marker genes (grouped by reported function/location in hair cycle; Prolif, proliferation). Quantifications, pairwise independent Student’s *t*-tests (2-sided), p-values indicated. n.s. not significant (p > 0.05). Data as box-and-whisker plots; box: first-to-third quartiles and median, whiskers 1.5× inter-quartile range. More details on statistics and reproducibility can be found in the Methods section. Source data

Prior studies on the start of catagen have shown that when apoptotic cell death becomes widespread in the hair bulb, the lower outer root sheath (lORS) cells and not the CD45^+^ professional phagocytes clear the corpses^10^. What happens when apoptosis reaches the stem cell niche is unclear. After corroborating that HFSCs in the uORS experience minimal cell death during early catagen, we monitored apoptosis into late catagen by cleaved caspase-3 (cCasp3^+^; marking early apoptosis) and DNA damage (TUNEL^+^; marking late death and engulfment stages). Phagocytic hair follicle cells were in equal proportion to apoptotic cells early in catagen (CatII) but increased to roughly twice as many by late catagen (Fig. 1b). At the end of catagen, engulfed apoptotic corpses were found entirely within the hair follicle stem and progenitor populations responsible for driving the next hair cycle. These cells appeared healthy, each displaying TUNEL^+^ condensed apoptotic bodies within their cytoplasm, indicative of phagocytosis (Extended Data Fig. 1a,b).

We verified the ability of the stem cells to clear apoptotic corpses by devising a strategy to detect bona fide phagocytic cells within catagen-phase hair follicles. By activating Cre recombinase in mid-growth hair follicles of *Sox9-creER*^*+*^;*R26-Brainbow2.1*^*fl/+*^ mice, we specifically labelled the uORS compartment with one of four fluorophores and then traced labelled cells across the hair cycle. Confocal microscopy and flow cytometry quantification revealed engulfed apoptotic bodies containing one fluorophore encased by otherwise healthy HFSCs expressing a different fluorophore (Extended Data Fig. 1c).

In late catagen, most phagocytic cells were adjacent to dying neighbours (Extended Data Fig. 1d), suggesting that in contrast to macrophages, non-motile HFSCs require close proximity to the dying cell. Moreover, uORS cells appeared to be highly efficient phagocytes, as ultrathin images of late catagen follicles revealed an average of 2 to 3 apoptotic corpses per uORS cell, suggestive of multiple rounds of engulfment (Fig. 1b). As surviving uORS cells comprise the HFSCs used for the next round of tissue regeneration, the data suggest an advantage to their eating.

## Catagen-regulated phagocytosis in HFSCs

Whereas early catagen lORS cells die, some late catagen HFSCs are spared and must silence efferocytosis once the apoptotic wave subsides so that they can return to their normal function of fuelling the next hair cycle. To explore the spatiotemporal regulation of efferocytosis, we first performed single-cell transcriptomic profiling of the uORS across the hair cycle, identifying 7 Leiden cell clusters (Fig. 1c and Extended Data Fig. 1e–g). To determine the transcriptional shifts that accompany the transition from hair follicle growth to regression, we performed gene set enrichment analysis on differentially expressed genes from the end of the growth phase (AnaVI) to mid-late destructive phase (CatVI–VII) (Supplementary Table 1).

**Extended Data Fig. 2:**
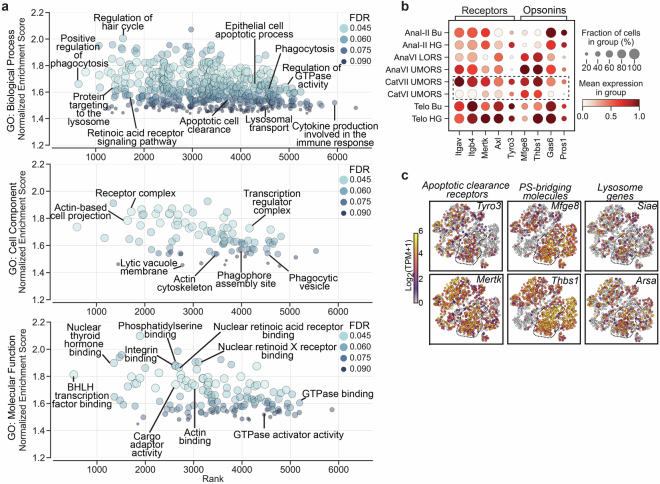
Gene set enrichment identifies pathways related to apoptotic cell clearance upregulated in catagen HFSCs. **a**, Visual representation of all gene sets identified as upregulated in catagen versus anagen (FDR < 0.1), broken by Gene Ontology (GO) category. Gene set enrichment analysis (GSEA) ranks gene set terms by position at which maximum enrichment occurs (horizontal axis), and by normalized enrichment score (vertical axis). Dot colour and size reflects the False Discovery Rate (FDR) calculated for each gene set (legend inset). Selected gene sets related to apoptotic cell clearance are annotated. See Supplementary Tables 1 and 2 for full lists. **b**, Dot plot representation of selected apoptotic cell recognition receptors and phosphatidylserine-bridging genes (vertical columns) across hair cycle stages (horizontal rows). Each dot represents the fraction of cells (size) and normalized mean expression (colour) of a single gene across the hair cycle grouped single cell transcriptomes. **c**, UMAP representation of single-cell transcriptomes coloured by the relative expression levels (log2[TPM + 1]) of example genes (relating to aggregate gene sets shown in Fig. 1d) from apoptotic cell receptors (left), apoptotic cell bridging molecules (middle) and lysosomal genes (right). The catagen cluster is outlined.

Phagocytic cells display receptors that bind to phosphatidylserine exposed on the apoptotic cell surface^4^. This can happen either directly or through engaging bridging molecules. Comparing genes enriched in catagen-phase stem (integrin α6^+^CD34^hi^) and progenitor (integrin α6^+^CD34^lo^^w^) uORS cells relative to the late-anagen stage, we found a significant enrichment of gene ontology terms related to the phagocytic state (Extended Data Fig. 2a). To better pinpoint the cell population(s) associated with a transcriptomic increase in phagocytic activity, we created aggregate gene set scores for these terms and visualized them on the UMAP data. Intriguingly, HFSCs in the catagen phase uORS were strongly enriched for transcripts encoding apoptotic cell clearance receptors and bridging molecules, with a more modest increase in the lysosomal pathway (Fig. 1d,e). Included in this cohort were genes encoding several members of the TYRO3/AXL/MERTK (TAM)-family receptor and integrin receptor pathways of apoptotic cell recognition and tethering, including *Tyro3*, *Mertk* and *Itgav*, as well as genes encoding the phosphatidylserine-bridging proteins *Gas6*, *Pros1*, *Mfge8* and *Thbs1* (Extended Data Fig. 2b,c and Supplementary Table 1).

**Extended Data Fig. 3:**
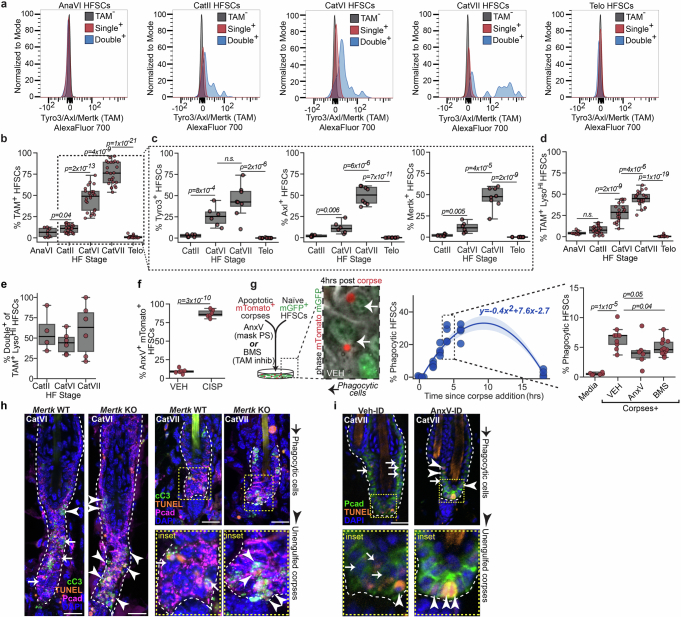
TAM-family receptor pathway for apoptotic cell engulfment is expressed in HFSCs during apoptotic regression. **a**, Representative FACS plots showing normalized counts of *Tyro3/Axl/Mertk* (TAM)-family receptors expression on *Sox9CreER*^*+*^*; Brainbow2.1* single-fluor^+^ versus double-fluor^+^ HFSCs isolated across the hair cycle. Experimental design as in Extended Data Fig. 1c. Ana, anagen; Cat, catagen; Telo, telogen. *n* = 4-6 mice analyzed per stage. Data is quantified in Fig. 1f. **b**, Percentage of TAM-family^+^ HFSCs per mouse across the hair follicle (HF) stage. *n* = 8 (AnaVI), *n* = 20 (CatII), *n* = 23 (CatVI), *n* = 24 (CatVII), and *n* = 14 (Telo) mice. **c**, Percentage of FACS-isolated HFSCs expressing each individual TAM-family member (Tyro3, left; Axl, middle; Mertk, right) during catagen. *n* = 6 each (CatII, CatVI), *n* = 8 (CatVII), and *n* = 10 (Telo) mice analyzed. **d**, Percentage of HFSCs which are TAM^+^ and lysosome^high^ (components of phagocytic program) across the hair cycle. *n* = 8 (AnaVI), *n* = 20 (CatII, CatVI), *n* = 22 (CatVII), and *n* = 14 (Telo) mice. **e**, Percentage of FACS-purified *Sox9CreER*^*+*^ *Brainbow2.1* fluorophore HFSCs that are TAM^+^ and lysosome^high^ and contain an apoptotic corpse (double^+^) per mouse across the hair cycle, *n* = 4 (CatII) and *n* = 8 each (CatVI, CatVII) mice. **f**, FACS-based percentage of mTomato^+^ HFSCs undergoing apoptosis (AnnexinV^+^) in preparation of corpses/corpse-conditioned media. CISP, cisplatin. *n* = 6 replicates. **g**, Left, Experimental strategy to expose naïve HFSCs to corpses directly, with representative image (middle left) showing engulfed mTomato^+^ apoptotic corpses inside GFP^+^ HFSCs 4 h post corpse addition. Middle right, Time-course quantification of total HFSCs that contain a corpse in vitro. *n* = 4 experimental replicates per time point. Shaded bands, 95% confidence interval. Note, data from 0-6 h post corpse exposure is replicated in Fig. 3c to contrast with nuclear RXRa upregulation. Right, Quantification of corpse-containing HFSCs; engulfment requires phosphatidylserine exposure (blocked by addition of AnnexinV [AnxV]) and TAM-family activity (inhibited by addition of BMS-777607 [BMS]). *n* = 6 (Media), *n* = 9 (Corpses+VEH), *n* = 6 (Corpses+AnxV), and *n* = 11 (Corpses+BMS) experimental replicates. **h**, Sagittal sections of wild type (WT) and constitutive *Mertk* knockout (KO) skin; boxed regions are magnified at bottom; cC3, cleaved caspase 3; Pcad, p-cadherin; scale bar 20 μm. **i**, Sagittal sections of contralateral intradermal (ID) injections of vehicle (Veh) or annexinV (AnxV) to mask corpse exposed phosphatidylserine during catagen in WT mice; boxed regions are magnified at bottom; Pcad, p-cadherin; scale bar 10 μm. For (h) and (i) data are quantified in Fig. 1g,h. Quantifications, pairwise independent Student’s *t*-tests (2-sided), p-values indicated. n.s. not significant (p > 0.05). Data as box-and-whisker plots; box: first-to-third quartiles and median, whiskers 1.5× inter-quartile range. More details on statistics and reproducibility can be found in the Methods section. Source data

If these transcriptional differences are physiologically relevant to the phagocytic state, their encoded proteins should be enriched specifically on the surface of outer root sheath (ORS) cells that engulf apoptotic cells. To test this, we repeated our Brainbow experiment, this time performing flow cytometry with antibodies against individual TAM-family members as well as a pan-TAM antibody. Surface expression of TAM-family members spiked during late catagen, but was low or absent at other phases of the hair cycle. A subset of these TAM-family^+^ HFSCs also displayed an expanded lysosomal compartment, indicative of apoptotic corpse degradation within phagolysosomes (Fig. 1f and Extended Data Fig. 3a–e). Thus, although some phagocytic proteins have additional biological functions, phagocytic protein and transcript expression in the hair cycle correlated well with the pronounced phagocytic activity seen in catagen HFSCs.

By monitoring the entire destructive phase of the hair cycle, a striking spatiotemporal relationship emerged between phagocytic activity and the presence of dying neighbours. To test whether healthy ORS cells activate phagocytosis by directly sensing dying neighbours, we first turned to an in vitro system, exposing healthy HFSCs to corpses derived from a culture treated with an apoptotic agent (Extended Data Fig. 3f,g). As expected, naive HFSCs responded by engulfing the corpses. However, when HFSCs were pretreated with BMS-777607 to inhibit TAM-family receptor activity, or with recombinant annexin V to mask exposed phosphatidylserine on apoptotic corpses, engulfment was impaired, underscoring the importance of these receptors in the process.

Interrogating the in vivo relevance of our findings, we ablated *Mertk* in mice, and also blocked exposed phosphatidylserine with intradermally injected annexin V (Fig. 1g,h and Extended Data Fig. 3h,i). Both measures delayed apoptotic corpse clearance, with more unengulfed apoptotic cells and fewer phagocytic ORS cells compared with controls. The effects were most potent in late catagen, in parallel with the elevated phagocytic programme.

## RXRα, a master regulator of phagocytosis

**Fig. 2:**
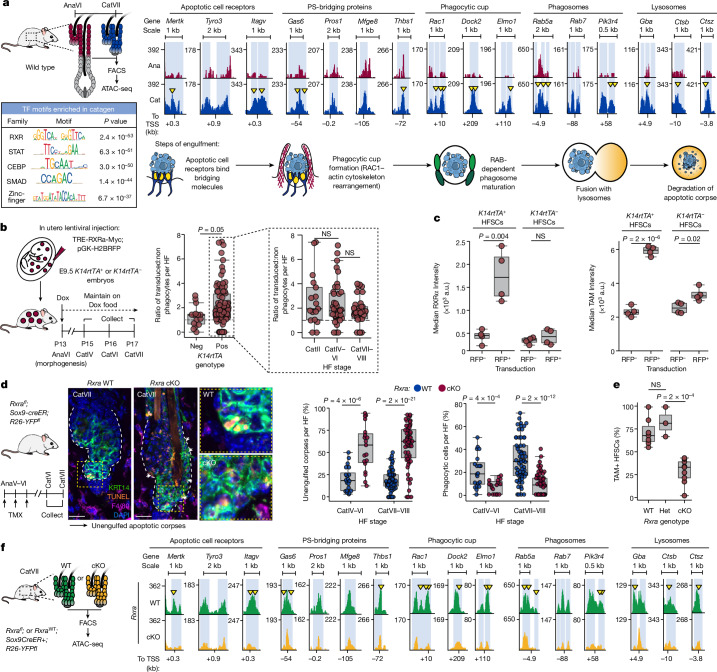
RXRα is a master regulator of the phagocytic hair follicle stem and progenitor cell state. **a**, Left, strategy to profile HFSCs in late anagen (AnaVI) versus late catagen (CatVII) by ATAC-seq (top). Motif enrichment analysis of catagen-accessible peaks (bottom). Right, replicate-pooled ATAC peak tracks for enhancers associated with phagocytic genes in catagen versus anagen. Phagocytic genes are grouped according to engulfment step as shown in the schematic. Differential peaks are highlighted in light blue and yellow arrowheads point to RXR-family-bound footprints. Peak tracks in reads per genome coverage. TF, transcription factor; TSS, transcription start site. **b**, Left, in vivo inducible RXRα expression strategy. Right, quantification of untransduced versus transduced phagocytic cells. *n* = 15 hair follicles (*K14-rtTA*^−^), *n* = 90 hair follicles (*K14-rtTA*^+^), 4 mice per genotype. E indicates embryonic day and P indicates postnatal day. Dox, doxycycline. **c**, Quantification of RXRα (left) and TAM-family (right) expression in FACS-purified *K14-rtTA*^−^ controls and *K14-rtTA*^+^ experimental HFSCs. Data are paired, with untransduced (RFP^−^) and RXRα-overexpressing (RFP^+^) cells co-occurring, *n* = 4 mice per genotype. **d**, Left, strategy to ablate *Rxra* in catagen ORS. Middle, sagittal sections of late catagen *Rxra* wild-type (*Rxra*^*fl/fl*^*;R26-YFP*^*fl/+*^) or *Rxra* conditional-knockout (cKO) (*Sox9-creER*^*+*^*;Rxra*^*fl/fl*^*;R26-YFP*^*fl/+*^) skins. Outlined regions are magnified on the right. Right, quantification of unengulfed corpses and of phagocytic HFSCs per hair follicle. Ten hair follicles analysed per mouse, *n* = 6 mice per genotype. TMX, tamoxifen. Scale bars, 20 μm. **e**, Percentage of FACS-isolated TAM-family^+^ HFSCs per mouse for *Rxra* wild-type, heterozygous mutant (Het) and cKO. *n* = 7 wild type, *n* = 3 heterozygote and *n* = 9 cKO mice. **f**, ATAC-seq pooled replicate peak tracks covering enhancers in **a** for FACS-isolated *Rxra*-WT or *Rxra*-cKO CatVII HFSCs, scaled, normalized and annotated as in **a**. Pairwise independent (**b**,**d**,**e**) or dependent (**c**) two-sided Student’s *t*-tests, *P* values indicated. In box plots, the centre line is the median, box edges delineate first and third quartiles and whiskers extend to 1.5× the inter-quartile range. Further details on statistics and reproducibility in Methods. See also Extended Data Figs. 4 and 5 for additional supporting experiments. Source data

**Extended Data Fig. 4:**
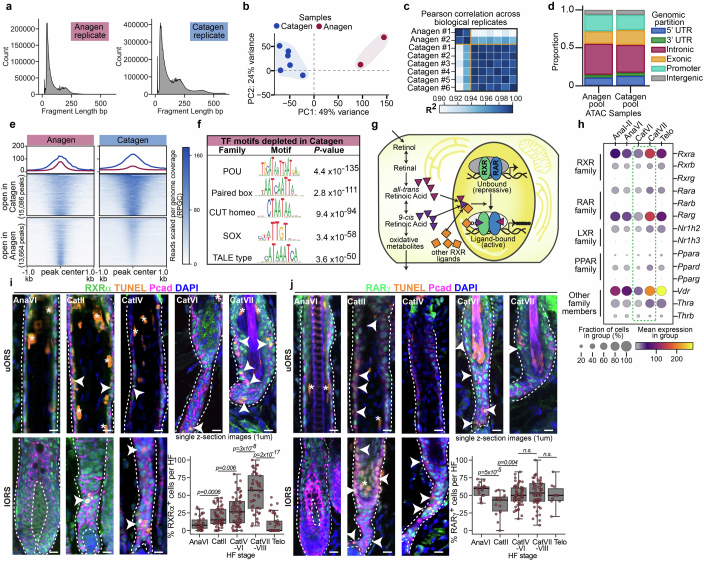
RXR and RAR family members are expressed in proximity to corpses during apoptotic regression. **a**, Representative histograms of fragment lengths in base pairs (bp) detected per biological replicate for each hair cycle stage examined by ATAC-seq. Nucleosome-free, mono- and di-nucleosome peaks were identifiable for all replicates analysed. **b**, Principal component analysis on reads mapped to peaks separates ATAC-seq biological replicates by hair cycle stage; *n* = 2 (anagen) and *n* = 6 (catagen) **c**, Pearson correlation (R^2^) values for reads mapped to peaks across replicates. **d**, Bar graph of peaks partitioning across the genome. **e**, Heatmap showing all peaks with significantly altered accessibility between anagen and catagen replicates. Accessibility signal for pooled replicate samples is normalized for fraction of reads in peaks, and scaled to reads per genome coverage (RPGC) (colourbar legend, right). Each row corresponds to a detected peak, centered and extended +/− 1 kb. Peaks are clustered based on differential accessibility, and summarized as the mean accessibility signal per region in a blue line (peaks gaining accessibility in catagen) or a pink line (peaks losing accessibility in catagen) in the graph at top. **f**, Motif enrichment analysis of the 13,664 peaks losing accessibility in catagen replicates. See Supplementary Table 3 for full list. Quantification via Binomial test (two-tailed, confidence interval 95%). **g**, Schematic depicting retinoid X receptor (RXR) and retinoic acid receptor (RAR) transcription factor signaling. **h**, Dot plot summarizing non-steroidal nuclear hormone receptor family expression across the hair cycle from single cell transcriptomic data. **i, j**, Sagittal skin sections of RXRα^+^ cells (**i**) and RARγ^+^ cells (**j**) within wild type skin from the end of anagen growth phase (AnaVI) throughout catagen (Cat). Dashed line denotes epithelial-dermal border; asterisk denotes TUNEL^+^ de-nuclearization of hair shaft lineages as part of differentiation. Pcad, p-cadherin. Scale bar 15 μm. Quantifications, inset. RXRα+: *n* = 35 HFs (AnaVI), 58 HFs (CatII), 62 HFs (CatIV-VI), 49 HFs (CatVII-VIII) and 45 HFs (Telo) across 8 mice. RARγ+: *n* = 21 HFs (AnaVI), 17 HFs (CatII), 58 HFs (CatIV-VI), 53 HFs (CatVII-VIII) and 10 HFs (Telo) across 6 mice. Quantifications, pairwise independent Student’s *T*-Tests (2-sided), p-values indicated. n.s. not significant (p > 0.05). Data as box-and-whisker plots; box: first-to-third quartiles and median, whiskers 1.5X inter-quartile range. More details on statistics and reproducibility can be found in the Methods section. Source data

The transient nature of the phagocytic programme in these stem cells distinguished it from the one that occurs in professional phagocytes whose primary mission is to clear dead cells. To understand how this phagocytic process is dynamically regulated, we profiled the chromatin landscape as HFSCs progressed from the end of anagen into late catagen. Using an assay for transposase-accessible chromatin by high-throughput sequencing (ATAC-seq) coupled with differential peak analysis, we identified two sets of dynamic chromatin peaks: those which closed upon catagen entry, and those which became accessible (Extended Data Fig. 4a–f). In proximity to catagen-opening peaks were genes encoding apoptotic cell receptors and soluble phosphatidylserine-bridging molecules, as well as proteins involved in phagocytic cup formation and phagolysosome maturation (Fig. 2a).

Seeking candidate transcription factors that might mediate this dynamic chromatin accessibility, we performed motif enrichment analysis for peaks that opened in catagen ORS cells. The most highly enriched motif belonged to the retinoid X receptor (RXR) family (Fig. 2a). Indeed, unbiased transcription factor footprint analysis of the newly accessible phagocytic enhancer regions pinpointed RXR family binding (Fig. 2a, yellow arrowheads), suggesting their direct and dynamic regulation of the phagocytic programme. The most enriched RXR-family motif among catagen-specific peaks was a direct-repeat 2 (DR2) motif composed of hexameric RXR binding motifs separated by 2 nucleotides. Although RXRs can serve as obligate heterodimeric partners of many different nuclear receptors, the DR2 motif has been implicated in RXR heterodimerization with retinoic acid receptors^14,15^ (RARs) (Extended Data Fig. 4g). Moreover, upon surveying expression of the nuclear receptor superfamily across the hair cycle, *Rxra* and *Rarg* stood out as peaking in catagen (Extended Data Fig. 4h–j).

**Extended Data Fig. 5:**
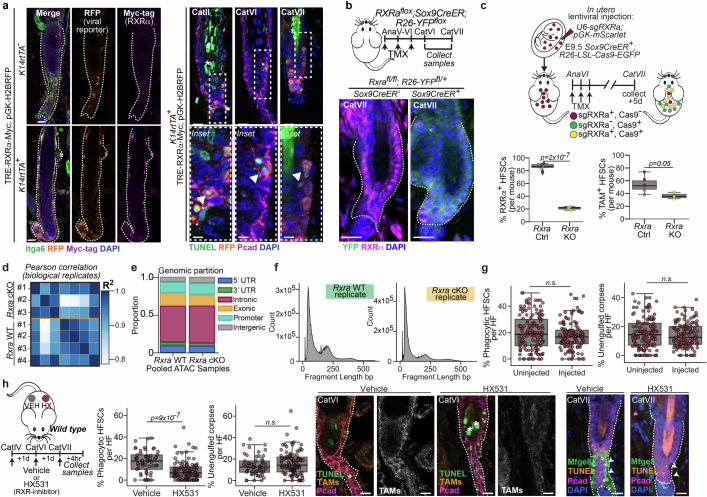
RXRα regulates the apoptotic cell clearance program in vivo. Source data **a**, Sagittal skin sections of *K14rtTA* mice injected with doxycycline (DOX)-inducible *TRE-Rxra-Myc*-tagged lentivirus containing constitutive H2B-RFP infection reporter (TRE-RXRa-Myc;pGK-H2BRFP). Left, DOX treatment induces Myc-tagged RXRα expression only in *K14rtTA*^+^ animals. Scale bar 20 um. Right, *K14rtTA*^+^ RFP^+^ (RXRα-high) cells more often contain TUNEL^+^ corpses than non-transduced (RFP^−^) neighbouring cells. Scale bar 20 μm. Insets, magnified regions of the white dashed box region. Scale bar 10 um. Images representative of 10-20 HFs per mouse, *n* = 3 mice per genotype per catagen stage. **b**, Top, Experimental strategy to ablate *Rxra* in catagen. Tamoxifen, TMX. Bottom, Sagittal skin sections of *Rxra* wild type (WT, *Sox9CreER*^*−*^) and conditional knockout (cKO, *Sox9CreER*^*+*^) show loss of RXRα in YFP^+^ cKO. Scale bar 20 um. Images representative of 10-20 HFs per mouse, *n* = 3 mice per genotype. **c**, Top, Strategy to mosaically knockout (KO) RXRα by lentiviral injection of sgRNA against *Rxra* (sgRXRα) with mScarlet reporter into *Sox9CreER*^*+*^*; R26-floxed-Cas9-EGFP* mice. Bottom, Percentages of RXRα^+^ HFSCs (left) and TAM^+^ HFSCs (right) per mouse. *n* = 3 mice. **d**, Pearson correlation (R^2^) values for *Rxra* WT and cKO ATAC-seq replicates. **e**, Bar graph of peaks partitioning across the genome. **f**, Representative histograms of fragment lengths in base pairs (bp) for *Rxra* WT and cKO HFSCs. **g**, Percentages of phagocytic HFSCs (left) and unengulfed corpses (right) per late catagen HF (CatVII), either uninjected or intradermally injected with vehicle. *n* = 149 HFs (uninjected), *n* = 137 HFs (injected) across 4 mice per condition. **h**, Left, Strategy to transiently inhibit RXR-family transcriptional activity by HX531 intradermal injection. Contralateral backskin was injected with vehicle (Veh) control. Middle, Percentages of corpse-containing HFSCs (left) and unengulfed corpses (right) per HF. *n* = 24 HFs (HX531) and *n* = 20 HFs (Vehicle); across 3 mice. Right, Sagittal sections of contralateral vehicle- and HX531-injected back skin stained as indicated. Asterisk denotes hair shaft keratinization. Images are representative of *n* = 10-12 HFs per mouse, *n* = 4 mice per staining panel. Scale bar 20 μm. Quantifications, pairwise independent Student’s *t*-tests (2-sided), p-values indicated. n.s. not significant (p > 0.05). Data as box-and-whisker plots; box: first-to-third quartiles and median, whiskers 1.5× inter-quartile range. More details on statistics and reproducibility can be found in the Methods section.

If RXRα is a dynamic regulator of catagen-triggered phagocytosis, changing its levels in cells should affect this step. To test this, we used in utero lentiviral delivery to exclusively transduce skin progenitors of *K14-rtTA* embryos with a doxycycline-inducible transgene encoding RXRα, and a constitutive RFP as transduction control. We then administered doxycycline at the end of the first anagen and followed the fate of elevated RXRα ORS cells during catagen. Congruent with a role for RXRα signalling in induction and/or maintenance of this transient phagocytic phase, catagen ORS cells with elevated RXRα more frequently contained engulfed apoptotic bodies than neighbouring untransduced cells (Fig. 2b and Extended Data Fig. 5a). Relative to controls, HFSCs with elevated RXRα also displayed significantly higher levels of TAM-family receptors, consistent with enhanced phagocytic ability (Fig. 2c).

Performing the converse studies, we eliminated RXRα specifically from catagen-phase HFSCs by administering tamoxifen to *Sox9-creER;R26-YFP*^*fl*^*;Rxra*^*fl*^ mice at the end of anagen. In the absence of RXRα, catagen hair follicles initiated apoptosis normally, consistent with prior studies reporting that dermal papilla-generated TGFβ has the apoptosis-initiating role^9,10^ (Extended Data Fig. 5b). Without RXRα, however, fewer catagen-phase HFSCs displayed engulfed corpses and interstitial spaces were littered with apoptotic debris that disintegrated via secondary necrosis (Fig. 2d). RXRα-deficient HFSCs also did not upregulate TAM-family receptor expression in late catagen (Fig. 2e), further demonstrating that these catagen-phase ORS cells had ceased functioning as non-professional phagocytes.

To assess whether RXRα regulates phagocytic receptor expression cell autonomously, we mosaically infected skin progenitors of *Sox9-creER;R26-LSL-Cas9-EGFP* embryos with a lentivirus harbouring an *Rxra-*targeting single guide RNA (sgRNA) and a mScarlet reporter, and administered tamoxifen at the end of the first postnatal anagen. Catagen-phase mScarlet^+^EGFP^+^ ORS cells, which had received both sgRNA and activated Cas9, were largely deficient for RXRα protein whereas single positive cells containing either active Cas9 or sgRNA maintained high RXRα levels. As measured by flow cytometry, the catagen ORS cells that were RXRα-deficient selectively displayed a paucity of surface TAM-family receptors (Extended Data Fig. 5c). Together, these data underscored the importance of catagen-phase RXRα in cell-autonomously activating a phagocytic programme in HFSCs in response to apoptotic neighbours.

Given the role of RXRα as a nuclear receptor, we next addressed whether RXRα is functionally important for opening the dynamic enhancer peaks that emerge during the transition from anagen to catagen. ATAC-seq and differential peak analyses of late catagen-phase HFSCs isolated by fluorescence-activated cell sorting (FACS) revealed that upon *Rxra* ablation, more than 8,000 peaks altered their chromatin accessibility, and nearly half of these showed a dependency on RXRα for accessibility (Extended Data Fig. 5d–f). Notably, the enhancer peaks linked to apoptotic cell clearance genes were among the RXRα-dependent peaks which gained accessibility during catagen of the wild type (Fig. 2f).

The importance of RXR signalling in transcriptionally regulating the apoptotic clearance machinery was bolstered by transiently administering RXR antagonist HX531 during catagen (Extended Data Fig. 5g,h). In contrast to vehicle control (injected into contralateral back skin), RXR inhibition decreased the number of phagocytic HFSCs and increased the number of unengulfed apoptotic corpses in late catagen. Consistent with their direct RXRα dependency (Fig. 2f), TAM-family and *Mfge8* genes were also sensitive to RXR-inhibition in vivo and did not exhibit upregulation in catagen (Extended Data Fig. 5h). These data bolstered the evidence that RXRα functions integrally in activating the transcriptional phagocytic programme in catagen-phase HFSCs.

## RARγ mediates the catagen RXRα response

**Fig. 3:**
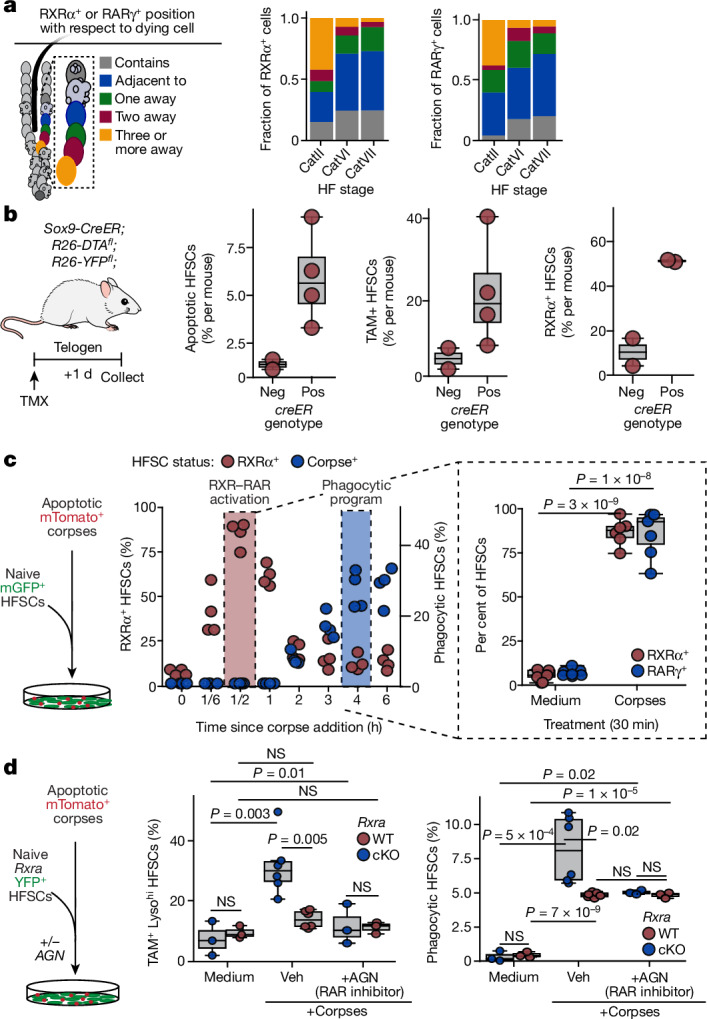
RXRα and RARγ respond to corpse-derived signals to upregulate the phagocytic programme. **a**, Left, schematic of cell position with respect to nearest corpse. Fractions of RXRα^+^ (middle) or RARγ^+^ (right) in position relative to corpses across catagen. *n* = 300 cells across 3 mice per stage. **b**, Left, induction of corpses in quiescent hair follicles. Right, quantification of total FACS-isolated HFSCs that are apoptotic (annexin V^+^), TAM-family^+^ and RXRα^+^ per mouse in control (*Sox9-creER*^*−*^, *n* = 2) or corpse-positive (*Sox9-creER*^*+*^, *n* = 4) mice. **c**, Left, in vitro strategy to expose naive HFSCs to corpses directly. Middle, time-course quantification of total HFSCs that are RXRα^+^ versus containing a corpse. *n* = 4 experiments per time point. Right, quantification of RXRα^+^ and RARγ^+^ HFSCs at 30 min after corpse addition. *n* = 6 (RXRα^+^) and *n* = 7 (RARγ^+^) experiments. **d**, Left, in vitro strategy to expose corpses to naive *Rxra* wild-type or *Rxra*-cKO HFSCs, with or without the pan RAR-family antagonist AGN193109 (AGN). FACS-based quantification of total HFSCs that are both TAM-family^+^ and lysosome^hi^ (middle) or contain engulfed corpses (right) at 4 h after corpse addition. Data represents one (of two) independent experiments. Each dot represents data from one experimental replicate: medium condition (*n* = 3 wild type, *n* = 3 *Rxra*-cKO), for corpses: +Veh (*n* = 6 wild type, *n* = 6 *Rxra*-cKO) and +AGN (*n* = 3 wild type, *n* = 3 *Rxra*-cKO). Pairwise independent two-sided Student’s *t*-tests, *P* values indicated. In box plots, the centre line is the median, box edges delineate first and third quartiles and whiskers extend to 1.5× the inter-quartile range. Further details on statistics and reproducibility in Methods. See also Extended Data Fig. 6 for additional supporting experiments. Source data

**Extended Data Fig. 6:**
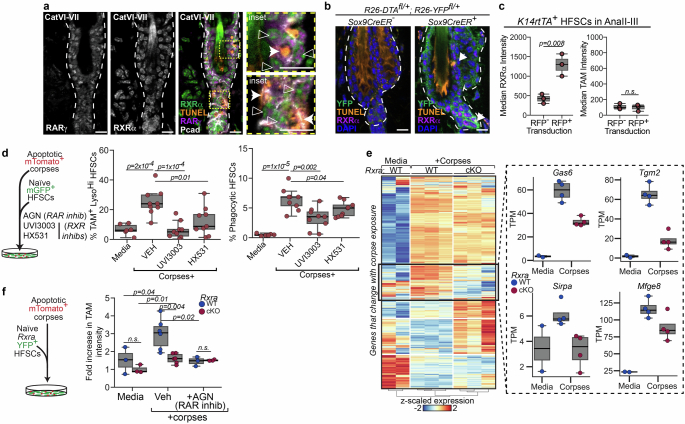
RXRα responds to corpses. **a**, Representative immunofluorescence images for data in Fig. 3a. Nuclear RXRα and RARγ co-occur (open arrowheads) near unengulfed dying cells (closed arrowheads) in mid- late catagen hair follicles (HFs) (white dashed outline). Insets, higher magnification view of yellow boxed regions to left. Scale bar 20 μm. 10-20 HFs per mouse, *n* = 3 animals per hair cycle stage. **b**, Representative immunofluorescence images for FACS-quantified data in Fig. 3b. Diptheria toxin A (DTA) expression in *Sox9CreER*^*+*^ mice creates TUNEL^+^ corpses (white arrowhead) in quiescent HFs (white dashed outline), which induces nuclear RXRα. Scale bar 20 μm. 10-20 HFs analyzed per mouse, *n* = 2 (*Sox9CreER*^*-*^) *n* = 4 (*Sox9CreER*^*=*^) animals. **c**, Quantifications of RXRα intensity (left) and TAM-family expression (right) for FACS-isolated hair follicle stem cells (HFSCs) from *K14rtTA*^+^ mice induced to overexpress RXRα (RFP^+^) at the end of catagen and followed to early anagen (AnaII-III), using the experimental strategy shown in Fig. 2b. *n* = 3 mice. **d**, Left, Experimental schematic. Right, FACS-based quantifications of the percentage of total HFSCs which are TAM-family^+^, lysosome^high^ (middle) or contain corpses (right) 4hrs post corpse addition. Inhibition of RXR-family activity by UVI3003 or HX531. *n* = 6 (Media), *n* = 9 (Corpses+VEH, Corpses+UVI3003, Corpses+HX531) experimental replicates. **e**, Bulk RNA-squencing of *Rxra* wild type (WT) versus *Rxra* conditional knockout (cKO) HFSCs in media or upon corpse exposure. Left, Heatmap of differentially expressed genes (full list in Supplementary Table 4); Z-scaled expression scores of genes (rows; blue, downregulated; red, upregulated) by experimental replicates (columns; *n* = 2 media and *n* = 3 corpse-exposed WT, *n* = 3 corpse-exposed cKO). Right, Expression of phagocytic genes; TPM, transcript per million. *n* = 2 media and *n* = 4 corpse-exposed WT, *n* = 4 corpse-exposed cKO. **f**, FACS-based quantification of TAM-family expression levels 4hrs post corpse addition to RXRα wild type (WT) or conditional knockout (cKO) with or without RAR-family inhibition (+AGN [AGN193109] condition). Quantification represents one of two independent experiments. Each dot represents data from one experimental replicate: Media condition (*n* = 3 WT, *n* = 3 cKO), for corpses: +VEH (*n* = 6 WT, *n* = 6 cKO) and +AGN (*n* = 3 WT, *n* = 3 cKO). Quantifications, pairwise independent Student’s *t*-tests (2-sided), p-values indicated. n.s. not significant (p > 0.05). Data as box-and-whisker plots; box: first-to-third quartiles and median, whiskers 1.5× inter-quartile range. More details on statistics and reproducibility can be found in the Methods section. Source data

Throughout catagen, many RXRα- and RARγ-positive ORS cells were within one cell body distance of an unengulfed corpse (Fig. 3a and Extended Data Fig. 6a). The proximity to corpses appeared to be functionally relevant, as sparsely activated diphtheria toxin subunit A (DTA) in a subset of HFSCs during the resting phase of the hair cycle caused a marked rise in RXRα^+^, TAM-family receptor^+^ expression within the healthy (DTA^−^) HFSCs near dying cells (Fig. 3b and Extended Data Fig. 6b).

In the absence of apoptotic cells, forced expression of RXRα in HFSCs was not sufficient to alter TAM phagocytic receptor expression (Extended Data Fig. 6c), suggesting that factors from apoptotic bodies may also be required to activate this nuclear receptor. Returning to in vitro studies, we showed that within 30 min of corpse addition to naive telogen HFSCs, there was a strong increase in nuclear RXRα and RARγ, and this was followed by apoptotic cell engulfment that plateaued 4–6 h later (Fig. 3c). These events were blocked by transcriptional antagonists against either RAR or RXR families, consistent with the notion that the phagocytic programme depends upon apoptotic corpses and is driven by RARγ–RXRα activity (Extended Data Fig. 6d).

To directly assess the requirement for RXRα in mediating the corpse response, we cultured FACS-isolated YFP^+^ telogen-phase HFSCs from *Rxra* wild-type and *Rxra*-cKO mice and transcriptionally profiled them after corpse addition. Wild-type HFSCs responded by transcriptionally upregulating a cohort of phagocytic genes (full list in Supplementary Table 2). A significant subset of these genes showed a diminished response in *Rxra*-null HFSCs concomitant with functionally impaired apoptotic corpse clearance (Extended Data Fig. 6e). Finally, although affecting apoptotic corpse clearance on its own, the RAR-family inhibitor AGN193109 had no further effect on *Rxra*-null cells, suggesting that the two act cooperatively rather than in parallel (Fig. 3d and Extended Data Fig. 6f). Together, our culture data added compelling evidence that HFSCs directly sense the presence of corpses and respond by upregulating a phagocytic state that requires activated RXRα–RARγ.

## Corpse-secreted signals trigger RXR–RAR

**Extended Data Fig. 7:**
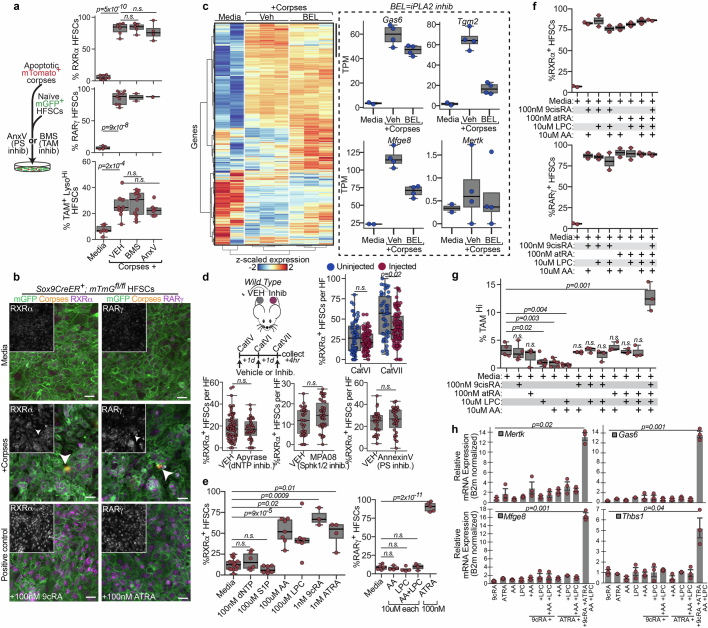
Corpses secrete lysophosphatidylcholine and free fatty acids to activate RXRα and fully upregulate the phagocytic program in vitro. **a**, Quantifications of RXRα^+^ (Top) and RARγ^+^ (Middle) HFSCs 30 min following addition of corpses with (+VEH) or without exposed phosphatidylserine (PS) (+AnxV), or in the presence of TAM-family inhibitor BMS-777607 ( + BMS). Bottom, Percentage of TAM-family^+^;Lysosome^high^ HFSCs 4 hrs after corpse exposure. RXRα^+^ HFSCs: *n* = 7 (Media), *n* = 6 (Corpses+VEH), *n* = 5 (Corpses+BMS), *n* = 4 (Corpses+AnxV); RARγ^+^HFSCs: *n* = 4 (Media), *n* = 9 (Corpses+VEH), *n* = 2 (Corpses+BMS), *n* = 2 (Corpses+AnxV); TAM^+^Lyso^Hi^ HFSCs: *n* = 6 (Media, Corpses+AnxV), *n* = 9 (Corpses+VEH), *n* = 11 (Corpses+BMS) experimental replicates. **b**, Immunofluorescence of HFSCs stained for RXRα or RARγ 30 mins after low-titre corpse addition. Insets are RXRα or RARγ alone. Scale bar, 10 um. Representative of triplicate experiments performed *n* = 3 times. Both addition of retinoic acid (positive controls) and corpses cause increased nuclear fluorescence of RXRα or RARγ (quantified as RXRα^+^ or RARγ^+^, respectively). **c**, Left, Heatmap of bulk RNA-sequencing of wild type HFSC replicates exposed to corpses with (+Veh, vehicle) or without (+BEL, bromoenol lactone) secreted lysophosphatidylcholine (LPC) and fatty acids (FAs). Colour bar, Z-score normalized expression. Right, Transcript per million (TPM) expression values of selected phagocytic program genes. *n* = 2 (Media), *n* = 4 each (Corpses+Veh, Corpses+BEL). Full list in Supplementary Table 5. **d**, Strategy (top left) and percentages of RXRα^+^ HFSCs (bottom) to manipulate corpse-derived secreted nucleotides (left) (*n* = 64 VEH HFs, *n* = 35 Apyrase HFs, 4 mice), sphingosine-1-phosphate (S1P) (middle) (*n* = 32 VEH HFs, *n* = 38 MPA08 HFs, 4 mice), or exposed phosphatidylserine, PS, (right) (*n* = 29 VEH HFs, *n* = 31 AnnexinV HFs, 4 mice) by intradermal injections. Contralateral back skin was injected with vehicle (Veh) control. Top right, Uninjected versus vehicle-injected percentage of RXRα^+^ cells. *n* = 62 HFs uninjected, *n* = 80HFs injected (CatVI) and *n* = 49 HFs uninjected, *n* = 83 HFs injected (CatVII) across 8 mice. **e**, Percentage of RXRα^+^ (left) and RARγ^+^ (right) HFSCs per experiment, 30 min after addition of the indicated recombinant molecules. dATP & dUTP, dNTP; arachidonic acid, AA; 9cRA, 9-*cis* retinoic acid; ATRA, all-*trans* retinoic acid. RXRα^+^ HFSCs: *n* = 14 (Media), *n* = 6 (dNTPs, LPC), *n* = 7 each (S1P, AA), *n* = 3 each (9cRA, ATRA) experimental replicates. RARγ^+^ HFSCs: *n* = 5 (Media, ATRA), *n* = 4 each (AA, LPC), and *n* = 3 (AA + LPC) experimental replicates. **f**, Percentage of HFSCs responding to recombinant molecules by increasing RXRα^+^ cells (top) or RARγ^+^ cells (bottom). *n* = 2 replicates per condition, averaged across technical triplicates. One of two independent experiments shown for each quantification. **g**, Percentage of TAM-family^+^ HFSCs in response to recombinant molecule combinations. One of two independent experiments shown. *n* = 4 (Media, 9cRA, LPC + AA), *n* = 6 (LPC, AA), *n* = 3 (remaining conditions) experimental replicates. **h**, Quantitative RT-PCR for phagocytic gene transcripts, relative to B2-microglobulin (*B2m*) levels, and normalized to media-only conditions. Concentrations of indicated added molecules are as in (g). *n* = 3 experimental replicates, performed in technical triplicates. Data presented as mean ± s.e.m (error bars). Quantifications, pairwise independent Student’s *t*-tests (2-sided), p-values indicated. n.s. not significant (p > 0.05). Data as box-and-whisker plots; box: first-to-third quartiles and median, whiskers 1.5× inter-quartile range. More details on statistics and reproducibility can be found in the Methods section. Source data

Our results were notable given that the phagocytic programme in macrophages is also influenced by RXRs^16–19^, but the heterodimeric partners involved differ. Moreover, in macrophages, elevation of TAM-family receptors via RXR–PPAR/LXR signalling requires engulfment and digestion of corpses^16–19^, whereas HFSCs required neither engulfment nor corpse digestion for nuclear RXRα–RARγ and its downstream activation of TAM-family and lysosomal genes (Extended Data Fig. 7a). Thus, despite certain parallels, the mechanism of activating RXR signalling between non-professional and professional phagocytes appeared to be distinct. Our findings raised the possibility that the distinctions reside not only in RXR co-receptors, but also their ligands.

**Fig. 4:**
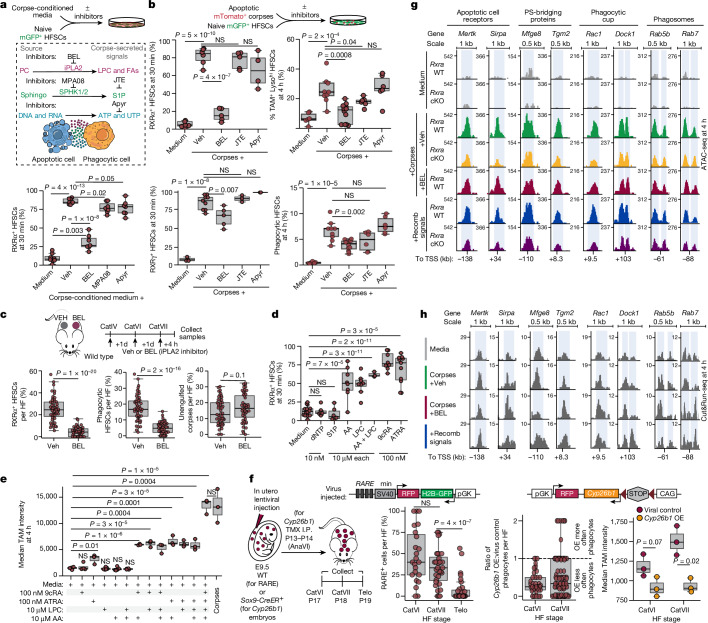
Corpse-derived LPC and fatty acids work with HFSC-derived RA to regulate phagocytosis. **a**, Top, experiment interrogating response to corpse-secreted factors: BEL, calcium-independent phospholipase A2 inhibitor; PC, phosphatidylcholine; FAs, fatty acids; JTE, JTE013 (S1P-receptor antagonist); sphingo, sphingosine; Apyr, Apyrase (ATP hydrolysis). Bottom, quantification of HFSCs with nuclear RXRα^+^. *n* = 8 (medium), *n* = 7 (rest) experiments per condition. **b**, Top, experimental design interrogating response to corpses. Bottom, quantified data. RXRα^+^ HFSCs: *n* = 7 (medium), *n* = 6 (corpses plus Veh), *n* = 5 (corpses plus BEL, corpses plus JTE), *n* = 4 (corpses plus Apyr) experiments. RARγ^+^ HFSCs: *n* = 4 (medium), *n* = 9 (corpses plus Veh), *n* = 5 (corpses plus BEL), *n* = 3 (corpses plus JTE, corpses plus Apyr) experiments. TAM-family^+^;lysosome^high^ (middle) and corpse-containing (bottom) HFSCs. *n* = 9 (corpses plus Veh), *n* = 12 (corpses plus BEL), *n* = 6 (medium, corpses plus JTE, corpses plus Apyr) experiments. **c**, Top, strategy to block LPC and fatty acids. Bottom, quantified data. RXRα^+^ (left), phagocytic (middle) HFSCs, unengulfed corpses (right). *n* = 51 hair follicles (Veh), *n* = 48 hair follicles (BEL) across 6 mice. **d**, RXRα^+^ response to recombinant factors. dNTP, 10 nM dUTP + dATP. *n* = 15 (medium), *n* = 6 (dNTP, S1P), *n* = 8 (arachidonic acid (AA), LPC, 9cRA), *n* = 3 (AA plus LPC), *n* = 11 (ATRA) experiments. **e**, AA, LPC and RA stimulate expression of TAM-family proteins. *n* = 4 (medium, 9cRA, LPC plus AA), *n* = 6 (LPC, AA), *n* = 3 (remaining conditions) experimental replicates. One representative experiment (of two) shown. **f**, RA is required for phagocytosis in vivo. Left, experimental design. Middle, hair cycle-dependent RXR–RAR-reporter activity. *n* = 23 (CatVI), *n* = 32 each (CatVII,Telo) hair follicles (2 mice per stage). Right, phagocytic sensitivity to RA degradation. Quantification of phagocytic HFSCs (*n* = 3 mice), TAM-family intensity per mouse (*n* = 6 mice). OE, overexpression. **g**,**h**, ATAC-seq (**g**) and RXRα CUT&RUN-seq (**h**) peak tracks (replicate-pooled) for phagocytic gene enhancers from *Rxra* wild-type or *Rxra*-cKO HFSCs treated as indicated. Recombinant (recomb) signals: RA + LPC + AA. Differentially accessible peaks are shown in light blue. No RXRα enrichment was detected in *Rxra*-cKO HFSCs. Pairwise independent two-sided Student’s *t-*tests, *P* values indicated. NS, not significant (*P* > 0.1). In box plots, the centre line is the median, box edges delineate first and third quartiles and whiskers extend to 1.5× the inter-quartile range. Further details on statistics and reproducibility in Methods. See also Extended Data Figs. 7–9 for additional supporting experiments. Source data

Of note, when even one apoptotic corpse was added per 100 healthy HFSCs in vitro, local increases were seen in RXRα–RARγ-positive cells around each corpse (Extended Data Fig. 7b), reminiscent of that seen throughout catagen in vivo. Corpse-conditioned medium achieved a similar response, indicating that factors secreted by corpses are involved (Fig. 4a,b). The best characterized ‘find-me’ signals secreted by apoptotic cells are free nucleotides, sphingosine-1-phosphate (S1P) and lysophosphatidylcholine^20–22^ (LPC). Using small molecule inhibitors to block the generation of either S1P (by inhibiting SPHK1 and SPHK2 (SPHK1/2) with MPA08), LPC (by inhibiting calcium-independent phospholipase A2 (iPLA2)-mediated phosphatidylcholine cleavage with bromoenol lactone (BEL)) or free nucleotides (by inducing their degradation with recombinant apyrase (Apyr)), we found that LPC promoted RXRα–RARγ activation. Correspondingly, when LPC generation was blocked in apoptotic corpses, healthy HFSCs failed to upregulate TAM and lysosomal genes needed for corpse engulfment (Fig. 4b). RNA sequencing verified that the effects of impairing phosphatidylcholine cleavage were at the transcriptional level, with moderate downregulation in a subset of apoptotic cell clearance receptors, phosphatidylserine-bridging proteins, phagocytic cup formation, and mediators of phagolysosome maturation (Extended Data Fig. 7c).

Eliminating LPC production during catagen in vivo significantly diminished both RXRα upregulation in hair follicle stem and progenitor cells adjacent to corpses, and numbers of corpse-containing ORS cells (Fig. 4c). Consistent with our in vitro findings, blocking the generation of S1P, free nucleotides or exposure of phosphatidylserine in vivo did not appreciably affect RXRα activation or the phagocytic programme (Extended Data Fig. 7d). These data pointed to the view that the cleavage of phosphatidylcholine to generate LPC and free fatty acids^20,23^ in apoptotic cells acts locally to activate RXRα signalling and induce a phagocytic state in their healthy neighbours.

Activated by caspase 3, iPLA2 hydrolyses phosphatidylcholines at the sn-2 position to generate LPC and free fatty acids^20,23,24^. A major constituent at the sn-2 position is the fatty acid arachidonic acid (AA), which has been described as a natural ligand for RXR^25^. Indeed, recombinant AA and/or LPC boosted nuclear RXRα intensity in roughly 50% of cultured HFSCs across a physiologically relevant range of concentrations^20,25^. By contrast, RARγ did not respond to AA or LPC, consistent with its classic ligands being 9-*cis* retinoic acid^26,27^ (9cRA) and all-*trans* retinoic acid^28,29^ (ATRA) (Fig. 4d and Extended Data Fig. 7e). Consistently, the combination of retinoic acid (RA) and LPC (and/or AA) yielded the highest levels of nuclear RXRα and RARγ (Extended Data Fig. 7f). Additionally, TAM receptor expression comparable to corpse-exposure could be achieved simply by exposure to AA, LPC and RA (Fig. 4e and Extended Data Fig. 7g,h).

**Extended Data Fig. 8:**
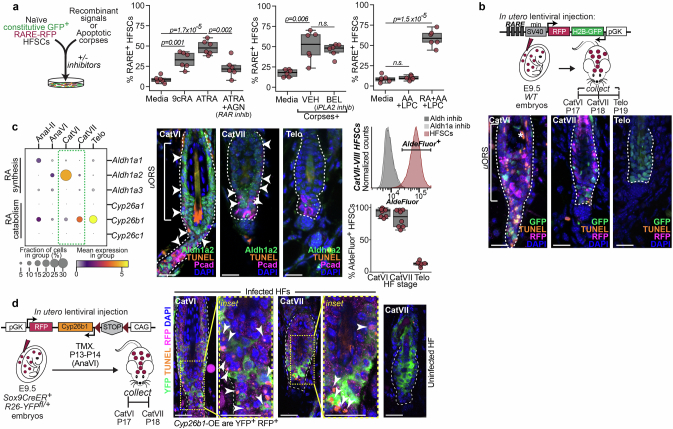
Catagen HFSCs produce and respond to retinoic acid to upregulate the phagocytic program. **a**, Left, Schematic to expose Retinoic Acid Response Element (RARE)-driven RFP; constitutive H2B-GFP expressing HFSCs to signals in vitro. Middle left, Quantification of RARE^+^ HFSCs to RA isoforms 9-*cis* retinoic acid (9cRA) or all-*trans* retinoic acid (ATRA) with or without the RAR-inhibitor (+AGN; AGN193109). Middle right, Quantifications of RARE+ HFSCs exposed to corpses with or without lysophosphatidylcholine (LPC)/free fatty acids (+BEL; bromoenol lactone) (left) or to recombinant arachidonic acid (AA) and LPC with or without retinoic acid (RA) (right). *n* = 6 experimental replicates per condition. **b**, Top, Strategy to express RARE-driven RFP with constitutive H2B-GFP reporter in skin. Bottom, Sagittal immunofluorescence of RARE-infected HFSCs across the hair cycle, asterisk denotes hair shaft keratinization; quantified in Fig. 4f. Images and quantification are representative of 10-15 HFs per mouse, *n* = 2 mice per hair cycle stage. Scale bar, 20 μm. **c**, Left, Dot plot summarizing retinoic acid synthesis and catabolism enzyme expression across the hair cycle from single cell transcriptomic data. Middle, Sagittal wild type skin sections from mid-late catagen and early telogen stages of the hair cycle stained for retinoic acid rate-limiting enzyme Aldh1a2 (arrowheads). Dashed line, dermo-epithelial border. Scale bar, 20 μm. Right, FACS plots showing normalized counts of Aldh1a activity on the AldeFluor substrate in late catagen HFSCs (top) quantified across the hair cycle (bottom). *n* = 6 mice per hair cycle stage. **d**, Left, Experimental strategy to deplete RA from HFs in vivo. TMX, Tamoxifen. Right, Sagittal imunofluorescence of transduced (left and middle) versus untransduced catagen HFs. Boxed regions are magnified at right. Cyp26b1 over-expressing (OE) cells are YFP^+^RFP^+^ while virus control are RFP^+^. Scale bar, 20 um. Data are quantified in Fig. 4f, n = 3 mice per hair cycle stage. Quantifications, pairwise independent Student’s *t*-tests (2-sided), p-values indicated. n.s. not significant (p > 0.05). Data as box-and-whisker plots; box: first-to-third quartiles and median, whiskers 1.5× inter-quartile range. More details on statistics and reproducibility can be found in the Methods section. Source data

If RXRα–RARγ signalling is key, it should occur in the uORS during late catagen. To test for this activity, we used a lentivirus with RAR–RXR response elements (RARE) to drive RFP, as well as a constitutively expressed GFP. We first showed that in vitro, RFP expression was upregulated upon exposure to each RA isoform and abrogated by RAR inhibitor AGN193109. Exposure to apoptotic corpses, with or without LPC and free fatty acids, also induced marked reporter activity (Extended Data Fig. 8a). Using in utero lentiviral delivery, we then transduced the skin epithelium and examined RARE-RFP activity during the hair cycle of adult mice. As shown in Fig. 4f and Extended Data Fig. 8b, reporter activity was strongest in the HFSCs during catagen and was dampened by telogen. Moreover, retinaldehyde dehydrogenase 2 (ALDH1A2), which is required for RA synthesis^30^, was expressed and active in the ORS specifically during catagen (Extended Data Fig. 8c). Consistently, when we employed a similar lentiviral approach to inducibly increase expression of CYP26B1, which promotes RA degradation^31^, uORS cells were less phagocytic than their control counterparts under mosaic conditions, and did not increase expression of TAM genes (Fig. 4f and Extended Data Fig. 8d). Together, these data suggest that signals from dying corpses (AA and LPC) and those generated by their healthy neighbours (RA) converge to activate RARγ–RXRα and trigger the phagocytic programme in the catagen ORS. The data further imply that once corpses are cleared, the nuclear receptor naturally shuts off, allowing remaining stem cells to return to quiescence for future hair cycles.

## Regulatory mechanisms of phagocytosis

**Extended Data Fig. 9:**
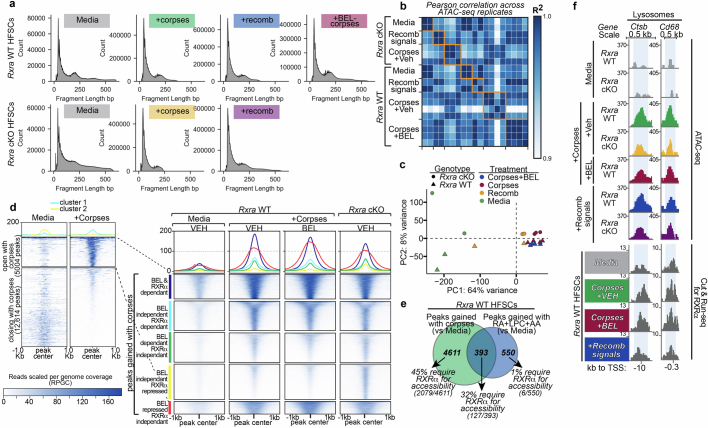
ATAC-seq shows differential chromatin accessibility in response to corpse-derived lysophosphatidylcholine and arachidonic acid dependent on RXRα. **a**, Representative histograms of fragment lengths in base pairs (bp) detected for cultured *Rxra* HFSCs ATAC-seq replicates. recomb, 9-*cis* retinoic acid +all-*trans* retinoic acid +lysophosphatidylcholine +arachidonic acid; WT, wild type; cKO, conditional knockout. **b**, Pearson correlation (R^2^) values for reads mapped to peaks on cultured *Rxra* HFSCs ATAC-seq replicates. *n* = 2 each (Media, Recomb) and *n* = 4 each (Corpses+Veh, Corpses+BEL) biological replicates per genotype. **c**, Principal component analysis on reads mapped to peaks. Biological replicates as in (b). **d**, Heatmap showing all peaks with significantly altered accessibility between *Rxra* WT and cKO pooled replicates exposed to corpses with (+Veh, vehicle) or without (+BEL, bromoenol lactone) lysophosphatidylcholine + arachidonic acid. Signal for pooled replicate samples is in reads per genome coverage (RPGC) (colour bar legend, right). Each row corresponds to a detected peak, centered and extended +/− 1 kb. Peaks are clustered based on differential accessibility relative to *Rxra* WT+Veh corpses, and summarized as the mean accessibility signal per region in a dark blue line (peaks that close in BEL and *Rxra* cKO), a light blue line (peaks that close in cKO only), a green line (peaks that close in BEL only), a yellow line (peaks that open in cKO only) or a red line (peaks that open in BEL only) in the graph at top. **e**, Venn diagram representation comparing peaks that open in response to corpses with those gained in response to retinoic acid (RA) + lysophosphatidylcholine (LPC) + arachidonic acid (AA) (‘Recomb’ condition) in *Rxra* WT HFSCs. Arrows indicate the percentage of peaks that require RXRα for accessibility within each category. Notably, enhancer peaks associated to phagocytic genes fall within the 32% of peaks gained in response to both corpses and recombinant signal that require *Rxra* for accessibility, which are shown in Fig. 4g. **f**, Top, ATAC-seq replicate-pooled peak tracks for lysosomal gene enhancers in cultured *Rxra* WT and cKO HFSCs exposed to apoptotic corpses with (+Veh) or without (+BEL) secreted LPC and FAs, or treated with RA + LPC + AA (+Recomb signals) for 4 hrs. Peaks with differential accessibility highlighted in light blue. Bottom, Cut & Run-seq for RXRα replicate pooled peak tracks for same enhancers above, in cultured *Rxra* WT HFSCs exposed to apoptotic corpses with (+Veh) or without (+BEL) secreted LPC and FAs, or treated with RA + LPC + AA (+Recomb signals) for 4 hrs. Peak tracks in reads per genome coverage (RPGC). No peak enrichment was observed in *Rxra* cKO HFSCs at these loci.

To further characterize the dependency of phagocytic programme genes on active RXRα–RARγ, we used ATAC-seq and profiled wild-type and *Rxra*-cKO HFSCs in three complementary in vitro settings: (1) in the absence of corpses or signalling cocktail (‘Medium’); (2) in response to corpses with (+Veh) or without (+BEL) phosphatidylcholine hydrolysis and generation of LPC and AA; and (3) in response to AA, LPC and RA combined (Fig. 4g and Extended Data Fig. 9a–e). In medium alone, phagocytic genes were in a closed chromatin state. Upon corpse exposure, more than 12,000 peaks lost accessibility and around 5,000 peaks gained accessibility. Approximately half the peaks that gained accessibility were diminished upon *Rxra* ablation. These peaks were also sensitive to the presence of LPC and fatty acids as documented by their decline upon exposure to BEL-treated corpses. Many of these peaks resided within putative enhancers for genes involved in multiple stages of apoptotic cell clearance in vivo as well as in vitro. Notably, a cocktail of AA, LPC and RA recapitulated the effect of corpses on approximately one-third of RXRα-dependent peaks, including those in enhancers of genes encoding apoptotic cell recognition, engulfment and processing pathways.

**Fig. 5:**
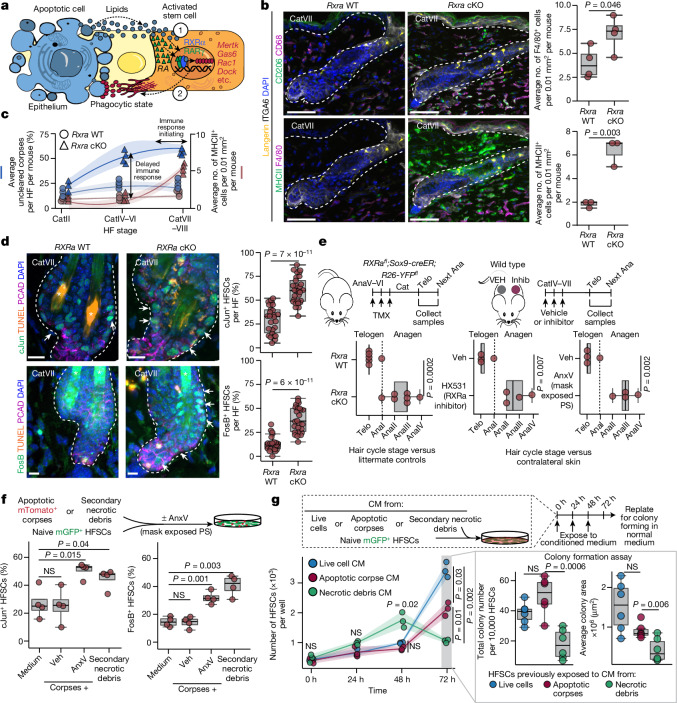
Stem cell-mediated corpse clearance protects the niche for tissue homeostasis. **a**, Model for HFSC sensing of corpses. **b**, Left, multiplexed immunofluorescence of *Rxra* wild-type versus catagen-specific *Rxra*-cKO hair follicles (strategy as in Fig. 2d). Cell-type markers: CD68, monocytic phagocytes; CD206, mature macrophages; langerin, epidermal-resident macrophages; ITGA6, epidermal progenitors; F4/80, pan-macrophage; MHCII, pan-immune cells. Right, quantification (top; F4/80, *n* = 4 mice per genotype); (bottom; MHCII, *n* = 3 mice per genotype) per dermal area. Scale bars, 50 μm. **c**, Percentage of unengulfed corpses per hair follicle and immune response (MHCII^+^ cells per dermal area). *n* = 3 (CatII) and *n* = 4 (CatVI, CatVII) mice per genotype. Shaded area represents 95% confidence interval. **d**, Immunofluorescence showing stress factors (cJun and FosB) and DNA damage (TUNEL). P-cadherin (PCAD) is a hair germ marker. Asterisk highlights hair shaft autofluorescence. cJun: *n* = 26 hair follicles (4 wild-type mice), *n* = 29 hair follicles (3 *Rxra*-cKO mice); FosB: *n* = 26 hair follicles per genotype (3 mice per genotype). Scale bars, 10 μm. **e**, Left, strategy to ablate *Rxra* at catagen entry and analyse subsequent telogen and anagen periods (top) and quantification (bottom) of hair cycle stage. *n* = 6 wild-type and *n* = 7 cKO mice. Right, strategy (top) to inhibit corpse clearance by HX531 (RXR inhibitor, left) or annexin V (to mask phosphatidylserine, right) versus contralateral vehicle in catagen, and quantification (bottom) on subsequent hair cycle stage. *n* = 4 mice per experimental condition. Inhib, inhibitor. **f**, Top, schematic of experiment to expose HFSCs to necrotic debris by blocking engulfment with annexin V or using necrotic-conditioned medium. Bottom, quantification of stress factors after exposure. *n* = 4 experiments per condition (averaged duplicates). **g**, Top, experimental design to repeatedly expose naive HFSCs to necrotic debris. Bottom left, quantification of HFSCs over time (one of three independent experiments shown). *n* = 3 replicates per condition per time point. Shaded area represents 95% confidence interval. Bottom right, colony-forming efficiency (middle) and size (right) upon passage of HFSCs exposed to repeated damage. *n* = 6 experiments per condition. CM, conditioned medium. Quantifications, multiple pairwise independent two-sided Student’s *t*-tests, *P* values indicated. NS, not significant (*P* > 0.05). In box plots, the centre line is the median, box edges delineate first and third quartiles and whiskers extend to 1.5× the inter-quartile range. Further details on statistics and reproducibility in Methods. See also Extended Data Fig. 10 for additional supporting experiments. Source data

We corroborated direct regulation of these genes by performing cleavage-under-targets-and-release-using-nuclease (CUT&RUN) sequencing with an antibody against RXRα. As shown in Fig. 4h and Extended Data Fig. 9f, binding was detected at phagocytic programme enhancers that were opened upon corpse exposure and sensitive to the presence of LPC and fatty acids, as well as being recapitulated by the cocktail of recombinant signals. Together our data point to a mechanism whereby HFSCs can sense apoptotic corpse signals in combination with local tissue RA signals to induce nuclear RXRα–RARγ signalling and orchestrate a transient phagocytic state (Fig. 5a).

## Phagocytosis and HFSC niche homeostasis

In many tissue contexts where non-professional phagocytosis is deployed, macrophages or dendritic cells compensate for impaired epithelial apoptotic cell clearance^32^. However, HFSCs are thought to reside in immune privileged niches, leading us to explore whether and how skin-resident professional phagocytes might compensate for impaired apoptotic cell clearance.

**Extended Data Fig. 10:**
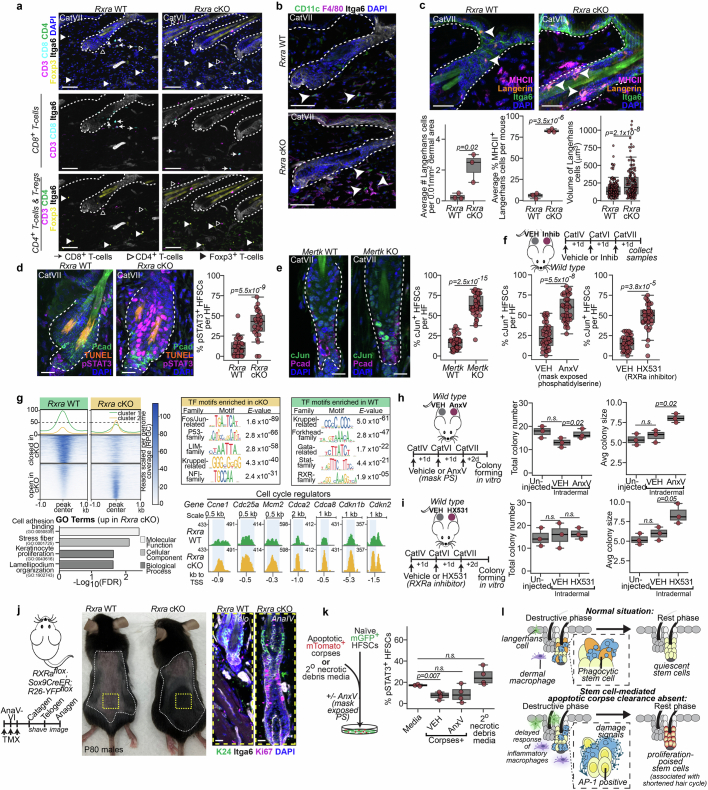
HFSC-mediated apoptotic corpse clearance is necessary to preserve tissue homeostasis. **a**, Multiplexed iterative immunofluorescence for T-cell markers on *Rxra* wild type (WT) and conditional knockout (cKO) skin. Itga6, integrin-α6. Scale bar, 50 um. Images representative of 15 HFs/mouse, 2 mice/genotype. **b**, Multiplexed iterative immunofluorescence for CD11c^+^ dendritic cells (arrowheads) and F4/80^+^ macrophages on *Rxra* WT and cKO skin. Images representative of 15 HFs/mouse, 2 mice/genotype. Additional characterization/quantification of macrophage phenotype in Fig. 5a. Scale bar, 30 um. **c**, Top, Sagittal immunofluorescence of Langerhans cells in the upper HF (above the sebaceous gland) in *Rxra* WT and cKO skin. Scale bar, 10 um. Bottom, Quantifications of Langerhans cells (LCs) in the dermis (left), MHCII-high Langerhans cells in the skin (middle), and Langerhans cell volume (right) in *Rxra* WT and cKO skin (*n* = 213 [WT] and 146 [cKO] Langerhans cells). *n* = 3 mice per genotype. **d**, Left, Sagittal immunofluorescence for phospho-STAT3 (pSTAT3) and quantifications (right) in *Rxra* WT and cKO HFs. Scale bar, 10 um. *n* = 27 HFs across 3 mice per genotype. **e**, Left, Sagittal immunofluorescence and quantifications (right) for cJun^+^ HFSCs in *Mertk* WT and KO HFs. Scale bar, 15 um. *n* = 39 HFs (WT) and 40 HFs (KO) across 3 mice per genotype. **f**, Top, Schematic to block apoptotic corpse engulfment by intradermal injection of small molecule inhibitors (Inhib) during catagen followed by quantifications of cJun^+^ HFSCs (bottom) in annexinV (AnxV)-treated skin (to mask exposed phosphatidylserine, left) or HX531 treated skin (to inhibit RXRα, right). AnnexinV experiment: *n* = 37 HFs (VEH) and *n* = 38 HFs (AnxV) across 3 mice. HX531 experiment: *n* = 34 HFs per treatment across 3 mice. **g**, Top left, Heatmap of differentially accessible peaks in late catagen HFSCs between *Rxra* WT and cKO pooled replicates, annotated as in Extended Data Fig. 9d. Top middle and right, Motif enrichment analysis of the peaks gaining (middle) or decreasing (right) accessibility in *Rxra* cKO. Bottom left, Bar chart of gene ontology (GO) terms of genes associated to peaks gained in cKO, ordered on false discorvery rate (FDR). Bottom right, ATAC-seq replicate-pooled peak tracks for cell cycle gene promoters in FACS-isolated *Rxra* WT and cKO CatVII HFSCs, scaled, normalized, and annotated as in Fig. 2f. Peaks with differential accessibility highlighted in light blue. **h,i**, Left, Experimental design to block corpse engulfment by masking exposed phosphatidylserine (PS) on apoptotic cells with annexinV (AnxV) (**h**) or by inhibiting RXRα with HX531 (**i**) in catagen HFSCs versus respective contralateral vehicle (VEH) controls in vivo, followed by HFSC isolation and colony forming assays in vitro. Quantifications of total colony number (middle) and average colony size (right) following 7 days of culture. *n* = 3 mice per condition averaged across technical triplicates. **j**, Left, Schematic to conditionally ablate *Rxra* immediately prior to catagen, and examine the consequences in the subsequent hair cycle. Middle, Representative image of whole back skin of *Rxra* WT and cKO littermates, with shaved area denoted by dashed line. Right, Saggital immunofluorescence of age-matched skin from *Rxra* WT and cKO littermates for hair cycle staging. *n* = 6 WT and *n* = 7 cKO mice. Data quantified in Fig. 5e. **k**, Experimental design (left) and quantification (right) of pSTAT3^+^ HFSCs exposed to corpses they can (VEH) or can’t (AnxV, annexinV) engulf or secondarily necrotic debris. *n* = 3 (Media) or *n* = 4 each (other conditions) experiments/condition (averaged duplicates). **l**, Model for how stem cell-mediated apoptotic cell clearance protects the niche for tissue homeostasis. Quantifications, pairwise independent Student’s *t*-tests (2-sided), p-values indicated. n.s. not significant (p > 0.05). Data as box-and-whisker plots; box: first-to-third quartiles and median, whiskers 1.5× inter-quartile range. More details on statistics and reproducibility can be found in the Methods section. Source data

To test this possibility, we conditionally ablated *Rxra* in the hair follicle ORS as before and monitored the skin through catagen, this time performing comprehensive profiling of the tissue resident immune cells using iterative multiplexed immunofluorescence analysis (Fig. 5b and Extended Data Fig. 10a–c). At the end of catagen, overall numbers of T cells, dendritic cells and Langerhans cells were unchanged, although Langerhans cells had entered the dermis of *Rxra*-cKO skin. Additionally, dermal macrophages were increased approximately twofold relative to in wild-type control skin. Both dermal macrophages and Langerhans cells showed signs of activation, with upregulation of the phagocytic receptor CD206 on dermal macrophages, increased branching of dendritic spines on Langerhans cells and increased major histocompatibility class II (MHCII) on both.

Despite these professional immune phagocytes sensing and responding to the undigested corpses, the kinetics of immune influx were protracted and signs were evident of secondary necrosis and release of pro-inflammatory damage-associated molecular patterns in *Rxra*-null hair follicles (Figs. 2d and 5b,c). Indeed, through either *Rxra* or *Mertk* knockout or transient inhibition of phagocytosis with small molecules, phagocyte-deficient catagen HFSCs displayed nuclear phospho-STAT3 and AP-1 transcription factors—hallmarks of a tissue damage response^33,34^ (Fig. 5d and Extended Data Fig. 10d–f). Moreover, profiling of accessible chromatin of late catagen *Rxra*-null HFSCs revealed regions associated with cell adhesion, cytoskeleton and proliferation genes that were significantly enriched for AP-1 transcription factor motifs (Extended Data Fig. 10g). Consistent with the expected response to tissue damage and increased accessibility of cell cycle promoters, HFSCs exposed to uncleared apoptotic corpses in vivo formed colonies in vitro with comparable efficiency, but with higher rates of proliferation, and therefore increased colony size (Extended Data Fig. 10h,i). In the natural hair cycle, this pro-proliferative HFSC state shortened the resting phase of the hair cycle (Fig. 5e and Extended Data Fig. 10j).

To assess whether the link between uncleared corpses and precocious entry into the hair cycle was directly attributable to an ability of HFSCs to sense damage-associated molecular patterns autonomously, we first exposed HFSCs in vitro to either necrotic-conditioned medium or apoptotic corpses that they could not engulf. In stark contrast to control medium or corpses that they could clear, HFSCs responded to necrotic debris by upregulating AP-1 transcription factors within 1 h (Fig. 5f and Extended Data Fig. 10k). Moreover, although HFSCs displayed an initial burst of proliferation in response to necrotic debris, their colony forming efficiency and proliferation waned over time in comparison to both control and apoptotic corpse-conditioned medium (Fig. 5g). These findings are consistent with the view that when stem cells are exposed to necrotic debris from corpses that they cannot engulf, they activate a damage response, transiently stimulating proliferation, but with an ultimate cost to fitness (Extended Data Fig. 10l).

## Discussion

Death is a fundamental aspect of life, not only in organisms but also in tissues. As dedicated professional phagocytes, macrophages and dendritic cells can migrate into injured tissues and seek out dying cells^1–3^. When confronted with death during homeostasis, however, tissues often call upon non-professional phagocytes to perform these duties^2–4^. The destructive phase of the hair cycle proved to be an excellent model to unravel some of the molecular mechanisms involved.

From the elegant studies of Mesa et al.^10^, it was known that at the start of the destructive phase, dermal papilla cells transmit a TGFβ signal that causes hair progenitors to terminally differentiate and lower ORS cells to apoptose^9,10^. As the hair follicle regresses, the dermal papilla is drawn upward, exposing the HFSCs in the upper ORS to this apoptotic signal for the first time. By studying the process in late catagen—that is, before telogen silences this death signal^12^—we learned that a considerable percentage of HFSCs dies, while each HFSC that survives contains multiple corpses. By engulfing corpses, HFSCs appear to gain a competitive advantage, since without the ability to phagocytose corpses, the quiescence controls that are essential for the long-term maintenance of the stem cell pool were disrupted (Extended Data Fig. 10l). Moreover, as our studies revealed, the link between HFSC efferocytosis and maintaining the quiescent state is an autonomous one as it could be recapitulated in vitro.

LPC and AA are known to attract phagocytic macrophages^20^, but in studying phagocytosis in HFSCs, we revealed an additional role for RA. The roots of these multifaceted requirements appear to reside in nuclear RXRα–RARγ signalling, as RXRα has been reported to bind fatty acids, and RA is essential for activating RARγ^27,35,36^. By requiring some signals emanating from healthy HFSCs and others from dying cells, phagocytosis is exquisitely tuned, triggered at the start of catagen when apoptotic ORS cells first appear, but then rapidly curtailed by the end of catagen, when healthy ORS cells become limiting. This ensures that at the end of the destructive phase, some HFSCs are not only retained for the next hair cycle, but can also return to their primary function of maintaining and regenerating tissue.

We speculate that this method of controlling apoptotic corpse clearance may function in other non-professional phagocytes, which must cope with sporadic cell death while maintaining their normal tissue tasks. Indeed, many tissues utilize the same phagocytic pathways for detecting and engulfing apoptotic corpses^4,37,38^. Additionally, as we show, putative enhancers for many of these genes contain RXRα sequence motifs and directly bind RXRα, further underscoring the orchestrated path to activating the phagocytic programme and eliminating dying cells at the right time and place.

Another notable facet of having the pathway dependent upon RXRs is that whereas corpse-dependent production of lyso-lipids and free fatty acids can act as the universal activator of this transcription factor, RXRs can heterodimerize with diverse binding partners each of which have their own set of ligands, which are differentially produced across tissue conditions. By having a combinatorial trigger dependent upon both healthy cells and apoptotic neighbours, phagocytosis can be spatially and temporally tailored to suit the needs of each tissue while balancing the system to maintain fitness.

Finally, despite a response by professional phagocytes to uncleared ORS corpses, their delayed kinetics was insufficient to prevent tissue damage when the pathway was crippled in the HFSC niche. In other scenarios in which the threshold for HFSC activation is reduced and/or HFSC usage is accelerated, as it was here, HFSCs keep up in the short term, but the pool is eventually exhausted, leading to premature aging or balding^39,40^. Although possible secondary effects precluded long-term studies here, it is tempting to speculate that accelerated stem cell usage caused by cyclical bouts of dysregulated efferocytosis may similarly take a toll on preserving the stem cell pool. Consistent with this is our finding that stem cells that cannot engulf leave behind necrotic debris that briefly stimulates HFSCs in vitro but then quickly leads to their exhaustion. Such features may also come into play in other stem cell niches such as the brain subventricular zone, where a subpopulation of neuronal progenitors clear their dying neighbours in the face of continual, rather than episodic, niche cell death^6^. In closing, the contributions of stem cells to apoptotic cell clearance provides a powerful mechanism for rapidly clearing dying cells and preventing tissue damage, while enabling residence in immune-privileged homeostatic niches.

## Methods

The following previously generated mouse lines were used in this study: *Rxra*^*fl*^ (ref. ^41^; Jax stock 013086), *Sox9-creER* (ref. ^42^), *Krt14-rtTA* (ref. ^43^; Jax stock 008099), *Rosa26*^*lox-STOP-lox-YFP*^ (ref. ^44^; Jax stock 006148; referred to as *R26*^*YFP*^), *Rosa26*^*mTmG*^ (ref. ^45^; Jax stock 007576; referred to as *R26*^*mTmG*^), *Rosa26*^*Brainbow2.1*^ (ref. ^46^; Jax stock 013731, referred to as *R26*^*Brainbow2.1*^), *Rosa26*^*lox-STOP-lox-Cas9-EGFP*^ (ref. ^47^; Jax stock 026175, referred to as *R26*^*Cas9-EGFP*^), *Rosa26*^*lox-STOP-lox-DTA*^ (ref. ^48^; Jax stock 010527, referred to as *R26*^*DTA*^) and *Mertk*^*−/−*^ (full knockout; ref. ^49^). The *Mertk*-knockout mice used in this study are referred to as *Mertk*^*−/−V2*^ in the originating paper. Wild-type CD1 or C57BL/6 mice were originally purchased from Charles River and The Jackson Laboratories, respectively, and maintained as in house colonies.

Mice were maintained and bred under specific-pathogen-free conditions at the Comparative Bioscience Center (CBC) at The Rockefeller University, an Association for Assessment and Accreditation of Laboratory Animal Care (AALAC)-accredited facility. *Mertk*-knockout mice and C57BL/6J wild-type controls (maintained as separate colonies) were bred and maintained in a specific-pathogen-free facility at Yale University. All mice were bred and maintained under a strict 12-h light cycle and fed with standard chow. The temperature of the animal rooms was 20–26 °C, and the humidity was 30–70%. Adult mice were housed in cage with a maximum of five mice. All mouse protocols were approved by the Institutional Animal Care and Use Committee (IACUC) at The Rockefeller University, or by the IACUC at Yale University.

For comparative assessments of phenotype between control and mutant mice, age and sex matched mice were used, with preference given to littermate controls wherever possible, and sample size greater than three mice per genotype or condition across multiple litters whenever possible. For our inducible overexpression studies, a *Krt14-rtTA*^+/−^ (heterozygous) male was mated with CD1 females and all offspring were transduced with lentivirus at E9.5 (see following sections). Offspring of both genotypes received doxycycline by intraperitoneal injection (0.5 mg per mouse) at P14 to activate *Krt14-rtTA* within 12 h, and expression was maintained by feeding the mother and pups doxycycline (2 mg kg^−1^) chow (Bioserv). *Krt14-rtTA*^−^ mice were used as control, with *Krt14-rtTA*^+^ mice as the experimental group. To generate *Rxra* control and cKO mice for experiments, the *Rxra*^*fl*^ line was crossed with *Sox9-creER*^*+*^*; R26*^*YFP*^ mice*. Sox9-creER*^−^ mice with any *Rxra*^*fl*^*;R26*^*YFP*^genotype, and *Rxra*^*fl/+*^*;Sox9-creER*^*+*^*;R26-YFP*^*fl/+*^mice were used as controls, while experimental mice were *Rxra*^*fl/fl*^*;Sox9-creER*^*+*^*;R26-YFP*^*fl/+*^. All mice received tamoxifen (2% in corn oil) (Sigma-Aldrich) to activate *Sox9-creER*, administered by intraperitoneal injection once a day for 3 days, as indicated. *Sox9-creER* was similarly activated when crossed to *R26*^*mTmG*^ (to label HFSCs prior to FACS-isolation and culture), *R26*^*Brainbow2.1*^ (to stochastically label HFSCs and identify functional phagocytes), *R26*^*Cas9-EGFP*^ (to mosaically knockout *Rxra*), and upon transduction with the inducible Cyp26b1 expression construct (to degrade RA in HFSCs). To activate *Sox9-creER* sparsely when crossed to *R26*^*DTA*^, 2% tamoxifen was intraperitoneally injected once early in second telogen.

### Hair cycle staging

Male and female mice have different hair cycle lengths due to a longer telogen quiescence phase in females, but otherwise progress through the hair cycle similarly. In addition to sex, strain and individuals also affect hair cycle stages. Therefore, we always determine hair cycle stage by visual inspection, and morphological staging on sectioned tissue. Specifically, for C57BL/6 pure and mixed backgrounds, visual inspection was performed by trimming full-length telogen hairs with electric clippers to reveal dorsal skin. Hair follicle entry into anagen was determined by darkening of skin and reappearance of hair. Catagen progression was determined by lightening of the skin, which appears black at the end of anagen, to a near complete loss of pigmentation (greyish-pink skin) by late catagen. Entry into telogen was marked by the appearance of completely unpigmented (pink) skin. In unpigmented mice (CD1 strains), histological analysis of hair follicle morphology was used to confirm hair cycle staging based on relative age.

For all experiments, a small piece of midline dorsal skin was taken in parallel from each mouse, fixed and processed for sectioning and immunofluorescence to precisely stage the hair cycle. Hair cycle was staged based hair follicle morphology^50–53^, as well as by immunofluorescence for markers of anagen (EdU-incorporation following a 2 h pulse chase or Ki67 staining), catagen (cleaved caspase-3 and/or TUNEL positivity) or telogen (pSMAD1/5/9 and/or LEF1). For samples obtained from anagen or catagen stage mice the dorsal back skin was subdivided in two along the anterior-posterior axis prior to the experiment, as precise hair cycle stage differs anterior to posterior (with anterior generally one substage ahead). For anagen samples, 5′-ethynyl-2′-deoxyuridine (EdU) was injected intraperitoneally (50 μg g^−1^) (Sigma-Aldrich) and chased for 2 h prior to collection.

To assess hair cycling defects following ablation of HFSC-mediated apoptotic corpse clearance in catagen, cohorts of catagen-specific *Rxra* control and cKO mice were generated as described above. Similar cohorts of HX531 or annexin V intradermally injected wild-type CD1 mice were generated as described below. Both sets of mice were shaved and examined weekly over the course of second telogen for skin darkening (*Rxra* line) and hair regrowth (all experimental paradigms). Mice were collected upon initial signs of anagen re-entry (skin darkening and/or small hairs breaking skin surface). For the *Rxra* line, co-housed littermates were collected once one animal showed signs of anagen entry. Hair cycle was staged based on hair follicle morphology and immunofluorescence as described and compared across co-housed littermates (*Rxra* line) or between contralateral vehicle- and inhibitor-injected back skin within each mouse (intradermal manipulations).

### Induction and knockout constructs

#### RXRα induction

To make TRE-RXRa-Myc; pGK-H2B-RFP, human *RXRa* cDNA was PCR-amplified from pSV-Sport-RXRα (a gift from B. Spiegelman; Addgene #8882)^54^, and a NheI site was introduced at the 5′ end. This was then inserted into the NheI and EcoRI restriction sites of a pLKO vector modified to contain the inducible tetracycline response element at the 5′ end, as well as a 3′ MYC epitope tag. Prior to packaging this construct as a lentivirus, induction of RXRα was tested in culture using FACS-isolated *Krt14-rtTA*^+^ keratinocytes grown in E300 medium. In brief, cells were transiently transfected with the TRE-RXRα construct using Effectene into keratinocytes in a 6-well plate format, following the manufacturer’s protocol (Invitrogen). Forty-eight hours later, doxycycline (100 ng ml^−1^) was added to induce RXRα expression for a further 24 h. Cells were fixed and stained for Myc-tag and RXRa, as described for cell culture immunofluorescence.

#### RXRα-mosaic knockout

To identify efficient CRISPR single guide RNAs (sgRNA) against mouse *Rxra*, we synthesized oligonucleotides targeting exon 4 with BsmBI restriction sites at 5′ and 3′ respectively (IDT). Sequences used are available in Supplementary Table 6. Oligonucleotides were subcloned into pLentiGuide-Puro (a gift from F. Zhang; Addgene #52963), following the Zhang laboratory protocol^55^. To select a guide for in vivo use, we first tested the cutting efficiency in culture using *K14Cre*^+^; *R26*^*Cas9-EGFP*^ expressing keratinocytes. pLentiGuide-Puro constructs were transiently transfected using Effectene into keratinocytes in a 6-well plate format, following the manufacturer’s protocol (Invitrogen). After 72 h, genomic DNA was collected using QuickExtract DNA Extraction Solution (Lucigen), and guide DNA was prepared by heating to 65 °C for 10 min followed by heat inactivation at 95 °C for 2 min. Following PCR amplification of each guide target region, a T7 endonuclease I cutting assay (NEB) was used to identify the extent of insertions and/or deletions for each guide. The most efficient guide showed ~70% genome editing events in vitro, and was cloned with its U6 promoter into a modified pLKO vector containing a constitutive pGK-driven mScarlet fluorophore (3′ to the sgRNA) for lentiviral preparation.

#### RARE reporter

To identify cells responding to RA, we obtained pGL3-RARE-luciferase as a gift from T. M. Underhill (Addgene plasmid #13458; http://n2t.net/addgene:13458) and subcloned the RA response element (RARE) into a modified pLKO backbone behind a minimal SV40 promoter to drive RFP expression with pGK-driven H2B-GFP^56^.

#### *Cyp26b1* induction

To deplete active RA metabolites from cells inducibly, we over-expressed mouse *Cyp26b1* cDNA (Origene, MC205286) from a CAG promoter, interrupted by a Lox-Stop-Lox (LSL) cassette in a modified pLKO backbone. As a viral control, we used pGK-driven RFP in the opposite orientation^56^.

### Lentiviral preparation and Injection

High-titre lentivirus was prepared and E9.5 embryos of indicated genotypes were infected with lentivirus delivered by ultrasound-guidance microinjection into the amniotic sac as previously described^57,58^. At E9.5 the surface ectoderm exists as a single layer of unspecified skin progenitors, which can be efficiently, selectively and stably transduced by the viral DNA, without transduction of dermal cell types^58^.

### HFSC culture

All primary HFSC lines were grown on a layer of mitomycin C-inactivated 3T3/J2 feeder fibroblast cells, and maintained in E intermediate (300 μM) calcium media^59^ supplemented with 10 μM Y-27632 (Selleckchem) (E300-Y medium)^60^. The 3T3/J2 fibroblast cell line^59^ was expanded in DMEM/F12 medium (Thermo Fisher Scientific) with 10% CFS (Gibco), 100 U ml^−1^ streptomycin and 100 mg ml^−1^ penicillin. Cells were grown at 37 °C, with 7.5% CO_2_, and medium was routinely changed every 2–3 days. Cell lines were grown to confluency, then propagated by digesting with 0.25% Trypsin EDTA (Gibco) for 5–10 min at 37 °C and resuspended with culture medium for passaging. Experiments were conducted with cells at passages 8–10. For experiments, cells were switched to E intermediate calcium medium without Y-27632 (E300 medium) and cultured for 24–48 h prior to the experiment. All cell lines were maintained in a culture facility routinely testing negative for mycoplasma contamination.

Primary HFSCs were derived from the following mouse crosses at second telogen: *R26-mTmG*^*fl/+*^*; Sox9-creER*^*−*^ (mTomato^+^ HFSCs to make apoptotic corpses and necrotic debris), *R26-mTmG*^*fl/+*^*; Sox9-creER*^*+*^ (mGFP^+^ HFSCs to make naive HFSC to expose to corpses), *Rxra*^*+/+*^*; Sox9-creER*^*+*^*; R26-YFP*^*fl/fl*^ (*Rxra* wild type YFP^+^ HFSCs), and *Rxra*^*fl/fl*^*; Sox9-creER*^*+*^*; R26-YFP*^*fl/fl*^ (*Rxra* cKO YFP^+^ HFSCs). All HFSCs were FACS-isolated (described later) and cultured as described. To generate *Rxra* wild type and cKO HFSC lines, cells were FACS isolated (described later), and cultures were established prior to activation of *Sox9-creER by* 4-hydroxytamoxifen (4-OHT). At passage 2, *Sox9-creER* was activated in culture by 4-OHT in solution (Sigma Aldrich); to do so it was used at a final concentration of 1 μM in E300-Y medium to treat HFSCs. Medium plus 4-OHT was refreshed each day for three consecutive days, and then replaced by E300-Y. HFSCs were allowed to grow for 4 further days prior to FACS isolation of YFP^+^ cells. All HFSC lines, as well as the 3T3/J2 fibroblast line, were functionally and morphologically validated as HFSC or fibroblast lines respectively.

To generate HFSCs carrying the RARE-driven RFP with pGK-driven H2B-GFP (‘RARE reporter’), the RARE-reporter virus was transduced^57^ into wild-type primary HFSCs derived from a second telogen mouse, as described^56^. Stably integrated RARE-reporter HFSCs were FACS sorted on the basis of H2B-GFP, and RARE-driven RFP induction within 4-6 hrs was confirmed by addition of 100 nM 9cRA or 100 nM ATRA ±1 μM AGN 193109.

In pilot experiments nuclear accumulation (by immunofluorescence) of RXRα or RARγ peaked at 30 min post corpse exposure, and so that time point was used to assess immediate effects of corpse-derived signals or recombinant molecules in subsequent experiments. Similarly, the number of corpse-containing HFSCs plateaued at 4–6 h after corpse addition, and so transcriptional activation of the phagocytic programme, surface expression of phagocytic receptors and corpse engulfment (latter two by FACS) were routinely assessed at that time point (Supplemental Fig. 7).

To prepare corpses or secondarily necrotic debris, fully confluent mTomato^+^ HFSCs were treated with 200 μM cisplatin (in 0.9% saline) for 18 h (apoptotic corpses) or 48 h (necrotic debris). Dead and dying cells were collected from the supernatant by pelleting at 700*g* for 5 min, washed once with E300 medium, and returned to the plate in fresh E300 medium. Prior to returning the floating corpses, dying adherent cells were rinsed with PBS to remove residual cisplatin and a minimal amount of fresh E300 medium was added. Corpses or necrotic debris were allowed to condition the medium for a further 3–4 h. Apoptotic cell corpses were collected by tapping the side of the plate and pipetting their medium over them to detach dying cells. Floating and detached corpses were collected and pelleted by centrifugation as before. Corpse-conditioned medium was carefully removed to a separate tube, before resuspending corpses in a minimal volume of fresh E300 medium. To label any corpses/debris derived from 3T3/J2 fibroblasts, corpses were next incubated with DiI-CM (Invitrogen) for 5 min at 37 °C before pelleting and washing with PBS as before. DiI/mTomato^+^ corpses were resuspended in their corpse-conditioned medium, counted and aliquoted (in their conditioned medium) directly onto experimental plates (medium removed prior) at a ratio of roughly 10 corpses:1 HFSC. For corpse-conditioned medium experiments, corpses were prepared as described and then spun out of the medium at 1,200*g* for 10 min. Corpse-conditioned medium was further strained through a 0.45-μm syringe filter, prior to use. Corpses, necrotic debris and/or conditioned medium were always prepared immediately prior to their use.

Manipulation of corpse-derived signals was achieved by adding small molecule inhibitors to the corpses after the removal of cisplatin (16 μM BEL (Sigma Aldrich); 100 nM MPA08 (Tocris Bioscience)) or by incubating the corpses with recombinant molecules for 15-20 min prior to adding them to naive HFSCs (1 U Apyrase; 1 ng ml^−1^ annexin V (both Tocris Bioscience)). Vehicle treated control corpses were incubated with 1% DMSO. Similar preparations were made for corpse-conditioned medium. To manipulate corpse-sensing mechanisms on HFSCs, naive mGFP^+^ or YFP^+^ or RARE-reporter HFSCs were pretreated with the indicated antagonist (1 nM UVI3003; 1 μM HX531; 1 μM JTE013; 100 nM BMS 777607; 1 μM AGN 193109) (first four: Tocris Bioscience; last one: R&D Systems) for 30 min prior to corpse or corpse-conditioned medium addition. When adding corpses or conditioned medium, the concentration of antagonist was maintained by adding an additional amount of the appropriate compound to the corpses and/or conditioned medium. Experiments were performed in biological duplicate or triplicate and repeated at least twice on separate days. For data visualization, all replicates across independent experiments are represented, unless indicated otherwise in figure legends. Experiments manipulating corpse-derived signals by small molecule inhibitors were performed in parallel, such that a core set of control medium and corpses + Veh experimental replicates exist. For presentation purposes, small molecule inhibitors were grouped according to the step of phagocytosis they affect in separate figure panels, and so the core set of control medium and corpses + Veh is repeated for each (Fig. 4b and Extended Data Figs. 3g, 6d and 7a).

To test the ability of recombinant molecules to recapitulate corpse secreted signals, mGFP^+^ or YFP^+^ or RARE-reporter HFSCs were cultured in E300 medium plus the indicated concentrations of recombinant molecules (see figures and figure legends). Molecules were prepared and stored as stock solutions according to manufacturer’s instructions. In brief, 9cRA and ATRA (both R&D Systems) were each dissolved in 100% DMSO, protected from light, and stored long term at −80 °C with working solutions kept at −20 °C. Recombinant LPC, S1P and AA (all Tocris Bioscience) were each dissolved in 100% ethanol and stored like the retinoids. Free nucleotides were purchased as 100 mM stocks of dATP or dUTP as sodium salts in ultrapure water (NEB) and stored at −20 °C. Stock solutions were diluted individually or in combinations with E300 medium for experiments. Experiments were performed in biological duplicate or triplicate and repeated at least twice on separate days. For data visualization, all replicates across independent experiments are represented, unless indicated otherwise in figure legends. Experiments testing different concentrations and combinations of recombinant molecules for induction of RXRα^+^ or RARγ^+^ nuclear accumulation in cultured HFSCs were performed in parallel, such that a core set of control medium experimental replicates exist. For presentation purposes, sets of concentrations were split across separate figure panels and so the core set of medium control experiments are repeated in each (Fig. 4d and Extended Data Fig. 7e).

To examine the effects of repeated exposure to necrotic damage in vitro, conditioned medium was prepared from live HFSCs, apoptotic corpses or necrotic debris as described above. For proliferation studies, naive mGFP^+^ HFSCs were exposed to new conditioned medium daily three times in 96-well plates on fibroblast feeders and cells were counted by GFP fluorescence in a BioTek Cytation 5 cell imaging multimode reader. Experiments were set up in three biological replicates, and cell numbers were counted daily. Experiment was performed independently three times with one representative experiment shown. To assess the effect of repeated exposure to necrotic debris on colony forming efficiency and size, naive mGFP^+^ HFSCs were plated in 12-well plates on fibroblast feeders and exposed to conditioned medium as described for proliferative studies. Twenty-four after the third exposure to conditioned medium, HFSCs were collected by trypsinization, washed with PBS and replated on fibroblast feeders in E300-Y medium at a density of 10,000 HFSCs per replicate. Medium was replaced 4 days later and changed every second day after that for a total of 14 days of growth. Colony number and size were quantified by GFP fluorescence in a BioTek Cytation 5 cell imaging multimode reader. Experiment was performed twice in experimental triplicates and all six replicates shown.

For colony forming assays on HX531- or annexin V-injected mice, primary HFSCs were FACS-isolated from either uninjected wild type or contralateral skin sites of intradermally injected vehicle versus inhibitor mice as described below. Isolated HFSCs were stained with DiI-CM as described for corpse preparation prior to plating in technical triplicates of 2,000 HFSCs each on 3T3/J2 feeder fibroblasts in 6-well plates in E300-Y medium and allowed to grow for 4 days before medium was changed. At one week post-seeding, colony number and size were counted under an upright fluorescent microscope using bright field and RFP (DiI-CM) fluorescence. Technical triplicates were averaged, and data presented per mouse.

### Intradermal injections

Adult mice in early second catagen (CatII) were anaesthetized using isoflurane prior to intradermal injections. Isofluorane anaesthetization was maintained throughout the procedure using a nose cone for delivery. Back skin was shaved using electric clippers and the surface sterilized by wiping with ethanol wipes. Vehicle or small molecule containing solutions were prepared by diluting appropriate chemical in sterile PBS plus 1% FluoSpheres Carboxylate-modified microspheres (1.0 μm, Thermo Fisher Scientific F8816) to assess placement of the injection on tissue sections. To find the injection site for repeated intradermal injections on subsequent days, a small dot was made with permanent marker which the needle was inserted through. One-millilitre insulin syringes with the needle bent to approximately 45° were used to shallowly inject through the epidermis to the dermal space approximately 3–5 mm from the injection site. An injection volume of 25 μl was delivered per injection site, with an average of 4 injection sites per mouse: two anterior and two posterior. Vehicle injections (10% DMSO) were randomly designated to either the left or right side, with the contralateral skin receiving the indicated small molecules. Following the injections, mice were placed in their home cage on a heating pad to recover. Compounds were prepared at 100× the cell culture working solution, from the same stock solutions. To inhibit the phagocytic programme across the course of catagen, intradermal injections were performed three times, separated by 20–24 h. Mice were euthanized by lethal CO_2_ administration, 4 h after the final injection (at CatVII–VIII), or allowed to progress into telogen for further analysis. For colony-forming assays, injected mice were euthanized 2 days after the end of catagen, back skin was manually dissected into 10 mm^2^ around sites of injections and HFSCs were FACS isolated as described below. Isolated HFSCs were plated on feeders and analysed as described for colony-forming assays (above). To analyse hair cycling defects upon transient inhibition of corpse engulfment during catagen, intradermally injected mice were followed throughout the course of second telogen as described above.

### Tissue collection and sectioning

For immunofluorescence analysis of tissue sections, mice were shaved following lethal CO_2_ administration and their back skin dissected. Back skin was stretched onto Whatman paper for stability, and immediately prefixed in 1% or 4% paraformaldehyde (PFA) for 1 h at 4 °C or 30 min at 25 °C, respectively. After fixing, tissue was washed twice with PBS for 10 min at 4 °C, before incubating in 30% sucrose in PBS at 4 °C overnight. Tissue was embedded in OCT medium (VWR) and frozen on dry ice blocks before storage at −80 °C. Alternatively, fresh frozen tissue was prepared without prefixation by directly embedding the skin in OCT after it was placed on Whatman paper. Frozen tissue blocks were sectioned at 20 um on a Leica cryostat and mounted on SuperFrost Plus slides (Thermo Fisher). When necessary, sections were stored at −20 °C prior to use.

### Immunofluorescence

#### Skin sections

Following sectioning, tissue was allowed to dry on the slide for 1 h in a partially closed slide box. Fresh frozen tissue was post-fixed with 4% PFA for 5 min, followed by washing in phosphate-buffered saline (PBS) three times for 5 min each. Pre-fixed tissue sections started with the PBS wash step to remove attached Whatman paper. Following washes, samples were permeabilized and blocked in blocking buffer (5% donkey serum, 2.5% fish gelatin, 1% BSA, 0.3% Triton in PBS) for 1 h at room temperature. Primary antibodies were incubated overnight at 4 °C, samples were washed for 5 min in PBS (three times) at room temperature, and secondary antibodies were incubated together with DAPI (to label nuclei) for 1 h at room temperature. Following three final PBS washes of 5 min each, samples were mounted in Prolong Diamond Antifade Mountant (Invitrogen) for imaging. For TUNEL labelling, the Cell Death Detection Kit (TMR red or FITC; Roche) was used according to manufacturer’s instructions, with application of secondary antibodies. A modification was made to halve the concentration of the substrate labelling component to reduce background fluorescence in the skin. For phospho-STAT3 staining, tissue was incubated in ice-cold methanol for 20 min at −20 °C, followed by three times PBS washes prior to blocking. Antibodies were used as follows: rabbit anti-cleaved-caspase-3 (Cell Signaling, 9661, 1:250), rat anti-RFP (Chromotek, 5F8, 1:1,000), rabbit anti-RFP (MBL, PM005, 1:1,000), chicken anti-GFP/YFP (Abcam, ab13970, 1:1,000), goat anti-P-cadherin (R&D, AF761, 1:250), rabbit anti-keratin14 (Fuchs laboratory, 1:200), rabbit anti-keratin24 (Fuchs laboratory, 1:200), sheep anti-Ki67 (Novus Biologicals, AF7649, 1:200), rabbit anti-MYC epitope (71D10) (Cell Signaling, 2278, 1:250), rat biotinylated anti-CD45 (Biolegend, 5530, 1:200), rabbit anti-RXRα (D6H10) (Cell Signaling, 3085, 1:250), rabbit anti-RARγ (D3A4) (Cell Signaling, 8965, 1:250), rabbit anti-MFGE8 (Invitrogen, PA5-109955, 1:200), rat AlexaFluor647-conjugated anti-F4/80 (BM8) (Biolegend, 123121, 1:200), rat biotinylated anti-ITGA6 (also known as CD49f) (GoH3) (Biolegend, 313603, 1:500), rabbit anti-cJun (60A8) (Cell Signaling, 9165, 1:250), rabbit anti-FosB (5G4) (Cell Signaling, 2251, 1:250), and rabbit anti-phospho-STAT3 (Tyr705)(D3A7) (Cell Signaling, 9145, 1:250). All secondary antibodies used were raised in a donkey host, and conjugated to AlexaFluor488, Rhodamine, or AlexaFluor647 (Jackson ImmunoResearch Laboratory; 1:500). Catalogue numbers (given in order of: AlexaFluor488, Rhodamine, and AlexaFluor647 conjugates) for donkey anti-rabbit antibodies (711-545-152; 711-295-152; 711-605-152), for donkey anti-rat antibodies (712-545-150; 712-295-150; 712-605-150), for donkey anti-chicken antibodies (703-545-155; 703-295-155; 703-605-155), for donkey anti-goat antibodies (705-545-003; 705-295-003; 705-605-003), and for donkey anti-sheep AlexaFluor647 (713-605-003). 4′,6-diamidino-2-phenylindole (DAPI) was used to label nuclei (1:10,000). To co-stain RARγ and RXRα, the rabbit primary antibodies were individually directly conjugated to one of AlexaFluor350, AlexaFluor488, AlexaFluor568, or AlexaFluor647 using the rabbit specific Zenon Antibody Labelling Kit (Thermo Fisher Scientific) and following manufacturer’s instructions.

#### Iterative bleaching extends multiplexity

Tissue sections were processed, fixed and sectioned as for normal immunofluorescence, with one modification. Tissue cryosections (25-μm) were placed in glass bottom slide wells coated with chrome gelatin alum to securely adhere the tissue to the glass coverslip. The IBEX protocol was followed as described^61^, with the following modifications. Following blocking with our blocking buffer (above), tissue was incubated with primary antibodies directly conjugated to fluorophores for 3 h at room temperature, followed by PBS washes and imaging using a spinning disk confocal microscope. DAPI was used as described before, as a fiducial stain to align images from iterative cycles. To bleach fluorophores between iterative cycles of staining and imaging, we exposed tissue to 1 mg ml^−1^ of lithium borohydride for 15 min at room temperature, followed by three 1× PBS washes. Antibodies used were as follows: (panel 1) rat anti-Foxp3-AlexFluor488 (FJK-16s) (ThermoFisher, 53-5773-82, 1:100), In situ cell death detection kit, TMR red (Roche), rat anti-CD8-AlexaFluor647 (BioLegend, 100724, 1:150); (panel 2) rat anti-CD206-AlexaFluor488 (MMR) (BioLegend, 141710, 1:500) and rat anti-CD68-AlexaFluor647 (BioLegend, 137004, 1:500); (panel 3) rat anti CD11c-AlexaFluor488 (N418) (BioLegend, 117311, 1:100) and rat anti-Ly6g-AlexaFluor647 (1A8) (BioLegend, 127610, 1:150); (panel 4) rat anti-ITGA6-AlexaFluor488 (BioLegend, 313608, 1:150) and rat anti-Langerin–AlexaFluor647 (929F3.01) (Novus Biologicals, DDX0362A647-100; 1:100); (panel 5) rat anti F4/80-AlexaFluor488 (BioLegend, 123122, 1:150) and rat anti-CD172a (Sirpα)-AlexaFluor647 (BioLegend, 144028, 1:150); (panel 6) hamster anti-TCRgd-AlexaFluor488 (BioLegend, 118128, 1:100) and rat anti-Tim4-AlexaFluor647 (RMT4-54) (BioLegend, 130008, 1:150); (panel 7) rat anti-CD4-AlexaFluor488 (RM4-5) (BioLegend, 100529, 1:100) and rat anti-CD3-AlexaFluor647(17A2) (BioLegend, 100209, 1:100); (panel 8) Avidin–FITC (ThermoFisher Scientific, A821 1:1,000) and rat anti-I-A/I-E (MHCII)–AlexaFluor647 (M5/114.15.2) (BioLegend, 107618, 1:150); (Panel 9) rat anti-CD45-AlexaFluor488 (BioLegend, 103122, 1:150) and rat anti-P-cadherin-AlexaFluor647 (R&D Systems, FAB761R-100UG, 1:200).

#### Cell culture

For immunofluorescence experiments, feeders were split onto poly-l-lysine coated glass coverslips, seeded in 12-well plates 24 h prior to the addition of HFSCs. HFSCs were grown to confluency before feeders were detached by repeated PBS washes, and corpse or corpse-conditioned medium experiments were performed. At the end of the experiment, cells were washed twice with PBS and prefixed with 4% PFA for 3 min at 25 °C. Cells were washed three times with PBS and stained as for tissue sections.

### Microscopy

Images of *Sox9-creER; Rosa26*^*Brainbow2.1*^ tissue was acquired using a Zen-software driven Zeiss LSM 780 inverted laser scanning confocal microscope and 20× air objective (NA = 0.8), a 40× water immersion objective (NA = 1.2), or a 63× oil immersion objective (NA = 1.4). To separate CFP, YFP, GFP, RFP and AlexaFluor647 fluorophores, excitation with specific laser lines (405, 440, 488, 514, 561, 594, and 633) and narrow wavelength emission cut-offs on 4 detectors were set up as follows: CFP (excitation 440 nm, emission 450 nm–490 nm), GFP (excitation 488 nm, emission 500 nm–515 nm), YFP (excitation 514 nm, emission 525 nm–570 nm), RFP (excitation 561 nm, emission 595 nm–620 nm), and AlexaFluor647 (excitation 633 nm, emission 650 nm–690 nm). Due to their well-separated excitation and emission spectra, GFP and AlexaFluor647 were acquired simultaneously on the same detector. Stacks with a 1-μm step were acquired. Confocal microscopy was performed in The Rockefeller University’s Bio-Imaging Resource Center, RRID: SCR_017791.

Images of *Rxra*^*fl*^*; Sox9-creER; Rosa26*^*YFP*^ tissue stained using IBEX methodology was acquired on an inverted Dragonfly 202 spinning disk confocal system (Andor Technology Inc.) using the 40× oil immersion objective, a 40-μm pinhole and a Zyla camera. Four laser lines (405, 488, 561 and 625 nm) were used for near simultaneous excitation of DAPI, Alexa-448, RRX and Alexa-647 fluorophores. Tiled images with a 1-μm stack step were acquired using the Andor Fusion software (v 2.3). Images were stitched and aligned using DAPI as a fiducial with Imaris and the SimpleITK Image Registration Pipeline plug-in for Imaris.

Images of other cryosections were acquired using a Zen-software-driven Zeiss Axio Observer.Z1 epifluorescence/brightfield microscope with a Hamamatsu ORCA-ER camera, Axiocam350, and an ApoTome.2 slider (to reduce light scatter in *z*). Stacks with a 1-μm step were acquired. Apotome acquired images were processed via ‘Apotome Raw Convert’ function, and stitched (if necessary), in Zen software (v 3.1). Subsequent image processing was conducted in ImageJ (v. 2.9.0) and Imaris (v. 10.1) (Oxford Instruments) software. For presentation purposes, images were cropped and assembled in Adobe Illustrator.

### Phenotyping corpse engulfment via microscopy

To quantify apoptotic cell clearance in tissue sections, confocal images were acquired with a 40× or 63× oil immersion objective, with a 1-μm step size, of tissue stained with either P-cadherin or KRT14 to mark cell boundaries, DAPI to mark nuclei, and TUNEL to label late-stages of cell death. On single *z*-plane images, dying cells were scored as engulfed when a small TUNEL+ apoptotic body was nestled inside the cell boundary of a cell with a healthy nucleus, and could be visualized as such across the consecutive *z*-stack. Apoptotic bodies were generally round and found against health nuclei, in accordance with electron microscopy images of wild-type hair follicle ORS cells. Unengulfed apoptotic cells were either large TUNEL+ signal that completely overlapped a condensed nucleus and occupied roughly 50–75% the area of a healthy cell, or were visualized as small, irregularly shaped TUNEL+ debris pushed to the cell boundary edges. TUNEL+ debris was slightly more prevalent at the dermal–epithelial junction or the epithelia directly adjacent to the hair shaft, but was also visible throughout the ORS at cell boundaries.

### Electron microscopy

Dissected back skin was placed on thin paper towel for stability, and fixed in 2% glutaraldehyde, 4% PFA, and 2 mM CaCl_2_ in 0.1 M sodium cacodylate buffer (pH 7.2) for 2 h at room temperature, postfixed in 1% osmium tetraoxide and processed for Epon embedding. Ultrathin sections of 60-65 nm were counterstained with uranyl acetate and lead citrate, before images were taken with a transmission electron microscope (Tecnai G2-12;FEI) equipped with a digital camera (AMT BioSprint29). Samples were processed and imaged at The Rockefeller Electron Microscopy Resource Center. The number of engulfed apoptotic corpses per stem cell was quantified via transmission electron micrographs.

### Flow cytometry

To obtain single-cell suspensions for fluorescence activated cell sorting (FACS) at all stages of the hair cycle, back skin was excised, and the dermal side scraped with a dull scalpel to remove excess fat prior to incubation with 0.25% collagenase (Sigma-Aldrich) in warm PBS, dermal side down for 45–60 min at 37 °C with gentle rotation in a plastic petri dish. The dermal side was scraped gently with a dull scalpel to mechanically dissociate cells in the lower ORS and hair bulb (“dermal fraction”). The dermal fraction was only kept for late anagen and early-to-mid catagen samples, and was processed separately from the epidermal fraction. To collect the epidermal fraction, the skin was placed dermal side down in 0.25% trypsin-EDTA (Gibco) for 20–25 min at 37 °C with gentle rotation. The hairy side of the skin was scraped against the direction of hair growth with a dull scalpel to release cells in the upper hair follicle (including the hair follicle bulge stem and hair germ progenitor cells). For both dermal and epidermal fractions, the resulting cell suspensions were pipetted up and down with a 5 ml serological pipette for 5 min, before being quenched with FACS buffer (5% fetal bovine serum, FBS, in PBS). Plastic petri dishes were rinsed with 5 ml of FACS buffer 2–3 times, which was collected and added to the appropriate cell suspension. Suspensions were filtered through sequential 70-μm and 40-μm nylon filters (VWR), before being pelleted at 350*g* for 15 min at 4 °C. Cell pellets were resuspended in ice cold FACS buffer, re-filtered into FACS tubes, and incubated with primary antibodies for 20 min on ice. Secondary antibodies and LysoTracker DeepRed (Invitrogen, 1:4,000) were added directly to FACS tubes, and incubation continued for 10 min on ice. Samples were further diluted with FACS buffer plus DNase (Roche) to minimize cell clumping prior to sorting or analysis. For analysis of RXRα levels by FACS, cells were stained with cell-surface specific primary and secondary antibodies, before being fixed and processed using the BD Cytofix/Cytoperm kit following manufacturer’s instructions. Primary antibodies were used as follows: rat biotinylated anti-CD45 (30-F11) (eBioscience, 13-0451-82, 1:200), rat biotinylated anti-CD117 (2B8) (eBioscience, 13-1171-82, 1:200), rat biotinylated anti-CD140a (APA5) (eBioscience, 13-1401-82, 1:200), rat biotinylated anti-CD31 (390) (eBioscience, 13-0311-82, 1:200), rat anti CD34-FITC (RAM34) (eBioscience, 11-0341-82, 1:200), rat anti CD34–eFluor660 (RAM34) (eBioscience, 50-0341-82,1:200), rat anti ITGA6–PercpCy5.5 (GoH3) (BioLegend, 313617, 1:250), rat anti-Ly6A/E-APC-Cy7(BioLegend, 108125, 1:1,000), rabbit anti-RXRα (D6H10) (CST, 3085, 1:250), rat anti-Tyro3/Dtk-AlexaFluor700 (R&D Systems, FAB759N, 1:200), rat anti-Mertk-AlexaFluor700 (R&D Systems, FAB5912N, 1:200), and rat anti-Axl-AlexaFluor700 (R&D Systems, FAB8541N, 1:200). Secondary antibodies were used as follows: Strepavidin-PE-Cy7 (1:3,000) and donkey AlexaFluor 488 or AlexaFluor568 (1:500). Annexin V-AlexaFluor568 (Invitrogen, A13202, 1:100) and/or DAPI was used to identify apoptotic and dying cells, respectively. For FACS using annexin V, primary and secondary antibody staining was performed in annexin V Binding Buffer (10 mM HEPES, 140 mM NaCl, 2.5 mM CaCl_2_, pH 7.4). For AldeFluor activity assay, manufacturer’s instructions were followed (ALDEFLUOR Kit 01700, StemCell Technologies) with the addition of a more specific ALDH1A inhibitor at 1 μM (673-A, R&D Systems 6934).

For FACS analysis, live cell suspensions from back skin were collected and analysed as described above. Alternatively, cultured HFSCs were trypsinized for 7–10 min (as for passaging the cell lines), and pelleted at 300*g* before resuspension, filtering and incubating with primary antibodies. A minimum of 20,000 HFSCs were analysed per sample using either a BD LSRII Flow Cytometer or a BD Fortessa Flow Cytometer (BD Bioscience). Representative sort schemes pertaining to analysis of TAM-family receptors, LysoTracker expression, RXRa expression and the AldeFluor assay can be found in Supplementary Figs. 3–5 in combination with Extended Data Figs. 4 and 8. For analysis of the Brainbow2.1 HFSCs, a LSRII specially equipped with a 445 nm laser was used to excite CFP, separately from YFP/GFP and RFP. Phagocytic HFSCs were scored as double positive (containing a corpse of one fluor inside a cell of another fluor) following stringent gating against doublets, and separately confirmed as engulfment events via immunofluorescence. A representative sort scheme is shown in Supplementary Fig. 1.

For HFSC isolation for single cell RNA-sequencing cells were sorted according to the scheme shown in Supplemental Fig. 2, with an 85-μm nozzle into 96-well PCR plates (Bio-Rad) containing 2 μl of lysis buffer (0.2% Triton X-100, 2 U μl RNaseOUT (Thermo Scientific), 0.25 μM oligo-dT30VN primer, 1:2 × 10^6^ diluted ERCC spike-in RNAs (Ambion)). For HFSC isolation for in vitro culture and bulk ATAC-sequencing, cells were sorted using a 70-μm nozzle into E300-Y medium and FACS buffer (Supplementary Fig. 3). Representative sort schemes pertaining to *Sox9-creER;R26*^*DTA*^ ectopic corpse response and RXRα overexpression and knockout in HFSCs, are available in Supplementary Figs. 4 and 5, respectively. To isolate primary HFSCs for culture from the *Sox9-creER:mTmG*^*fl/+*^ treated or not with tamoxifen as described previously, we gated on mTomato^+^ or mGFP^+^ in combination with CD34^+^ITGA6^+^ (Supplementary Fig. 6), using a 70-μm nozzle to sort into FACS buffer. Similar methodology was used to isolate *Rxra* HFSC lines for culture as described in Supplemental Fig. 3, after which *Sox9-creER* was activated with 4-OH-tamoxifen in culture. For bulk RNA-sequencing, cells were sorted using a 70-μm nozzle directly into Trizol and FACS buffer (Supplementary Fig. 7). Sorting was performed on a BD FACSAriaII equipped with Diva software (v. 8.0) (BD Biosciences).

Flow cytometry was performed at The Rockefeller University’s Flow Cytometry Resource Center (RRID: SCR_017694). Flow cytometry plots were generated using FlowJo to illustrate the strategies used for cell isolation, and manually compensated for presentation.

### scRNA-sequencing libraries

Single-cell RNA-sequencing libraries were prepared from FACS-isolated hair follicle epithelial cells in AnaVI, CatVI, and CatVII, using a slightly modified Smart-Seq2 protocol as previously described^62,63^. For each hair cycle stage, cells from 3–6 mice were pooled prior to FACS isolation. In brief, cells were sorted into hypotonic lysis buffer, snap frozen in liquid nitrogen and stored at −80 °C until all samples were collected. Cells were lysed by heating at 72 °C for 3 min, followed by reverse transcription of mRNA using dT30 oligonucleotides, template switching oligonucleotides and Maxima H- reverse transcriptase. The whole transcriptome was amplified (15 cycles) by KAPA HiFi DNA polymerase (Roche), and then size-selected using 0.6× AmpPure XP beads (Beckman Coulter). To exclude cells with poor amplification, and wells containing multiple cells, quantitative PCR (qPCR) for *Gapdh* was performed. Illumina sequencing libraries were indexed with unique 5′ and 3′ barcode combinations (up to 384 cells) using the Nextera XT DNA library preparation kit (Illumina). Libraries were pooled and size-selected with 0.9× AmpPure XP beads. Prior to sequencing on Illumina NextSeq500 using a 75 bp paired-end read mid-output setting, library quality was assessed by TapeStation (Agilent).

### ATAC-sequencing libraries

ATAC-seq was performed on 20,000–75,000 (in vivo samples) or 50,000 (culture samples) FACS-sorted HFSCs, as previously described^64–66^. In brief, cells were lysed in ATAC lysis buffer for 1 min on ice, washed and nuclei resuspended in transposase buffer. Genomic DNA was transposed using Tn5 transposase (Illumina) for 30 min at 37 °C, at which point the reaction was halted. Samples were uniquely barcoded in batches of 10-12 (in vivo samples) or one batch of 27 (cultured HFSCs), using Buenrostro^64^ or Nextera XT index kit v2 indices. Sequencing libraries were prepared according to manufacturer’s instructions (Illumina). Libraries were sequenced to a depth of 50–100 million sequences, using paired-end runs on an Illumina Novaseq 6000 (at The Rockefeller University Genomics Resource Center).

### CUT&RUN sequencing libraries

CUT&RUN sequencing was performed on 500,000 cultured HFSCs, as previously described with minor modifications^67,68^. Unless otherwise indicated, steps were performed at room temperature. In addition to biological samples, antibody validation and specificity was verified using (1) a rabbit IgG control antibody; and (2) rabbit anti-RXRα in the *Rxra-*cKO HFSCs. In brief, cells were trypsinized as for re-plating, then washed with PBS and resuspended in crosslinking buffer (10 mM HEPES–NaOH pH 7.5, 100 mM NaCl, 1 mM EGTA, 1 mM EDTA and 1% formaldehyde) with rotation for 10 min. Crosslinked cells were quenched with 0.125 M (final concentration) glycine for 5 min, then washed with ice cold 1× PBS and resuspended in NE1 buffer (20 mM HEPES–KOH pH 7.9, 10 mM KCl, 1 mM MgCl_2_, 1 mM dithiothreitol, 0.1% Triton X-100 supplemented with Roche complete protease inhibitor EDTA-free) and rotated for 10 min at 4 °C. Nuclei were washed twice with CNR wash buffer (20 mM HEPES pH 7.5, 150 mM NaCl, 0.5% bovine serum albumin and 0.5 mM spermidine supplemented with protease inhibitor) and incubated with concanavalin-A (ConA) beads washed with CNR binding buffer (20 mM HEPES–KOH pH 7.9, 10 mM KCl, 1 mM CaCl_2_ and 1 mM MnCl_2_) for 10 min at 4 °C. ConA-bead bound nuclei were incubated overnight at 4 °C in CNR antibody buffer (CNR wash buffer supplemented with 0.1% Triton X-100 and 2 mM EDTA) and 1:50 RXRα antibody (Cell Signaling Technologies, clone D6H10, 3085). ConA-bead bound nuclei were washed with CNR Triton wash buffer (CUT&RUN wash buffer supplemented with 0.1% Triton X-100) then resuspended and incubated at 4 °C for 60 min in CUT&RUN antibody buffer and 2.5 μl pAG-MNase (EpiCypher). Following this, ConA-bead bound nuclei were washed twice with CUT&RUN Triton wash buffer, resuspended in 100 μl of Triton wash buffer and incubated on ice for 5 min before 2 μl of 100 mM CaCl_2_ was added per sample. Samples were incubated on ice for 30 min and the reaction was then stopped by adding 100 μl of 2× stop buffer (340 mM NaCl, 20 mM EDTA, 4 mM egtazic acid, 0.1% Triton X-100 and 50 μg ml^−1^ RNaseA) and incubated at 37 °C for 10 min. ConA-bound nuclei were captured on a magnet, and supernatant containing Cut-and-Run DNA fragments was collected. Supernatant was incubated at 70 °C for 4 h with 2 μl 10% sodium dodecyl sulfate and 2.5 μl 20 mg ml^−1^ proteinase K, prior to DNA purification using PCI reagent (phenol:chloroform:isoamyl alcohol, Millipore). DNA fragments were precipitated overnight with ethanol and glycogen at −20 °C before resuspension in elution buffer (1 mM Tris–HCl pH 8.0 and 0.1 mM EDTA).

CNR sequencing libraries were generated using NEBNext Ultra II DNA Library Prep Kit for Illumina and NEBNext Multiplex Oligos for Illumina. PCR-amplified libraries were purified using 1× ratio of SPRI beads (Beckman) and eluted in 15 μl EB buffer (Qiagen). All CNR libraries were sequenced on Illumina NextSeq using 40 bp paired-end reads.

### RNA isolation

Total RNA was isolated from FACS-isolated HFSCs using the Direct-zol RNA MicroPrep kit (Zymo Research) following manufacturer’s instructions. The optional DNase I treatment was included in all sample preps, and RNA was eluted in DNase/RNase-free water. Quality and concentration of RNA samples were determined using an Agilent 2100 Bioanalyzer. All samples for sequencing had RNA integrity (RIN) numbers >8.5. RNA samples were used for qPCR with reverse transcription (RT–qPCR) or bulk RNA sequencing, as described.

### Bulk RNA-sequencing libraries

Comparable amounts of RNA per sample were used to prepare bulk RNA-sequencing libraries using Illumina Trueseq standard mRNA library kit (non-stranded, poly-A selection) following manufacturer’s guidelines. Libraries were then uniquely barcoded, pooled and sequenced on an Illumina Novaseq 6000 using single-end runs (at Weill Cornell Medical College’s Genomic Core Facility).

### RT–qPCR

Equivalent amounts of RNA were reverse transcribed using SuperScript III Reverse Transcriptase (Thermo Fisher Scientific) following manufacturer’s instructions. To normalize cDNA amount across samples, primers for *B2m* were used. cDNAs were mixed with gene specific primers (Supplementary Table 6) and SYBR green PCR MasterMix (Sigma Aldrich) and run on an Applied Biosystems 7900HT Fast Real-Time PCR system.

### Single cell and bulk RNA-sequencing analysis

Trimmed FASTQ files were obtained from the Rockefeller University’s Genome Resource Center (scRNA-sequencing, this study), or from the Gene Expression Omnibus (GSE90848 and GSE130850 for previously published telogen and AnaI-II HFSC scRNA-sequencing datasets), or from the Genomic Core Facility (Weill Cornell Medical College; bulk RNA-sequencing), and raw sequencing reads were aligned to the mouse reference genome (UCSC release mm39) using STAR (v2.6)^69^. The expression values of each gene were quantified as both raw counts and transcripts per million (TPM) using Salmon (v.1.4.0)^70^, and compiled in R (v.3.6.1) using RStudio (v.3.4.2) by Tximport (v.1.12.3)^71^.

#### Bulk RNA sequencing

For differential gene expression analysis in R, low detection genes (minimum average read count <10) were filtered before DESeq2 analysis (v.1.16.1)^72^. Differential expression modelling used a negative binomial distribution and Wald test. Genes were differentially expressed for log_2_[fold-change]>|1| and adjusted *P* < 0.05. Heat maps and bar graphs illustrating differential gene expression were constructed in a Python environment (detailed in next paragraph).

#### scRNA-sequencing

Analysis and visualization of the data were conducted in a Python environment built on Pandas (v.2.0.1), NumPy (v.1.24.2)^73^, SciPy (v.1.10.1)^74^, scikit-learn (v.1.2.0), SCANPY (v1.9.3)^75^, AnnData (v.0.9.1)^75^, matplotlib (v.3.7.1)^76^ and seaborn (v.0.13.1)^77^ packages. Raw count and metadata matrices for 1,489 single ORS cells across the hair cycle were loaded in SCANPY as an AnnData object. Single cell data was preprocessed to remove lowly detected genes (expressed in <75 cells) and cells with low complexity libraries (<2,000 genes detected). SCANPY was used to normalize counts per cell, and highly variable genes were detected. Prior to dimensionality reduction by principal component analysis (PCA), data were centred and scaled. PCA was performed on highly variable genes, with 100 components and the svd_solver using ‘arpack’ (SCANPY default setting). To construct a *k*-nearest neighbours graph on Euclidean distance, 41 principal components were used (which captured 25% of the variance in the data). Data was visualized using UMAP in SCANPY, and clustering was done using the Leiden algorithm (with a resolution setting of 0.5). Cluster resolution was chosen after iterating through resolution parameters from 0.1 to 0.75, as best capturing both hair follicle cycle stages and anatomic location (upper bulge region/upper ORS versus hair germ/upper-middle ORS versus lower ORS). Marker gene expression based on the literature^7,11,63,78^, together with the FACS markers each population was sorted on, was used to identify clusters. SCANPY was used to visualize selected marker genes in dot plots, or as normalized counts visualized on UMAPs.

Differential gene expression based on cluster identity was used in DESeq2 to identify genes that varied as cells transitioned from late anagen growth phase to catagen. Differential expression was performed as described for bulk RNA-sequencing, with the modification of a threshold of 0.75 to construct Wald tests of significance. Gene set enrichment analysis (GSEA) on differentially expressed genes was performed using GSEA software (v.4.3.2)^79,80^, and run with the MSigDB 2022 mouse database. Gene set terms with false discovery rate < 0.1 and showing high normalized enrichment scores in catagen cells were considered interesting. To construct gene set scores based on the GSEA identified terms the corresponding *Mus musculus* gene lists were obtained by Amigo2 through the Gene Ontology consortium. The SCANPY tl.score_genes function was used to compute the average expression of each gene set across single cells, and normalized to a randomly sampled reference set of genes^81,82^. The resulting gene set scores were colour coded on corresponding UMAP visualizations of the data.

### ATAC-seq analysis

Trimmed FASTQ files were obtained from the Rockefeller University’s Genome Resource Center and aligned to the mouse reference genome (UCSC release mm39) using Burrows-Wheeler Aligner (BWA, v.0.7.18), using BWA-MEM with default parameters. The output.sam files were name-sorted and duplicate reads were marked and removed using SAMtools (v.1.17)^83^. Peaks were called on each replicate using MACS3 (v.3.0.0) using the callpeak command, BAMPE, and a mappable genome estimate of 1.87 × 10^9^ (from the ENCODE pipeline). The fraction of reads in peaks was calculated using bedTools (v. 2.31.0)^84^ and used to scale bigwig files equivalently in deepTools (v.2.0.0)^85^. Bigwig files were created from deduplicated, pooled replicate bam files using deepTools, and normalized as reads per genome coverage. Pooled replicate bigwig files were also used to calculate peak coverage matrices to plot heatmaps of centred differential peaks, extended by 1 kb upstream and downstream. Differential peak analysis was done in DESeq2, using read count matrices across each individual replicate from concatenated, merged union peak sets from each replicate. These union peak sets were created separately for in vivo samples and in vitro samples. Differential analysis used negative binomial modelling, and Wald’s test for significance. To assign peaks to nearest expressed gene, part of the Inferelator-prior (v.0.3.8)^86^ package was used. Peaks were assigned to genes if they fell 50 kb upstream or 5 kb downstream of the gene body and were curated for expression using either scRNA-seq (in vivo samples) or bulk RNA-seq (in vitro samples). To make sure that all potential enhancers for genes related to the apoptotic cell clearance programme were identified, any unassigned intergenic peaks within approximately 200 kb of phagocytosis-related genes were manually curated. If no genes were expressed transcriptionally in the interval between phagocytic gene and unassigned intergenic peak, the intergenic peak was considered a potential enhancer for said gene. Peaks of interest were visualized using the integrated genome viewer (IGV) software (v.2.13.2), together with.bed files of differential peaks.

Motif enrichment analysis for in vivo samples was performed in two ways: First, the MEME suite (v. 5.5.2) package XSTREME^87^ in web browser format was used to search for motifs enriched in differential peaks, using as background the union set of all peaks detected, and the JASPAR 2022 vertebrate CORE transcription factor motif database, with lengths of 6–18 bp specified. Both known and de novo enriched motifs were collapsed to clusters based on similarity and ranked based on adjusted *P* value. Second, the transcription factor occupancy prediction by investigation of ATAC-seq signal (TOBIAS, v.0.14.0)^88^ framework was used to perform chromatin footprinting analysis. In brief, replicate-pooled bam files read coverage across the genome was calculated and corrected for Tn5 transposase cutting bias before footprint scores were calculated within the union set of called peaks. TOBIAS footprint scores were used to compute differential binding between anagen and catagen pooled replicates, or between *Rxra* wild-type and cKO pooled replicates. RXR-family catagen bound footprints were visualized in IGV by pooling each individual RXR-family member’s bed footprint file.

### CUT&RUN sequencing analysis

Trimmed FASTQ files were obtained from the Rockefeller University’s Genome Resource Center and aligned to the mouse reference genome (UCSC release mm39) using Burrows-Wheeler Aligner (BWA), using BWA-MEM with default parameters. The output.sam files were name-sorted and duplicate reads were marked and removed using SAMtools (v.1.17)^83^ Reads were filtered to less than 121 bp using SAMtools (v.1.3.1). BAM files for each replicate were combined using Samtools. Bigwig files were generated using Deeptools (v.3.1.2) with reads per kilobase of transcript per million mapped reads (RPKM) normalization and presented with Integrative Genomics Viewer software. CNR peaks were called using SEACR (v.1.3)^89^ from bedGraph files generated from RPKM-normalized Bigwig files (bigWigToBedGraph, UCSC Tools) using stringent setting and a numeric threshold of 0.01.

### Statistics and reproducibility

All data from every experiment were included for analysis unless an error was detected via failed positive or negative controls; in that case the entire experiment was excluded from analysis. Measurements were taken from independent distinct samples, unless stated otherwise. Statistical methods were not used to predetermine sample size. Experiments were not randomized or blinded, given the lack of ambiguity in phenotypes observed and internal controls used.

Statistical and graphical analyses were performed in Jupyter Notebooks, running a custom Python environment built as described in the single cell sequencing analysis section. Sample sizes, replicates and statistical tests used are indicated in each figure legend. Unless otherwise stated, unpaired two-tailed Student’s *t*-tests with a 95% confidence interval were performed to test for pair-wise differences among the means. Data are visualized as box-and-whisker plots, with the box representing the first to third quartiles of the data set, the median line inside the box, and the whiskers extending a maximum of 1.5 times the inter-quartile range. Observations that fall outside this range are plotted independently. For clarity, each observation in a data set is also visualized as a point overlaid on the box plot. Whenever representative plots or images are shown, data sets with similar results were generated from additional *n* > 3 independent biological replicates, from separate litters of mice or two independent cell culture experiments from separate days. All attempts at replication in this study were successful. In general, experiments were not randomized or performed in a blinded manner, due to the complex genetic models and obvious phenotypic differences in samples.

### Reporting summary

Further information on research design is available in the Nature Portfolio Reporting Summary linked to this article.

## Online content

Any methods, additional references, Nature Portfolio reporting summaries, source data, extended data, supplementary information, acknowledgements, peer review information; details of author contributions and competing interests; and statements of data and code availability are available at 10.1038/s41586-024-07855-6.

## Supplementary information

Supplementary Figures This file contains Supplementary Figs. 1–7.

Reporting Summary

Supplementary Table 1 Significantly up-regulated genes in catagen versus late anagen HFSCs (Log_2_[Fold Change]). Based on scRNA-sequencing data, genes which are significantly differentially expressed between catagen and late anagen HFSCs, given as Log_2_[Fold Change] and padj value.

Supplementary Table 2 Gene Set Enrichment Analysis (GSEA) terms for differential genes from anagen to catagen HFSCs. Based on scRNA-sequencing data, gene set enrichment analysis performed on differentially expressed transcripts increased in catagen versus late anagen HFSCs.

Supplementary Table 3 Extended motif analysis of differential peaks between catagen and late anagen. MEME-suite based analysis of transcription factor motifs enriched in catagen HFSCs-associated accessible chromatin peaks versus those motifs more strongly associated with late Anagen HFSCs open chromatin.

Supplementary Table 4 Significantly up-regulated genes in corpse-exposed *Rxra* wild type and conditional knockout HFSCs *in vitro* (Log_2_[Fold Change]). DESeq2-based differential gene expression analysis between corpse-exposed *Rxra* wild-type cKO HFSC lines in culture.

Supplementary Table 5 Significantly up-regulated genes in wild type HFSCs *in vitro* exposed to corpses with or without phosphatidylcholine cleavage (Log_2_[Fold Change]). DESeq2-based differential gene expression analysis between wild type cultered HFSCs exposed to corpses with (+Veh) or without phosphotidylcholine cleavage to release free fatty acids and LPC (+BEL).

Supplementary Table 6 DNA oligonucleotides used for sgRNA and qPCR analysis. Sequences of the DNA oligonucleotides used for sgRNA and qPCR analysis.

## Source data

Source Data Fig. 1

Source Data Fig. 2

Source Data Fig. 3

Source Data Fig. 4

Source Data Fig. 5

Source Data Extended Data Fig. 1

Source Data Extended Data Fig. 3

Source Data Extended Data Fig. 4

Source Data Extended Data Fig. 5

Source Data Extended Data Fig. 6

Source Data Extended Data Fig. 7

Source Data Extended Data Fig. 8

Source Data Extended Data Fig. 10

## Data Availability

All data supporting the findings of this study are available within the Article and its Supplementary Information. All single-cell, ATAC, CUT&RUN and bulk sequencing data generated within this study have been deposited at the Gene Expression Omnibus (GEO) under the super-series accession code GSE271007. Publicly available single-cell RNA sequencing data sets for telogen HFSCs (GSE90848) and AnaI–II HFSCs (GSE130850) were used. Source data are provided with this paper.

## Code availability

Custom code for scRNA-seq for this study is deposited at Zenodo (https://zenodo.org/records/12520073 (ref. ^90^)).
