# Supplementary material for: Stem cells tightly regulate dead cell clearance to maintain tissue fitness
Source: Nature. 2024 Aug 21;633(8029):407–16. doi: 10.1038/s41586-024-07855-6 (PMC11390485; doi:10.1038/s41586-024-07855-6)
Supplement: Supplementary file 1 — This file contains Supplementary Figs. 1–7. [file 41586_2024_7855_MOESM1_ESM.pdf]

nature portfolio

https://doi.org/10.1038/s41586-024-07855-6

Supplementary information

Stem cells tightly regulate dead cell clearance to maintain tissue fitness

In the format provided by the authors and unedited

Supplementary Information for

Stem cells tightly regulate dead cell clearance to maintain tissue fitness

Katherine S Stewart[1, ‡], Merve Deniz Abdusselamoglu[1], Matthew T Tierney[1], Anita Gola[1], Yun Ha Hur[1†], Kevin AU Gonzales[1†], Shaopeng Yuan[1†], Alain R Bonny[1], Yihao Yang[1†], Nicole R Infarinato[1†], Christopher J Cowley[1†], John M Levorse[1†], Hilda Amalia Pasolli[2], Sourav Ghosh[3], Carla V Rothlin[4], & Elaine Fuchs[1, ‡]

Affiliations

[1]Howard Hughes Medical Institute, Robin Chemers Neustein Laboratory of Mammalian Cell Biology and Development, The Rockefeller University, New York, NY, USA

[2]Electron Microscopy Resource Center, The Rockefeller University, New York, NY, USA

[3]Departments of Neurology and Pharmacology, Yale School of Medicine, New Haven, CT, USA

[4]Departments of Immunobiology and Pharmacology, Yale School of Medicine, New Haven, CT, USA

‡For reagents and queries, contact: fuchslb@rockefeller.edu; lab@kstewart.org

† *Present Addresses:* Department of Life Sciences, Pohang University of Science and Technology, Pohang, Republic of Korea (Y.H.H); Department of Discovery Technology and Genomics, Novo Nordisk Research Centre Oxford, Oxford, UK (K.A.G); Altos Labs, Cambridge Institute of Science, Granta Park, Cambridge, UK (S.Y.); Altos Labs, San Diego, CA, USA (Y.Y.); PrecisionScienta, Yardley, PA, USA (N.I.); Molecular Pharmacology Program, Memorial Sloan Kettering Cancer Center, New York, NY, USA (C.J.C.); Cardiovascular Research Group, Temple University, Philadelphia, PA, USA (J.L.)

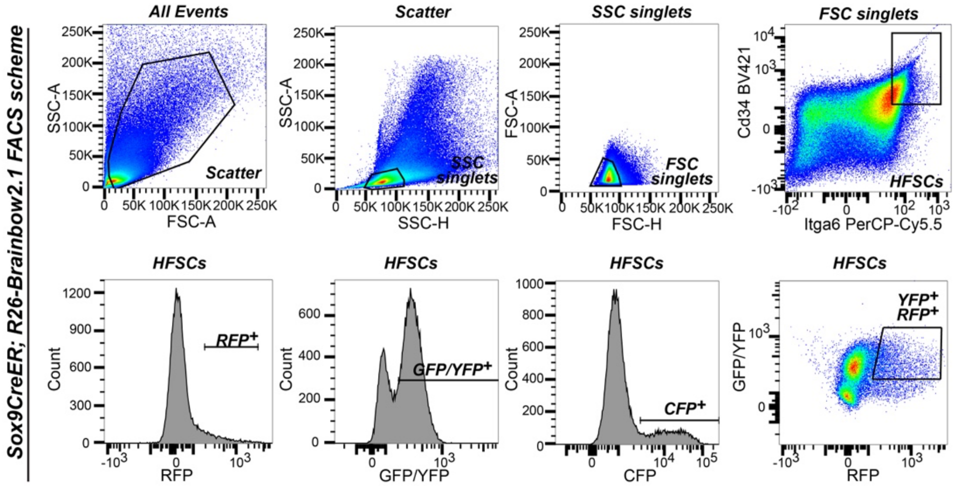

Supplementary Fig. 1 FACS gating strategy to isolate functional phagocytic cells (related to Fig. 1 and Extended Data Figs. 1 and 3)**.** Representative flow cytometry panel to isolate and analyze single and double-positive hair follicle stem cells (HFSCs) from *Sox9CreER*$^{+}$**;** *R26-Brainbow2.1* mice. Following stringent gating on singlets, HFSCs were identified as CD34+, Itga6+ epithelial cells that expressed at least one of RFP, GFP/YFP, or CFP. Note that our flow cytometer was unable to separate YFP and GFP. Double fluorophore positive cells arise from apoptotic cell engulfment events. *Tyro3/Axl/Mertk* (TAM)-family receptor (AlexaFluor700) and lysosome (LysoTracker DeepRed) expression was gated after identification of single/double-fluorophore+ cells.

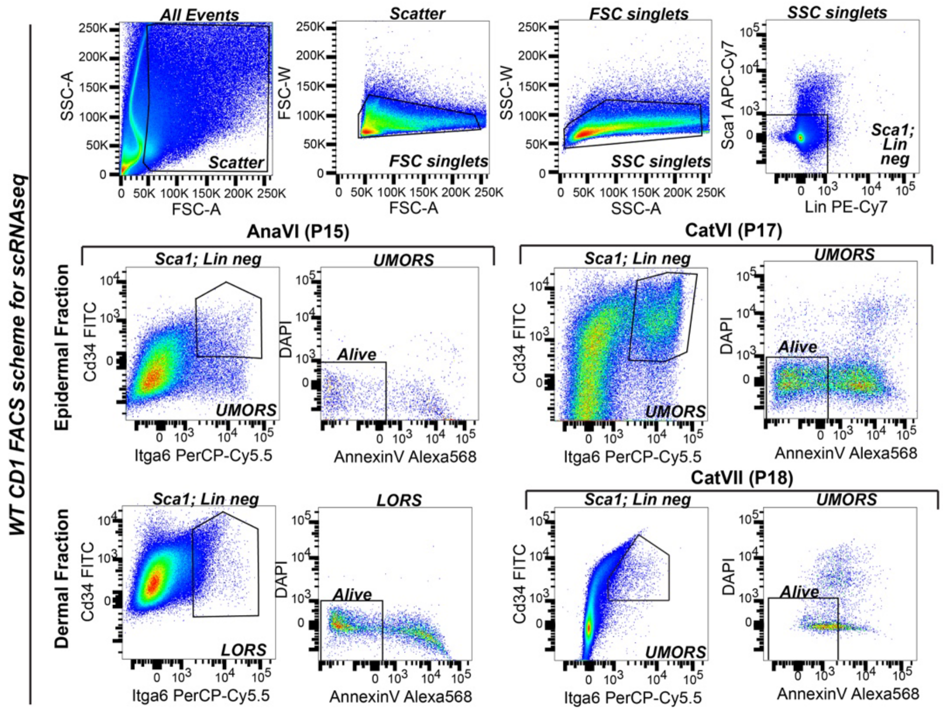

Supplementary Fig. 2 FACS gating strategy to isolate wild type HFSCs for scRNA-seq libraries from late anagen and mid-late catagen (related to Fig. 1 and Extended Data Figs 1 and 2)**.** Representative flow cytometry panel to isolate and analyze wild type hair follicle stem cells (HFSCs) during the hair cycle. Following gating on singlets, we excluded Sca1-positive lineage-positive (CD31/CD117/CD140a/CD45) cells. The double negative population was further gated on $CD34^{+}$, $Itga6^{+}$ outer root sheath (ORS) epithelial cells. Alive cells in each population were $AnnexinV^{-}$ $DAPI^{-}$. Upper and lower hair follicle epithelial cells were mechanically separated in epidermal and dermal fractions during the FACS preparation.

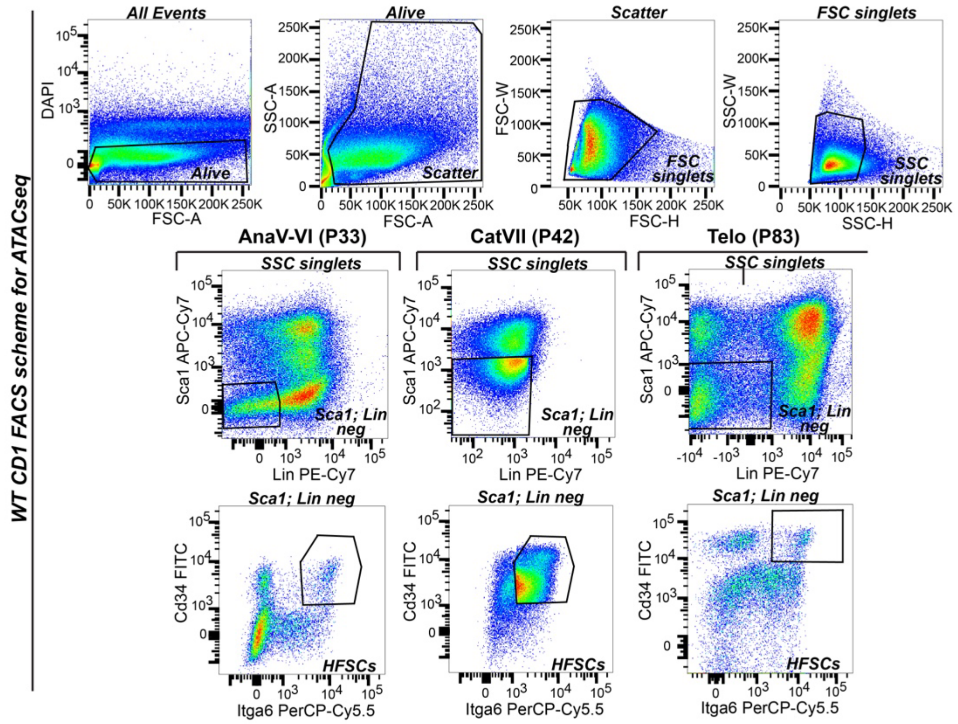

Supplementary Fig. 3 FACS gating strategy to isolate wild type HFSCs *in vivo* samples (related to Fig. 2 and Extended Data Figs. 3,8, and 10)**.** Representative flow cytometry panel to isolate and analyze wild type hair follicle stem cells (HFSCs) during the hair cycle used to isolate cells for ATAC-sequencing and for analysis of TAM-family phagocytic receptor and/or lysosome expression, or AldeFluor activity across the hair cycle. DAPI was used to exclude dead cells prior to gating on singlets. Singlet gating was followed by exclusion of Sca1-positive lineage-positive (CD31/CD117/CD140a/CD45) cells. The double negative population was further gated on CD34^{high}, Itga6^{+} hair follicle stem cells (HFSCs). For analysis of TAM-family phagocytic receptor and/or lysosome expression across the hair cycle, HFSCs were subsequently gated on on TAM-family AlexaFluor700 versus LyoTracker DeepRed (as done for DTA experiment in next figure). For AldeFluor activity, HFSCs were gated on AldeFluor as shown in Extended Data Fig. 8. Note, a similar gating strategy (following the CatVII panels) was used to isolate *Rxra* wild type (WT) and conditional knockout (cKO) HFSCs for ATAC-seq, as well as *in vitro* culture. Both *Rxra* WT (*Rxra*$^{+/+}$) and cKO (*Rxra*$^{fl/fl}$) were Sox9CreER^{+} YFP$^{fl/+}$ so CD34-FITC was replaced with YFP+ gated against Itga6-Percp-Cy5.5. Similarly, analysis of *Rxra* HFSCs for phagocytic program expression was done by subsequently gating on on TAM-family AlexaFluor700 versus LyoTracker DeepRed.

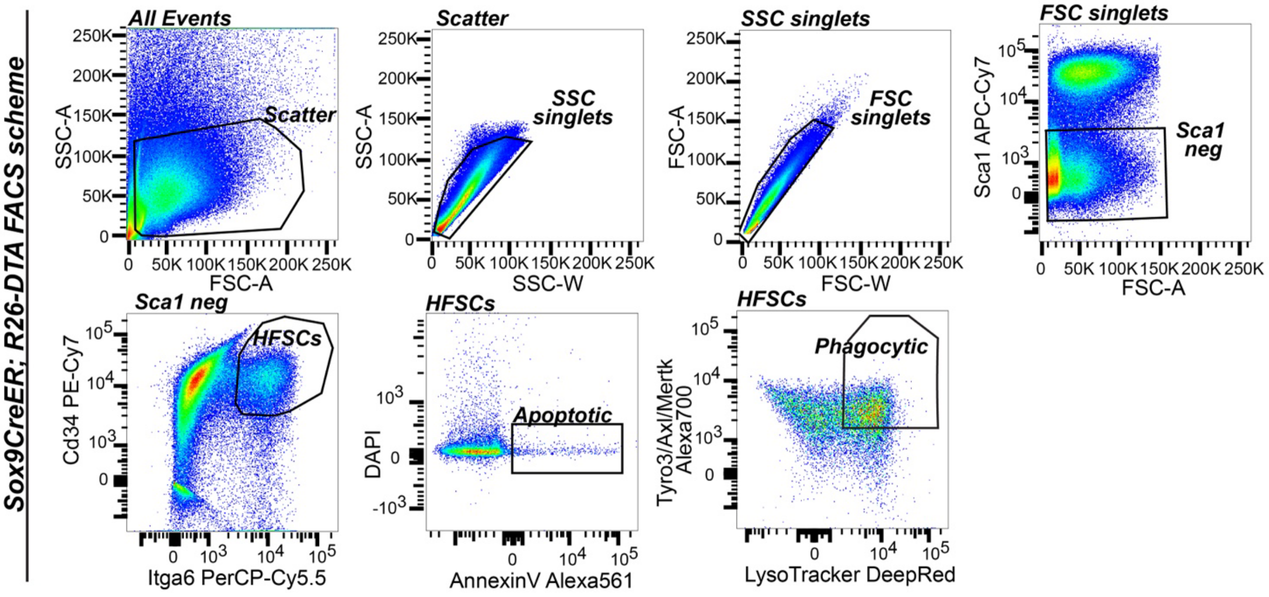

Supplementary Fig. 4 FACS gating strategy to isolate HFSCs from *Sox9CreER*$^{+}$; *R26-DTA* mice (related to Fig. 3)**.** To analyze telogen hair follicle stem and progenitor cells upon induction of ectopic corpses, we sorted Sca1^{-} epithelial cells from the back skin of *Sox9CreER*$^{+}$; *R26-DTA* mice. Cells were further gated for CD34^{+} Itga6^{+} hair follicle stem and progenitor cells. We assessed the extent of apoptotic corpse induction by AnnexinV^{+}, DAPI^{-} staining, and the phagocytic program by expression of Tyro3/Axl/Mertk (TAM)-family receptors and lysosome stain, LysoTracker DeepRed. Note, similar gates on TAM-family AlexaFluor700 versus LyoTracker DeepRed gates were used in combination with Sca1 APC-Cy7, Lineage (Lin) PE-Cy7, CD34-FITC and Itga6-PercpCy5.5 on wild-type FACS-isolated HFSCs to profile phagocytic program expression for Extended Data Fig. 4b-d.

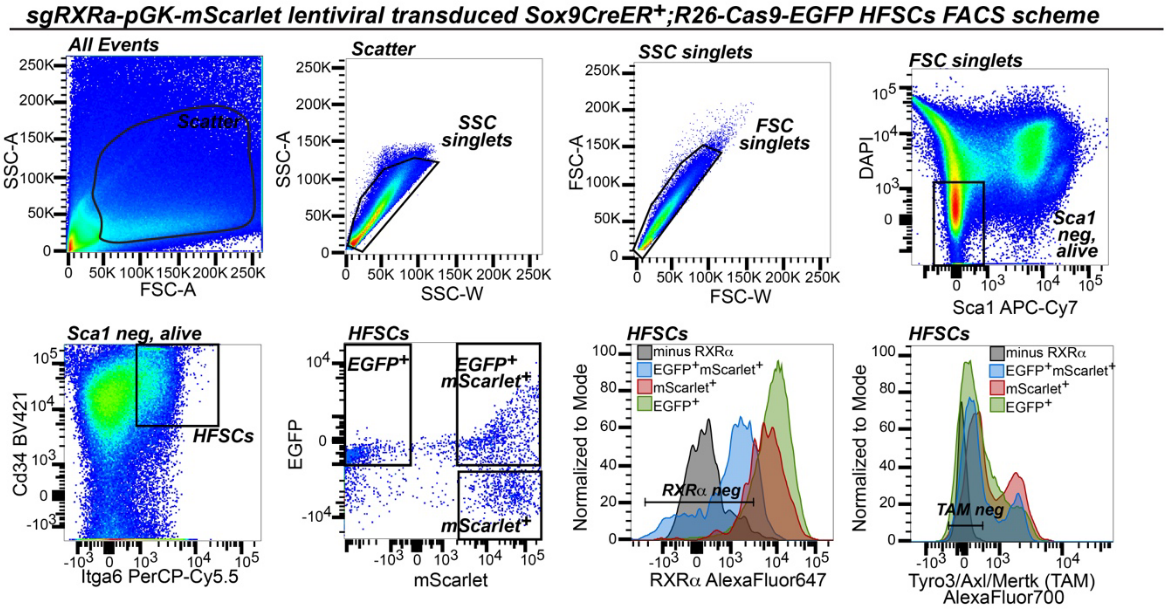

Supplementary Fig. 5 FACS gating strategy to isolate mosaically deleted *Rxra* HFSCs or to profile *Cyp26b1*- over expression HFSCs or RXRa over expression HFSCs from late catagen (related to Extended Data Fig. 5, Fig.4, and Fig. 2 respectively). To analyze RXRα and TAM-family receptor levels on HFSCs in a mosaic deletion model, we excluded cells that were DAPI+ (dead) and Sca1+ (upper HF and interfollicular epidermis). We selected for CD34+ Itga6+ hair follicle stem and progenitor cells in catagen, and then gated GFP+ (Cas9-expressing), mScarlet+ (sgRXRa expressing) and GFP+ mScarlet+ (expressing both Cas9 and sgRXRa). Fluoresecent minus one controls were used to set RXRα negative and TAM-family negative gates. Note, a similar gating strategy was used to profile TAM-family expression on virus infected control (RFP+) versus Cyp26b1 overexpressing (YFP+ RFP+) HFSCs. YFP replaces EGFP and RFP replaces mScarlet expression in the FACS plots above, and RXRa wasn't examined for this set of experiments. Finally, a similar gating strategy was used to profile RXRa overexpressing HFSCs from *K14rtTA* mice transduced with TRE-RXRa-Myc; pGK-H2BRFP. To do so, EGFP was replaced with Itga6 (so that Itga6 was repeated) and gated against H2BRFP (instead of mScarlet). RFP+ and RFP- gates were further examined for expression of RXRa and TAM-receptors as indicated.

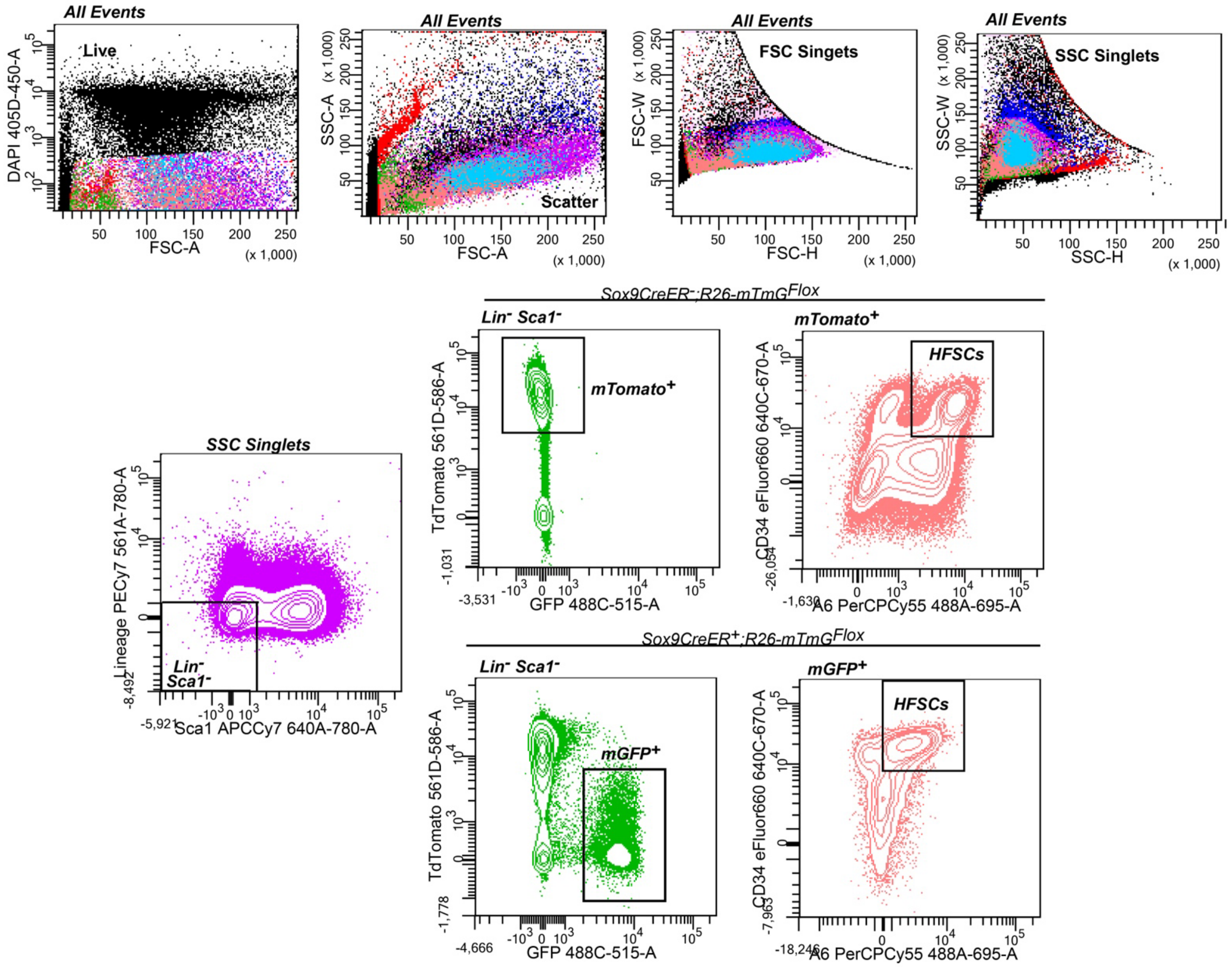

Supplementary Fig. 6 FACS gating strategy to isolate HFSCs from *Sox9CreER; R26-mTmG* second telogen mice for primary culture (related to Figs. 3, 4, 5 and Extended Data Figs. 3, 6, 7, 10). Representative flow cytometry panels to generate mTomato+ and mGFP+ primary HFSC lines for culture. To isolate mTomato^{+} or mGFP^{+} HFSCs, after gating on DAPI- singlets, we excluded Lineage (CD117/CD140a/CD31/CD45)$^{+}$ Sca1^{+} cells, and further gated for mTomato^{+} or mGFP^{+} cells from *Sox9CreER- or Sox9CreER*+ mice, respectively. mTomato^{+} or mGFP^{+} cells were further selected on CD34^{High} Itga6^{+} gates.

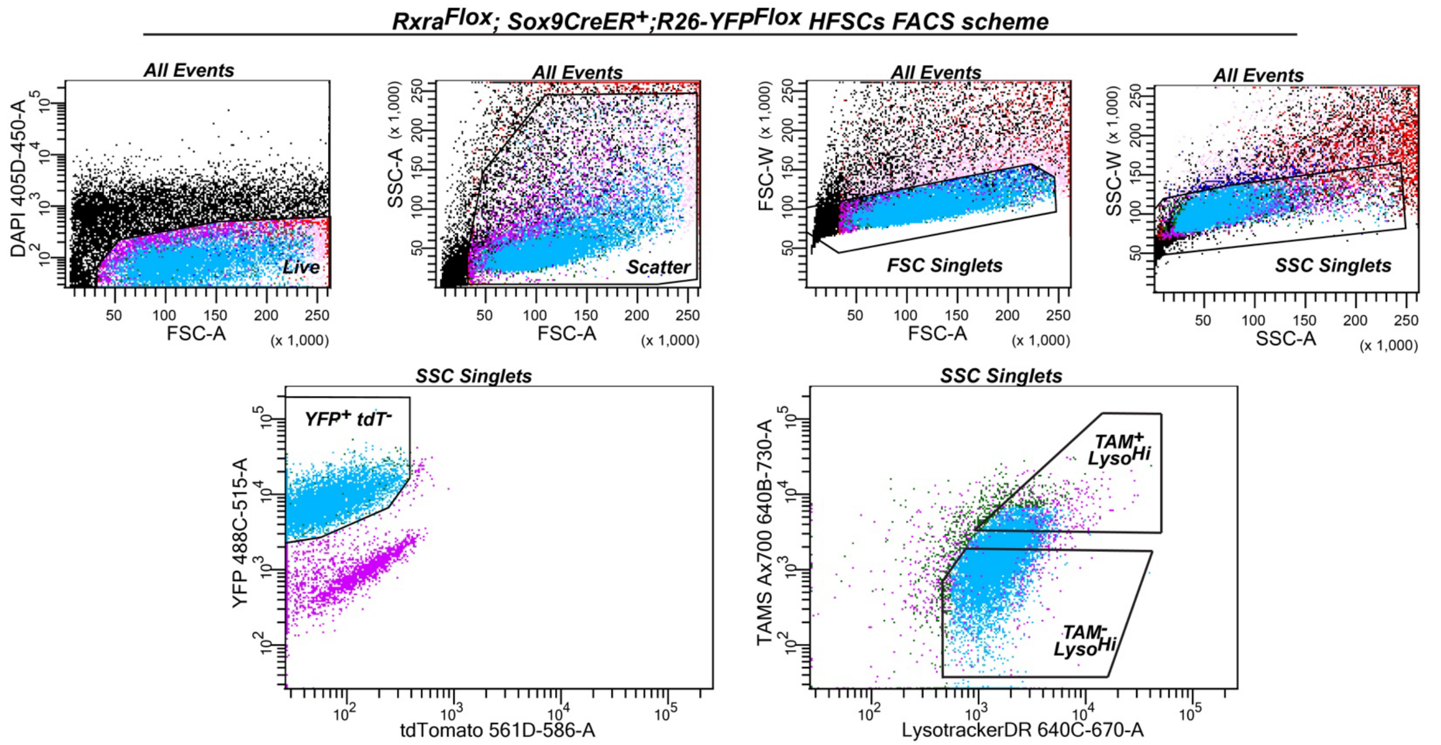

Supplementary Fig. 7 FACS gating strategy to assess phagocytic program expression in *YFP*+ (or *GFP*+) HFSCs fed mTomato+ corpses in culture (related to Figs. 3, 4, 5 and Extended Data Figs. 3, 6, 7, 10). Representative flow cytometry panels for analysis of phagocytic program expression and number of corpse-containing phagocytic HFSCs in culture. Cell lines were first gated as DAPI- (alive) singlets. This was followed by YFP+ (for *Rxra* WT or cKO cell lines) or GFP+ (for Sox9CreER+;mTmG-floxed cell lines) mTomato- gates to select for recipient (phagocytic) HFSCs, before gating TAM-family+ against Lysotracker high. For each experiment, fluorescent minus one controls for TAMs and LysoTracker were used to set determine negative staining for each. Note, a similar scheme was used to determine the fraction of corpse-containing phagocytic HFSCs, with a stringent YFP+ mTomato+ or GFP+ mTomato gate determining the percentage of double positive.
