# Supplementary material for: Stem cells tightly regulate dead cell clearance to maintain tissue fitness
Source: Nature. 2024 Aug 21;633(8029):407–16. doi: 10.1038/s41586-024-07855-6 (PMC11390485; doi:10.1038/s41586-024-07855-6)
Supplement: Supplementary file 2 — Reporting Summary [file 41586_2024_7855_MOESM2_ESM.pdf]

nature portfolio

Corresponding author(s): Elaine Fuchs; Katherine Stewart

Last updated by author(s): 06/23/2024

Reporting Summary

Nature Portfolio wishes to improve the reproducibility of the work that we publish. This form provides structure for consistency and transparency in reporting. For further information on Nature Portfolio policies, see our Editorial Policies and the Editorial Policy Checklist.

Statistics

For all statistical analyses, confirm that the following items are present in the figure legend, table legend, main text, or Methods section.

n/a	Confirmed	
☐	☒	The exact sample size (*n*) for each experimental group/condition, given as a discrete number and unit of measurement
☐	☒	A statement on whether measurements were taken from distinct samples or whether the same sample was measured repeatedly
☐	☒	The statistical test(s) used AND whether they are one- or two-sided<br>*Only common tests should be described solely by name; describe more complex techniques in the Methods section.*
☐	☒	A description of all covariates tested
☐	☒	A description of any assumptions or corrections, such as tests of normality and adjustment for multiple comparisons
☐	☒	A full description of the statistical parameters including central tendency (e.g. means) or other basic estimates (e.g. regression coefficient) AND variation (e.g. standard deviation) or associated estimates of uncertainty (e.g. confidence intervals)
☐	☒	For null hypothesis testing, the test statistic (e.g. *F*, *t*, *r*) with confidence intervals, effect sizes, degrees of freedom and *P* value noted<br>*Give P values as exact values whenever suitable.*
☒	☐	For Bayesian analysis, information on the choice of priors and Markov chain Monte Carlo settings
☒	☐	For hierarchical and complex designs, identification of the appropriate level for tests and full reporting of outcomes
☒	☐	Estimates of effect sizes (e.g. Cohen's *d*, Pearson's *r*), indicating how they were calculated

Our web collection on statistics for biologists contains articles on many of the points above.

Software and code

Policy information about availability of computer code

Data collection	BD FACSDiva (v. 8.0) for FACS sorting; Zen (v 3.1) or Andor Fusion (v 2.3) for microscopy image collection
Data analysis	FlowJo (v.9.0) for flow cytometry analysis; ImageJ (v. 2.9.0) and Imaris (v. 10.1) for immunofluorescence analysis;<br>STAR (v2.6), Salmon (v.1.4.0), R (v.3.6.1), RStudio (v.3.4.2), Tximport (v.1.12.3), DESeq2 analysis (v.1.16.1), Pandas (v.2.0.1), NumPy (v.1.24.2), SciPy (v.1.10.1), scikit-learn (v.1.2.0), SCANPY (v1.9.3), AnnData (v0.9.1), matplotlib (v3.7.1), seaborn (v.0.13.1),GSEA software (v.4.3.2), Burrows-Wheeler Aligner (BWA, v.0.7.18), SAMtools (v.1.17 and v.1.3.1), MACS3 (v.3.0.0), bedTools (v. v. 2.31.0), deepTools (v.2.0.0 and (v.3.1.2), Inferelator-prior (v.0.3.8), IGV software (v.2.13.2), MEME suite (v. 5.5.2), TOBIAS (v.0.14.0), SEACR (v. 1.3) for bulk RNA-seq, ATAC-seq and scRNA-seq; code for scRNA-seq deposited in Zenodo: https://zenodo.org/records/12520073

For manuscripts utilizing custom algorithms or software that are central to the research but not yet described in published literature, software must be made available to editors and reviewers. We strongly encourage code deposition in a community repository (e.g. GitHub). See the Nature Portfolio guidelines for submitting code & software for further information.

Data

Policy information about availability of data

All manuscripts must include a data availability statement. This statement should provide the following information, where applicable:

- Accession codes, unique identifiers, or web links for publicly available datasets
- A description of any restrictions on data availability
- For clinical datasets or third party data, please ensure that the statement adheres to our policy

All data supporting the findings of this study are available within the Article and its Supplementary Information. All single-cell, ATAC, Cut&Run and bulk sequencing data generated within this study have been deposited at the Gene Expression Omnibus (GEO) under accession code GSE230523. Publicly available single-cell RNA sequencing data sets for telogen HFSCs (GSE90848) and anagen I-II HFSCs (GSE130850) were used. Source data are provided with this paper. All sequencing data were aligned to the mm39 reference genome (UCSC). Gene set enrichment analysis (GSEA) used the MSigDB 2022 mouse database. Transcription factor motif analysis used the JASPAR 2022 vertebrate core transcription factor motif database.
mm39: https://genome.ucsc.edu/cgi-bin/hgGateway?db=mm39
MSigDB 2022: https://www.gsea-msigdb.org/gsea/msigdb/mouse/genesets.jsp?collection=GO
JASPAR 2022: https://jaspar2022.genereg.net/

Research involving human participants, their data, or biological material

Policy information about studies with human participants or human data. See also policy information about sex, gender (identity/presentation), and sexual orientation and race, ethnicity and racism.

Reporting on sex and gender	N/A
Reporting on race, ethnicity, or other socially relevant groupings	N/A
Population characteristics	N/A
Recruitment	N/A
Ethics oversight	N/A

Note that full information on the approval of the study protocol must also be provided in the manuscript.

Field-specific reporting

Please select the one below that is the best fit for your research. If you are not sure, read the appropriate sections before making your selection.

[x] Life sciences [ ] Behavioural & social sciences [ ] Ecological, evolutionary & environmental sciences

For a reference copy of the document with all sections, see nature.com/documents/nr-reporting-summary-flat.pdf

Life sciences study design

All studies must disclose on these points even when the disclosure is negative.

Sample size	Preliminary experiments were performed when possible to determine requirements for sample size, taking into account resources available and ethical, reductionist animal use. For all experiments a minimum of n=3 independent samples per condition were used, except for Sox9CreER;R26-DTA-floxed, where only 2 Sox9CreER- wild type littermate control mice were available to contrast against n=4 Sox9CreER+ experimental mice in telogen (Fig 3b). Notably, other data panels show multiple additional wild type telogen mice from other experiments that replicate two Sox9CreER- wild type littermate controls in all parameters assessed.
Data exclusions	No data were excluded from analysis, except in the case of (1) lentiviral injection of sgRXRa-mScarlet into Sox9CreER;R26-Cas9-GFP mice or (2) lentiviral injection of TRE-RXRa-Myc;pGK-RFP into K14rtTA mice. There animals which didn't transduce at least 5,000 hair follicle stem cells were excluded from FACS analysis assessed by lentiviral transduction reporters (mScarlet+ or RFP+ cells, respectively). Data were pre-excluded due to the prior established difficulty of assessing phagocytic receptor expression phenotype reliably by FACS on low cell numbers.
Replication	Experiments were performed on n=3 mice, and preferably 6-12 mice, of each genotype or condition. Experiments were performed in batches of 3 animals per genotype per hair cycle stage, and repeated at least twice, if not up to 4 separate times. For cell culture experiments measuring RXRa+,RARg+, cJun+, FosB+, or pSTAT3+ HFSCs by immunofluorescence, technical duplicates were performed and averaged across biological replicates, with at least 2 biological replicates per experiment. Experiments were successfully replicated at least twice on separate days and replicates were pooled for analysis. For cell culture experiments measuring TAM+Lysosome-high or phagocytic HFSCs by FACS, experiments were performed with 2 or 3 biological replicates per experiment. Experiments were successfully replicated at least twice on separate days and replicates were pooled for analysis (unless otherwise indicated in Figure legend). All attempts at replication were successful.

Randomization	No randomization was used beyond the random nature of Mendelian genetics. For the small molecule intradermal injections, no randomization was used because each animal received contralateral inhibitor and vehicle control injections in the backskin.
Blinding	In general experiments were not blinded to the investigator due to the obvious nature of the apoptotic cell clearance phenotypes, and the complex genetic models used which required genotyping prior to experiments being conducted. Whenever possible, phenotypes were assessed without referring to the animal id (which indicated genotypes); this was possible for FACS analysis of RXRa and TAM receptor surface levels on (1) K14rtTA; RXRa overexpression experiments, (2) Rxra-floxed;Sox9CreER;R26-YFP experiments, and (3) Sox9CreER;R26-DTA experiments.

Reporting for specific materials, systems and methods

We require information from authors about some types of materials, experimental systems and methods used in many studies. Here, indicate whether each material, system or method listed is relevant to your study. If you are not sure if a list item applies to your research, read the appropriate section before selecting a response.

Materials & experimental systems

n/a	Involved in the study
☐	☒ Antibodies
☐	☒ Eukaryotic cell lines
☒	☐ Palaeontology and archaeology
☐	☒ Animals and other organisms
☒	☐ Clinical data
☒	☐ Dual use research of concern
☒	☐ Plants

Methods

n/a	Involved in the study
☒	☐ ChIP-seq
☐	☒ Flow cytometry
☒	☐ MRI-based neuroimaging

Antibodies

Antibodies used	For immunofluorescence: rabbit anti-cleaved-caspase-3 (Cell Signaling, 9661, 1:250), rat anti-RFP (Chromotek, 5F8, 1:1000), rabbit anti-RFP (MBL, PM005, 1:1000), chicken anti-GFP/YFP (Abcam, ab13970, 1:1000), goat anti-P-cadherin (R&D, AF761, 1:250), rabbit anti-keratin14 (Fuchs laboratory, 1:200), rabbit anti-keratin24 (Fuchs laboratory, 1:200), sheep anti-Ki67 (Novus Biologicals, AF7649, 1:200), rabbit anti-Myc epitope (71D10) (Cell Signaling, 2278, 1:250), rat biotinylated anti-CD45 (Biolegend, 5530, 1:200), rabbit anti-RXRa (D6H10) (Cell Signaling, 3085, 1:250), rabbit anti-RARg (D3A4) (Cell Signaling, 8965, 1:250), rabbit anti-MFGE8 (Invitrogen, PA5-109955, 1:200), rat AlexaFluor647-conjugated anti-F4/80 (BM8) (Biolegend, 123121, 1:200), rat biotinylated anti-Itga6/CD49f (GoH3) (Biolegend, 313603, 1:500), rabbit anti-cJun (60A8) (Cell Signaling, 9165, 1:250), rabbit anti-FosB (5G4) (Cell Signaling, 2251, 1:250), and rabbit anti-phospho-STAT3 (Tyr705)(D3A7) (Cell Signaling, 9145, 1:250). All secondary antibodies used were raised in a donkey host, and conjugated to AlexaFluor488, Rhodamine, or AlexaFluor647 (Jackson ImmunoResearch Laboratory; 1:500). Catalog numbers (given in order of: AlexaFluor488, Rhodamine, and AlexaFluor647 conjugates) for donkey anti-rabbit antibodies (711-545-152; 711-295-152; 711-605-152), for donkey anti-rat antibodies (712-545-150; 712-295-150; 712-605-150), for donkey anti-chicken antibodies (703-545-155; 703-295-155; 703-605-155), for donkey anti-goat antibodies (705-545-003; 705-295-003; 705-605-003), and for donkey anti-sheep AlexaFluor647 (713-605-003). TUNEL staining used the Cell Death Detection Kit (TMR Red or FITC, from Roche). For multiplexed immunofluorescence: (Panel 1) rat anti-Foxp3-AlexFluor488 (FJK-16s) (ThermoFisher, 53-5773-82, 1:100), In situ cell death detection kit, TMR red (Roche), rat anti-CD8-AlexaFluor647 (BioLegend, 100724, 1:150); (Panel 2) rat anti-CD206-AlexaFluor488 (MMR) (BioLegend,141710, 1:500) and rat anti-CD68-AlexaFluor647 (BioLegend, 137004, 1:500); (Panel 3) rat anti CD11c-AlexaFluor488 (N418) (BioLegend, 117311, 1:100) and rat anti-Ly6g-AlexaFluor647 (1A8) (BioLegend, 127610, 1:150); (Panel 4) rat anti-Itga6/Cd49f-AlexaFluor488 (BioLegend, 313608, 1:150) and rat anti-Langerin-AlexaFluor647 (929F3.01) (Novus Biologicals, DDX0362A647-100; 1:100); (Panel 5) rat anti F4/80-AlexaFluor488 (BioLegend, 123122, 1:150) and rat anti-CD172a (Sirp-AlexaFluor647 (BioLegend, 144028, 1:150); (Panel 6) hamster anti-TCRgd-AlexaFluor488 (BioLegend, 118128, 1:100) and rat anti-Tim4-AlexaFluor647 (RMT4-54) (BioLegend, 130008, 1:150); (Panel 7) rat anti-CD4-AlexaFluor488 (RM4-5) (BioLegend, 100529, 1:100) and rat anti-CD3-AlexaFluor647(17A2) (BioLegend, 100209, 1:100); (Panel 8) Avidin-FITC (ThermoFisher Scientific, A821 1:1000) and rat anti-I-A/I-E (MHCII)-AlexaFluor647 (M5/114.15.2) (BioLegend, 107618, 1:150); (Panel 9) rat anti-CD45-AlexaFluor488 (BioLegend, 103122, 1:150) and rat anti-P-cadherin-AlexaFluor647 (R&D Systems, FAB761R-100UG, 1:200).<br><br>For FACS: Primary antibodies were used as follows: rat biotinylated anti-CD45 (30-F11) (eBioscience, Cat #13-0451-82, 1:200), rat biotinylated anti-CD117 (2B8) (eBioscience, 13-1171-82, 1:200), rat biotinylated anti-CD140a (APA5) (eBioscience, 13-1401-82, 1:200), rat biotinylated anti-CD31 (390) (eBioscience, 13-0311-82, 1:200), rat anti CD34-FITC (RAM34) (eBioscience, 11-0341-82, 1:200), rat anti CD34-eFluor660 (RAM34) (eBioscience, 50-0341-82,1:200), rat anti CD49f/Itga6-PercpCy5.5 (GoH3) (BioLegend, 313617, 1:250), rat anti-Ly6A/E(Sca1)-APC-Cy7(BioLegend, 108125, 1:1000), rabbit anti-RXR (D6H10) (CST, 3085, 1:250), rat anti-Tyro3/Dtk-AlexaFluor700 (R&D Systems, FAB759N, 1:200), rat anti-Mertk-AlexaFluor700 (R&D Systems, FAB5912N, 1:200), and rat anti-Axl-AlexaFluor700 (R&D Systems, FAB8541N, 1:200). Secondary antibodies were used as follows: Strepavidin-PE-Cy7 (1:3000) and donkey AlexaFluor 488 or AlexaFluor568 (1:500). AnnexinV-AlexaFluor568 (Invitrogen, A13202, 1:100) and/or DAPI was used to identify apoptotic and dying cells, respectively.
Validation	All primary antibodies used were validated by the manufacturer for the antigen specificity and purpose using knockout cell lines for western blotting, immunofluorescence, or FACS , and/or western blotting against recombinant protein for each specific antigen. Validation statements are available on manufacturer's websites for each catalog number. Antibodies were further validated in lab using knockout tissue or cell lines whenever possible, eg. RXRa, Mertk, Keratin 24 and Keratin 14.

Eukaryotic cell lines

Policy information about cell lines and Sex and Gender in Research

Cell line source(s)	Primary hair follicle stem cell (HFSC) lines were FACS-isolated as described in the methods section from male Sox9CreER;mTmG mice, using the gating strategy shown in the supplemental figures. Rxra WT and cKO HFSCs were FACS-isolated as described in the methods and supplemental figures, using female mice. The mouse fibroblast 3T3/J2 line originated in Howard Green's laboratory, was transferred directly to Elaine Fuchs lab (no commercial source), and has been passaged in the Fuchs' laboratory since.
Authentication	All HFSC lines, as well as the 3T3/J2 fibroblast line, were functionally and morphologically validated as HFSC or fibroblast lines respectively.
Mycoplasma contamination	The cell lines used in the manuscript were not directly tested for mycoplasma contamination, but were passaged and maintained in a tissue culture facility that is routinely tested for mycoplasma (especially via the feeder cell lines). All cell lines and media preparations tested negative for mycoplasma contamination.
Commonly misidentified lines (See ICLAC register)	None of the cell lines used in this study are found in the ICLAC register.

Animals and other research organisms

Policy information about studies involving animals; ARRIVE guidelines recommended for reporting animal research, and Sex and Gender in Research

Laboratory animals	All laboratory mice were used for experiments between 2 and 10 weeks of age, except for lentiviral injections, performed at embryonic day 9.5 (as described in methods). previously generated mouse lines were used in this study: RxraFlox (ref.55; Jax stock 013086), Sox9CreER (ref.56), Krt14-rtTA (ref.57; Jax stock 008099), Rosa26lox-STOP-lox-YFP (ref.58; Jax stock 006148; referred to as R26YFP), Rosa26mTmG (ref.59; Jax stock 007576; referred to as R26mTmG), Rosa26Brainbow2.1 (ref. 60; Jax stock 013731, referred to as R26Brainbow2.1), Rosa26lox-STOP-lox-Cas9-EGFP (ref.61; Jax stock 026175, referred to as R26Cas9-EGFP), Rosa26lox-STOP-lox-DTA (ref.62; Jax stock 010527, referred to as R26DTA) and Mertk-/- (full knockout, ref.63). The Mertk knockout mice used in this study are referred to as Mertk-/-V2 in the originating paper. Wild type CD1 or C57BL/6 animals were originally purchased from Charles River and The Jackson Laboratories, respectively, and maintained as in house colonies
Wild animals	No wild animals were used in the study.
Reporting on sex	Mice were sexed at genotyping (P8-10) and confirmed by visual inspection of genitalia as adults. For comparative assessments of phenotype between control and mutant animals, age and sex matched mice were used, with preference given to littermate controls wherever possible. Male and female mice have different hair cycle lengths due to a longer telogen quiescence phase in females, but otherwise progress through the hair cycle similarly. In addition to sex, strain and individuals also affect hair cycle stages. Therefore, we always determine hair cycle stage by visual inspection, and morphological staging on sectioned tissue. Beyond the difference in hair cycle timing, no differences in morphological or molecular mechanism of apoptotic elimination of the hair follicle during catagen exist, and thus both male and female animals were used in the study. Data presented is aggregated from both sexes, but was checked by plotting male and female animals separately prior to aggregation. No effect of sex was noted on any parameter measured in this study.
Field-collected samples	No field-collected samples were used in the study.
Ethics oversight	Mice were maintained and bred under specific-pathogen-free conditions at the Comparative Bioscience Center (CBC) at The Rockefeller University, an Association for Assessment and Accreditation of Laboratory Animal Care (AALAC)-accredited facility. All mouse protocols were approved by the Institutional Animal Care and Use Committee (IACUC) at The Rockefeller University, or by the IACUC at Yale University.

Note that full information on the approval of the study protocol must also be provided in the manuscript.

Flow Cytometry

Plots

Confirm that:

- [x] The axis labels state the marker and fluorochrome used (e.g. CD4-FITC).
- [x] The axis scales are clearly visible. Include numbers along axes only for bottom left plot of group (a 'group' is an analysis of identical markers).
- [x] All plots are contour plots with outliers or pseudocolor plots.
- [x] A numerical value for number of cells or percentage (with statistics) is provided.

Methodology

Sample preparation	To obtain single-cell suspensions for fluorescence activated cell sorting (FACS) at all stages of the hair cycle, back skin was excised, and the dermal side scraped with a dull scalpel to remove excess fat prior to incubation with 0.25% collagenase

(Sigma-Aldrich) in warm PBS, dermal side down for 45-60 min at 37oC with gentle rotation in a plastic petri dish. The dermal side was scraped gently with a dull scalpel to mechanically dissociate cells in the lower outer root sheath (ORS) and hair bulb ("dermal fraction"). The dermal fraction was only kept for late anagen and early-mid catagen samples, and was processed separately from the epidermal fraction. To collect the epidermal fraction, the skin was placed dermal side down in 0.25% trypsin-EDTA (Gibco) for 20-25 min at 37oC with gentle rotation. The hairy side of the skin was scraped against the direction of hair growth with a dull scalpel to release cells in the upper HF (including the HF bulge stem and hair germ progenitor cells). For both dermal and epidermal fractions, the resulting cell suspensions were pipetted up and down with a 5ml serological pipette for 5 minutes, before being quenched with FACS buffer (5% fetal bovine serum, FBS, in PBS). Plastic petri dishes were rinsed with 5ml of FACS buffer 2-3 times, which was collected and added to the appropriate cell suspension. Suspensions were filtered through sequential 70um and 40 um nylon filters (VWR), before being pelleted at 350xg for 15 min at 4oC. Cell pellets were resuspended in ice cold FACS buffer, re-filtered into FACS tubes, and incubated with primary antibodies for 20 min on ice. Secondary antibodies and LysoTracker DeepRed (Invitrogen, 1:4000) were added directly to FACS tubes, and incubation continued for 10 min on ice. Samples were further diluted with FACS buffer plus DNase (Roche) to minimize cell clumping prior to sorting or analysis. Alternatively, cultured HFSCs were trypsinized for 7-10min (as for passaging the cell lines), and pelleted at 300xg before resuspension, filtering and incubating with primary antibodies.

Instrument

Sorting was performed on a BD FACSAriaII

Software

FACSDiva software for running FACS machine

Cell population abundance

HFSCs were generally obtained as 1-10% of the isolated population from adult back skin. When isolated from tissue culture, they comprised 50-100% of the population (depending on confluency at sorting). Phagocytic HFSCs generally ranged from 5-40% of the HFSC population.

Gating strategy

Cells were generally gated as DAPI- (alive) prior to sorting for forward and side scatter, then singlets for both forward and side scatter. Lineage+ (CD45/CD117/CD31/CD140a) and Sca1+ (upper hair follicle and interfollicular epidermis) were excluded prior to gating on CD34+ Itga6/CD49f+ HFSCs. Where possible, genetically encoded fluorescent reporters were used to further refine HFSC identification. See supplemental figures 1-5 for example gating strategies for each type of experiment. To assess (1) RXRa, (2) TAM-family, (3) Lysosomes and (4)corpse engulfment both single-positive controls and fluorescent minus one controls for each marker were included with every experiment. These were used to determine positive and negative staining gates.

☒ Tick this box to confirm that a figure exemplifying the gating strategy is provided in the Supplementary Information.
